# Genome-wide identification and expression analysis of the GRAS gene family in *Dendrobium chrysotoxum*

**DOI:** 10.3389/fpls.2022.1058287

**Published:** 2022-11-28

**Authors:** Xuewei Zhao, Ding-Kun Liu, Qian-Qian Wang, Shijie Ke, Yuanyuan Li, Diyang Zhang, Qinyao Zheng, Cuili Zhang, Zhong-Jian Liu, Siren Lan

**Affiliations:** ^1^ College of Forestry, Fujian Agriculture and Forestry University, Fuzhou, China; ^2^ Key Laboratory of National Forestry and Grassland Administration for Orchid Conservation and Utilization at College of Landscape Architecture, Fujian Agriculture and Forestry University, Fuzhou, China

**Keywords:** GRAS gene, *Dendrobium chrysotoxum*, phylogenetic analysis, abiotic stress, expression analysis

## Abstract

The GRAS gene family encodes transcription factors that participate in plant growth and development phases. They are crucial in regulating light signal transduction, plant hormone (e.g. gibberellin) signaling, meristem growth, root radial development, response to abiotic stress, etc. However, little is known about the features and functions of GRAS genes in Orchidaceae, the largest and most diverse angiosperm lineage. In this study, genome-wide analysis of the GRAS gene family was conducted in *Dendrobium chrysotoxum* (Epidendroideae, Orchidaceae) to investigate its physicochemical properties, phylogenetic relationships, gene structure, and expression patterns under abiotic stress in orchids. Forty-six *DchGRAS* genes were identified from the *D. chrysotoxum* genome and divided into ten subfamilies according to their phylogenetic relationships. Sequence analysis showed that most *DchGRAS* proteins contained conserved VHIID and SAW domains. Gene structure analysis showed that intronless genes accounted for approximately 70% of the *DchGRAS* genes, the gene structures of the same subfamily were the same, and the conserved motifs were also similar. The *K*a/*K*s ratios of 12 pairs of *DchGRAS* genes were all less than 1, indicating that *DchGRAS* genes underwent negative selection. The results of *cis*-acting element analysis showed that the 46 *DchGRAS* genes contained a large number of hormone-regulated and light-responsive elements as well as environmental stress-related elements. In addition, the real-time reverse transcription quantitative PCR (RT−qPCR) experimental results showed significant differences in the expression levels of 12 genes under high temperature, drought and salt treatment, among which two members of the LISCL subfamily (*DchGRAS13* and *DchGRAS15*) were most sensitive to stress. Taken together, this paper provides insights into the regulatory roles of the GRAS gene family in orchids.

## Introduction

In recent years, studies on the GRAS gene family have shown that it plays an important role in plant growth and development and particularly in the response to abiotic stress (Jaiswal et al., 2022). The GRAS gene family name is derived from the abbreviations of the earliest three members: gibberellic acid insensitive (GAI) (Peng et al., 1997), repressor of GAI (RGA) (Silverstone et al., 1998), and scarecrow (SCR) (Laurenzio et al., 1996). Genes of the GRAS gene family generally consist of 400–770 amino acids, with a changeable N-terminal sequence and a more conservative C-terminal sequence (Niu et al., 2016a). The C-terminal sequence has five conserved motifs: LHR I, LHR II, VHIID, PFYRE and SAW. Among them, VHIID, PFYRE, and SAW are more conserved (Pysh et al., 1999). GRAS gene family has been identified and analysed in many plants. According to the difference in N-terminal length and sequence, members of GRAS gene family can be divided into 17 subfamilies at most (DELLA, PAT1, LISCL, HAM, SCR, SHR, LAS, SCL3 in model plants, and NSP1, NSP2, RAD1, SCLA, SCLB, RAM1, DL, SCL4/7, SCL32 in other plants.) (Tian et al., 2004; Liu and Widmer, 2014; Cenci and Rouard, 2017).

First, in the growth and development of plants and morphological regulation, through the regulation of the LAS subfamily of the GRAS protein family, the formation of mutants prevents the development of axillary meristems, which is more conducive to the growth of meristems (Schumacher et al., 1999). *OsMOC1* and LAS are homologous genes that are key factors in regulating tillering in rice (Liang et al., 2014). SHR induces the activity of SCR promoters in specific tissues (Helariutta et al., 2000), which play an important role in radial growth of roots and stems (Laurenzio et al., 1996). *NSP1* (Smit et al., 2005) and *NSP2* (Kalo et al., 2005) also play important roles in nodulation signalling in legumes. Second, in terms of plant hormone signal transduction, the introduction of an early stop codon in the PAT1 1 mutant indicated that the PAT1 protein inhibits phyA signalling (Bolle et al., 2000); *AtSCL13* plays a positive regulatory role in phyB signalling (Torres-Galea et al., 2006); and the DELLA subfamily and SCL3 subfamily antagonize each other and regulate GA (gibberellin) signal transduction in plants, thereby regulating plant seed germination, stem elongation, and flower development (Heoa et al., 2011). In addition, GRAS proteins also play an important role in biotic and abiotic stresses. *OsCIGR1* and *OsCIGR2*, members of the PAT1 subfamily, can inhibit the infection of *Oryza sativa L.* pathogens (Day et al., 2004), and their expression in *Arabidopsis thaliana* can improve the drought, cold, and salt resistance of plants (Yuan et al., 2016). The DELLA protein interacts with the cellular gibberellin (GA) receptor *GID1*, activates stress adaptation mechanisms by altering cellular reprogramming, and regulates plant growth under different adversities by combining plant intrinsic signal transduction. *AtSCL9*, *AtSCL13*, *AtSCL14*, and other genes can all respond to abiotic stress (Torres-Galea et al., 2006; Fode et al., 2008; Lee et al., 2008).

Orchidaceae is one of the largest angiosperm families, accounting for 10% of the world’s angiosperms and representing an ideal taxa for studying biodiversity and evolution. Orchids have abundant flower morphology and pollination mechanisms (Ramirez et al., 2011; Li et al., 2020; Lu et al., 2022), unique epiphytic phenomena, xerophytic physiology (Zhang et al., 2016), and extremely complex mycorrhizal relationships (Li et al., 2022). *Dendrobium* is the second largest genus of Orchidaceae and has high medicinal and ornamental value and strong stress resistance (Zhang et al., 2016; Zhang et al., 2021a). *D. chrysotoxum* is a valuable medicinal plant resource in Orchidaceae, and its functional components, such as erianin (Zhang et al., 2021b) and chrysotoxene (Dou et al., 2018), have been reported. The stout pseudobulb of *D. chrysotoxum* is not only rich in medicinal components but also closely related to its resistance to stress (Zhang et al., 2021a). Although GRAS genes have been extensively studied in model plants as well as in some crops, little is known about the characterization of GRAS genes in orchids. The advent of high-quality, chromosomal-level orchid genomes has made it possible to explore the role of the GRAS gene family in orchids. Exploring the regulatory mechanism of the GRAS gene family in the growth and development of *D. chrysotoxum* and the expression of functional genes under abiotic stress is of great significance for the development and utilization of *D. chrysotoxum*.

In this study, GRAS gene family members of *D. chrysotoxum* were examined by gene structure analysis, phylogenetic tree construction, selection pressure analysis, *cis*-acting element analysis, and analysis of expression patterns under abiotic stress. This study provides a reference for further research on the evolutionary relationship and functional characteristics of the GRAS family in orchids.

## Materials and methods

### Data sources

We downloaded the genome sequence and annotation files of *D. chrysotoxum* (Zhang et al., 2021a) from the National Center for Biotechnology Information (NCBI) (accession number: PRJNA664445) and the GRAS protein sequence files of *A. thaliana* and *O. sativa* from PlantTFDB (http://planttfdb.gao-lab.org ) and Phytozome v13 (https://phytozome-next.jgi.doe.gov/ ) respectively.

### Identification and physicochemical properties of the GRAS gene family

A local BLASTp search was conducted with *A. thaliana* GRAS proteins as a probe (built-in Tbtools; Chen et al., 2020). In addition, the conserved domains of GRAS, PF03514, were downloaded from the online database (http://pfam.xfam.org/) (Finn et al., 2014) to perform the HMMER search (default parameters) to screen for candidate genes of the GRAS gene family in *D. chrysotoxum* (Eddy, 2011). BLAST and HMMER results were combined to remove incomplete and redundant protein sequences, and uncertain genes were uploaded to the NCBI website (https://blast.ncbi.nlm.nih.gov/) for a BLASTp search. The online software ExPASy (https://www.expasy.org/) was used to analyse the protein length, isoelectric point (pI), molecular weight (MW), hydrophilic large average (GRAVY), instability index (II) and fat index (AI) of the protein (Duvaud et al., 2021). Snapgene is used to analyse the length of CDS. The number of exons and chromosome distribution of the *DchGRAS* genes were obtained from the gff3 files of *D. chrysotoxum*. Subcellular localization was predicted by Plant mPloc (Chou and Shen, 2010).

### Phylogenetic analysis of GRAS genes

The sequences of 46 GRAS proteins of *D. chrysotoxum*, 34 GRAS proteins of *A. thaliana*, and 60 GRAS proteins of *O. sativa* were introduced into the MEGA 7.0 software. We used the muscle program and default settings for multisequence alignment and the maximum likelihood method (ML; bootstrap method: 1000) to construct the phylogenetic tree (Edgar, 2004). FigTree and online software Evloview (http://www.evolgenius.info/evolview/treeview) were used to improve and beautify the phylogenetic tree.

### Gene structure analysis and gene distribution on chromosomes

Using the CDD tool in the online NCBI software, the conserved domains of the *DchGRAS* protein were predicted. The conserved motif of the GRAS gene in *D. chrysotoxum* was analysed and downloaded by the online software MEME, and the prediction number was set to ten. TBtools was used to integrate phylogenetic trees, conserved protein motifs, and overall comparative maps of gene structure.

The software TBtools was used to extract the location information of the GRAS gene from the genome file and gene annotation file of *D. chrysotoxum* and to construct the physical map of the GRAS gene on the chromosome.

### Calculation of *K*a and *K*s ratios

According to the phylogenetic tree, the gene pairs with a close relationship were selected. DNAMAN software was used to select gene pairs with greater than 60% identity. TBtools software was then used to calculate *K*a (nonsynonymous rate), *K*s (synonymous substitution), and *K*a/*K*s (evolutionary constraint) values. Divergence time (T) was calculated using the form T=*Ks*/(2×9.1×10^−9^)×10^−6^ million years ago. In general, *K*a/*K*s<1.0 represents purifying or negative selection, *K*a/*K*s = 1.0 represents neutral selection, and *K*a/*K*s>1.0 represents positive selection (Wang et al., 2021).

### Promoter element analysis of *DchGRAS* genes

To identify putative *cis*-acting elements in the promoter, we used TBtools to obtain 2,000 bp gene sequence upstream of the promoter codon from the *D. chrysotoxum* genome (Chen et al., 2020; Zhang et al., 2021a). The online software PlantCARE (https://bioinformatics.psb.ugent.be/webtools/plantcare/html/) (Lescot et al., 2002) was used to analyse the *cis*-acting regulatory elements in the promoter region of the *DchGRAS* gene (Chen et al., 2021). Then, the data was processed using Excel software, followed by TBtools and Origin software for visualization.

### Treatment of plant materials

The plant materials used in this study were gotten the shade house in Forest Orchid Garden of Fujian Agriculture and Forestry University. We selected nine pots of mature *D. chrysotoxum* with the same growth periods and culture conditions in the shed and put them into the artificial climate culture room and divided them into three groups equally (group-A, group-B and group-C). After they were cultured for one week (photoperiod: 16h light/8h dark, temperature period: 15°C/25°C), the leaves of three groups of plants taken as contrast samples (CK-A, CK-B, CK-C).

Then, three groups of plants were subjected with three different abiotic stress treatments respectively. The plants of group-A were placed under the conditions of 16h light/8h dark, 30°C/38°C, watered once every 24h, and collected mixed samples of leaves after 48h treatment (T); Under the condition of 16h light/8h dark, 15°C/25°C, the plants of group-B were irrigated with 0.5 M NaCl solution in their roots every 8h. After 48h of continuous irrigation for 48h, the leaves of group-B were mixed and sampled (St); The leaves of group-C plants were also collected together as a sample after ten days, starting from the substrate humidity in the basin drops below 1.0 under the conditions of 16h light/8h dark, 15°C/25°C (10) (Cao et al., 2015; Zeng et al., 2019).

### RT−qPCR analysis

Using the Biospin Plant Total RNA Extraction Kit (Bioer Technology, Hangzhou, China), RNA was isolated from the leaves of *D. chrysotoxum*. First-strand DNA was synthesized with TransScript^®^ All-in-One First-Strand cDNA Synthesis SuperMix for quantitative PCR (qPCR; TransGen Biotech, Beijing, China). TransScript^®^ All-in-One First-Strand cDNA Synthesis SuperMix for qPCR was also used to remove genomic DNA. The real-time reverse transcription quantitative PCR (RT−qPCR) primers for *DchGRAS* were designed by Primer Premier 5 software (**Supplementary Table 5**). Primer blast on the NCBI website was used to confirm primer specificity. PerfectStart™ Green qPCR SuperMax (TransGen Biotech, Beijing, China) was used for RT−qPCR analysis. The Ef-1α gene from *D. chrysotoxum* was used as the reference gene in this study (Hou et al., 2022). The relative expression of the target gene was calculated by the 2^-△△CT^ method (Wang et al., 2021). All the RT−qPCR analyses set three technical repetitions respectively.

## Results

### Identification and physicochemical properties of the GRAS gene family

Sequence alignment was carried out between the *A. thaliana* GRAS gene and the *D. chrysotoxum* genome. The obtained genes were analysed in the SwissProt database, and the genes with GRAS conserved domains were screened. We finally identified 46 GRAS genes of *D. chrysotoxum* and named the 46 GRAS genes *DchGRAS1*–*DchGRAS46* according to the order in which genes are distributed on chromosomes (from top to bottom). The physical and chemical properties of *D. chrysotoxum* were analysed by the ExPASY online tool. These *DchGRAS* protein sequences varied considerably in the number of amino acids (AA), ranging from 405 to 783 aa, with an average length of 574 aa. The isoelectric point (pI) ranged from 4.92 to 8.02, with four GRAS proteins having an isoelectric point greater than 7, making them alkaline, and 42 GRAS proteins having an isoelectric point less than 7, making them acidic. It is further predicted that four proteins (*DchGRAS7*, *DchGRAS19*, *DchGRAS35*, and *DchGRAS45*) are strongly acidic. The protein molecular weights ranged from 25.72 for *DchGRAS12* to 85.54 kDa for *DchGRAS13*, with an average molecular weight (Mw) of 57.43 kDa. The deduced grand average of hydrophilic (GRAVY) values were in the range of -0.568 for *DchGRAS14* to 0.201 for *DchGRAS5*, suggesting that most *DchGRAS* proteins were hydrophilic. The instability index (II) was in the range of 30.78 for *DchGRAS12* to 64.29 for *DchGRAS42*, and the aliphatic index (AI) of *DchGRAS-*deduced proteins was in the range of 72.70 for *DchGRAS33* to 105.44 for *DchGRAS12*. In addition, we also predicted the subcellular localization of 46 *DchGRAS* genes. The results showed that most of the genes were distributed in the nucleus, and a few genes were distributed in the cytoplasm (**Table 1**). These results imply that they may function in the nucleus similar to most transcription factors.

**Table 1:** Characteristics of the GRAS proteins from *D. chrysotoxum*.

Name	Gene ID	AA^1^ (aa)	pI^2^	Mw^3^ (kDa)	GRAVY^4^	II^5^	AI^6^	CDS^7^(bp)	Chromosome location^8^	Number of exons	Subcellular localization^9^
*DchGRAS1*	Maker103270	558	5.46	64.72	-0.011	50.76	90.29	1767	Chr02:26408192–26409958	1	Nucleus.
*DchGRAS2*	Maker109984	482	6.24	46.61	-0.047	45.07	95.07	1263	Chr03:1989689–1991459	5	Cytoplasm., Nucleus.
*DchGRAS3*	Maker109993	482	6.81	39.21	-0.112	47.43	95.06	1035	Chr03:2003069–2004103	1	Nucleus.
*DchGRAS4*	Maker90665	410	5.53	46.27	-0.094	47.95	85.66	1275	Chr04:77284065–77286015	2	Nucleus.
*DchGRAS5*	Maker107353	486	5.74	50.44	0.201	44.51	99.37	1428	Chr05:55087262–55088689	1	Extracell., Nucleus.
*DchGRAS6*	Maker107243	603	5.4	49.79	-0.293	58.22	75.72	1362	Chr05:55239506–55240867	1	Nucleus.
*DchGRAS7*	Maker109157	588	4.92	63.27	-0.256	51.16	83.39	1710	Chr05:58493522–58495231	1	Nucleus.
*DchGRAS8*	Maker106713	783	5.36	69.48	-0.190	61.33	87.15	1926	Chr06:22492694–22495775	2	Nucleus.
*DchGRAS9*	Maker109319	640	7.59	61.04	-0.127	40.67	89.89	1704	Chr06:90385613–90387380	2	Nucleus.
*DchGRAS10*	Maker96576	658	6.41	64.11	-0.392	49.1	77.78	1716	Chr06:93270893–93272608	1	Nucleus.
*DchGRAS11*	Maker64901	405	5.81	47.36	-0.090	60.06	91.81	1314	Chr07:51745669–51746982	1	Nucleus.
*DchGRAS12*	Maker77807	718	7.72	25.72	0.089	30.78	105.44	654	Chr08:30122565–30127357	2	Nucleus.
*DchGRAS13*	Maker93384	718	6.61	85.54	-0.560	54.67	76.07	2271	Chr09:1803507–1805777	1	Nucleus.
*DchGRAS14*	Maker74260	718	5.52	75.86	-0.568	55.11	76.55	3993	Chr09:90897477–90903204	2	Nucleus.
*DchGRAS15*	Maker74359	718	6.28	65.84	-0.561	51.38	75.82	1740	Chr09:90918703–90920442	1	Nucleus.
*DchGRAS16*	Maker102355	542	7.16	51.14	-0.420	55.01	74.89	1395	Chr10:8813808–8815202	1	Nucleus.
*DchGRAS17*	Maker108806	630	5.22	69.14	-0.148	46.26	77.81	1950	Chr10:12747107–12791396	1	Nucleus.
*DchGRAS18*	Maker120980	575	5.78	53.66	-0.040	43.64	91.76	1488	Chr10:13665155–13666642	1	Nucleus.
*DchGRAS19*	Maker120973	508	4.96	50.57	-0.043	39.79	86.17	1380	Chr10:13774703–13776082	1	Nucleus.
*DchGRAS20*	Maker74947	695	6.45	50.57	-0.303	43.65	86.75	1350	Chr10:20947846–20949195	1	Nucleus.
*DchGRAS21*	Maker116208	531	5.24	44.27	-0.190	50.90	82.62	1182	Chr10:50696148–50697329	1	Nucleus.
*DchGRAS22*	Maker55437	405	5.81	47.36	-0.090	60.06	91.81	1314	Chr11:63210270–63211583	1	Nucleus.
*DchGRAS23*	Maker57245	445	6.61	45.48	-0.208	49.52	87.94	1212	Chr12:16579699–16580910	1	Nucleus.
*DchGRAS24*	Maker56787	718	5.09	83.16	-0.545	49.01	74.22	2199	Chr12:27678250–27680448	1	Nucleus.
*DchGRAS25*	Maker34817	533	5.09	52.99	0.026	57.04	95.00	1467	Chr12:35790222–35791688	1	Nucleus.
*DchGRAS26*	Maker67092	558	5.85	72.72	0.012	53.23	89.56	1983	Chr13:57193721–57221035	2	Cytoplasm., Nucleus.
*DchGRAS27*	Maker18932	575	5.98	50.02	-0.025	44.75	86.35	1374	Chr14:3417648–3419021	1	Nucleus.
*DchGRAS28*	Maker98448	630	5.14	62.34	-0.163	52.52	83.71	1725	Chr14:44820072–44823800	1	Nucleus.
*DchGRAS29*	Maker91453	658	5.94	59.57	-0.396	52.77	89.75	1578	Chr14:44820072–44823800	4	Nucleus.
*DchGRAS30*	Maker78471	438	6.29	47.90	-0.118	51.75	90.52	1278	Chr15:22460806–22462083	1	Nucleus.
*DchGRAS31*	Maker09526	544	6.08	64.23	-0.287	46.73	84.32	1731	Chr15:27837037–27847016	2	Cytoplasm., Nucleus.
*DchGRAS32*	Maker71994	544	6.22	64.10	-0.295	46.76	83.49	1731	Chr15:28087128–28107888	2	Nucleus.
*DchGRAS33*	Maker53947	623	5.29	69.59	-0.235	51.28	72.70	1938	Chr16:11189178–11191115	1	Nucleus.
*DchGRAS34*	Maker53896	575	5.79	55.23	-0.172	50.81	84.64	1515	Chr16:11897962–11899476	1	Nucleus.
*DchGRAS35*	Maker53963	575	4.95	53.85	-0.154	42.08	79.84	1488	Chr16:12003067–12004554	1	Nucleus.
*DchGRAS36*	Maker06292	531	5.46	44.87	-0.288	50.91	78.85	1176	Chr16:26958259–26959434	1	Nucleus.
*DchGRAS37*	Maker59349	542	6.06	56.50	-0.390	43.20	73.94	1563	Chr17:87774189–87775751	1	Nucleus.
*DchGRAS38*	Maker59202	630	5.33	62.67	-0.184	51.80	83.47	1704	Chr17:90199821–90201524	1	Nucleus.
*DchGRAS39*	Maker59068	508	5.08	53.00	-0.125	46.5	82.17	1470	Chr17:91328084–91329553	1	Nucleus.
*DchGRAS40*	Maker64611	533	5.55	53.73	0.084	50.24	100.93	1479	Chr18:14440477–14443592	1	Nucleus.
*DchGRAS41*	Maker84688	544	5.82	57.91	-0.298	52.81	82.97	1569	Chr18:95039362–95040930	1	Nucleus.
*DchGRAS42*	Maker84792	410	5.44	45.04	-0.058	64.29	94.52	1230	Chr18:96739953–96741182	1	Nucleus.
*DchGRAS43*	Maker65831	640	8.02	73.00	-0.243	54.36	79.24	2067	Chr19:12929694–12934290	2	Nucleus.
*DchGRAS44*	Maker65692	558	5.73	77.52	-0.135	55.44	84.83	2133	Chr19:15485118–15487250	1	Nucleus.
*DchGRAS45*	Maker22635	588	4.92	63.31	-0.249	52.32	83.9	1710	Unknow	1	Nucleus.
*DchGRAS46*	Maker26072	658	5.65	51.18	-0.315	52.96	92.21	1371	Unknow	2	Nucleus.

^1^AA, exhibits amino acid; ^2^pI, theoretical isoelectric point; ^3^Mw, molecular weight; ^4^GRAVY, grand average of hydrophobicity; ^5^II, instability index; ^6^AI, aliphatic index; ^7^CDS, Snapgene is used to calculate the CDS length of genes; ^8^The location of the gene on the chromosome comes from the gff file; ^9^subcellular localization predicted by Plant-mPloc (Chou and Shen, 2010; Kaundal et al., 2010).

### Phylogenetic analysis of GRAS genes

**Figure 1:**
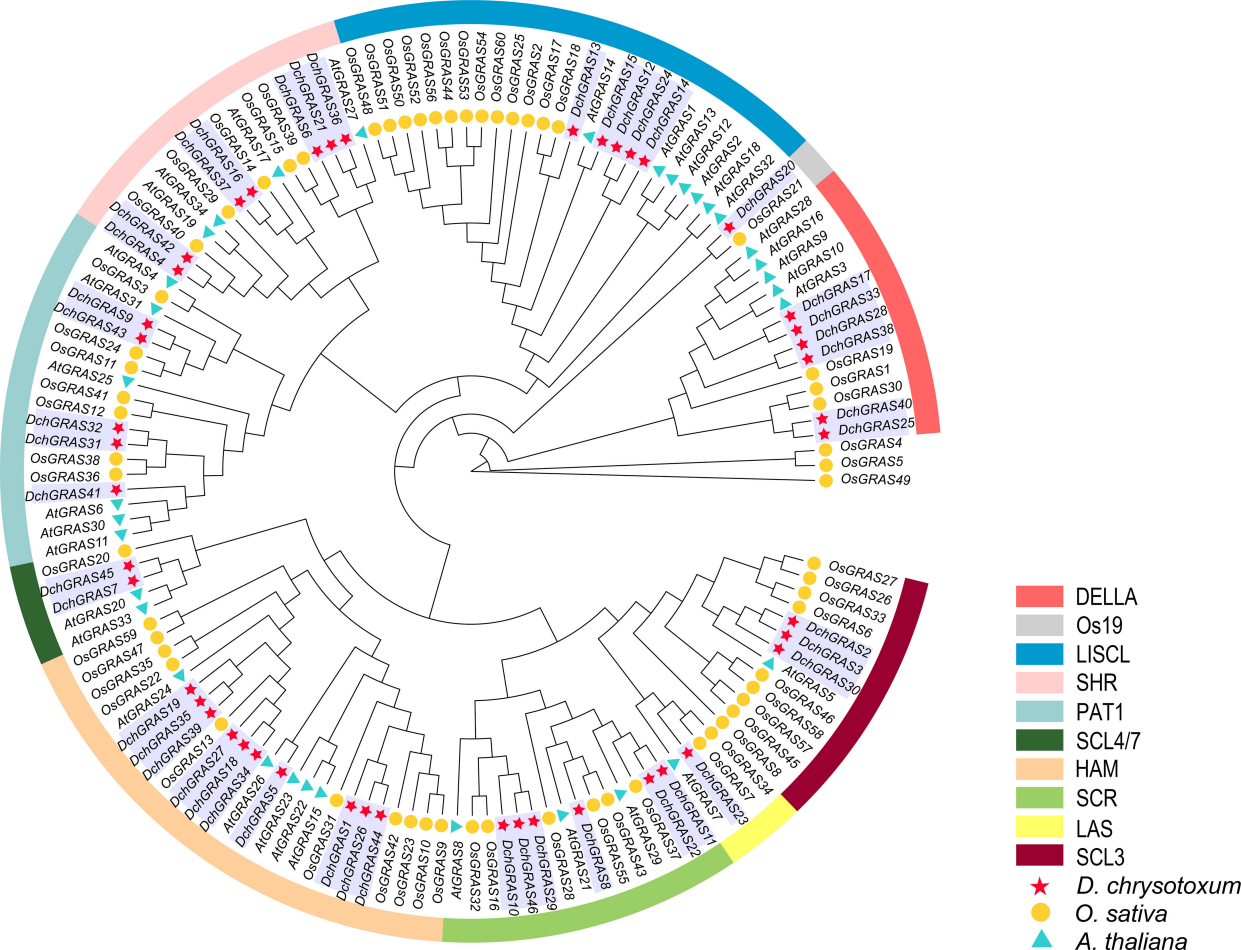
Phylogenetic tree of GRAS genes based on the GRAS protein sequences of *D. chrysotoxum*, *A. thaliana*, and *O. sativa*. The GRAS protein sequences of *D. chrysotoxum*, *A. thaliana*, and *O. sativa* are shown in **Supplementary Table 1**.

To reveal the evolutionary relationship of the *D. chrysotoxum* GRAS gene family and help with its classification, an evolutionary tree was constructed with 140 GRAS genes from *D. chrysotoxum*, *A. thaliana*, and *O. sativa*. We divided 46 GRAS genes in *D. chrysotoxum* into ten subfamilies: DELLA (six genes), Os19 (one gene), LISCL (five genes), SHR (seven genes), PAT1 (five genes), SCL4/7 (two genes), HAM (ten genes), SCR (six genes), LAS (one gene) and SCL3 (three genes) (**Figure 1**). Among them, the HAM subfamily has the most members, which includes ten genes; the Os19 and LAS subfamilies have the fewest members, both of which contain only one member.

### Gene structure analysis and gene distribution on chromosomes

**Figure 2:**
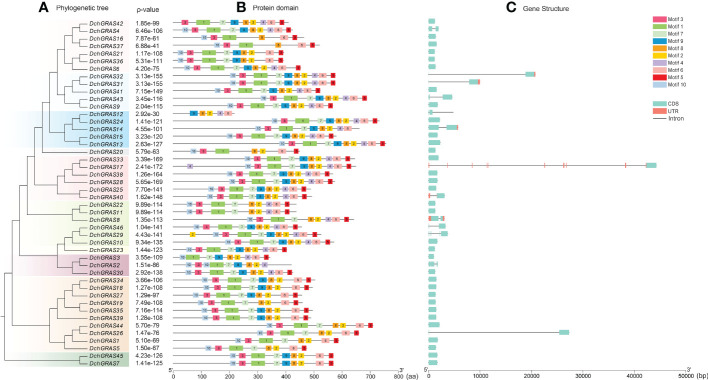
Analysis of the motif and gene structure of the GRAS gene family in *D. chrysotoxum*. **(A)** Using MEGA 7.0 software, the phylogenetic tree of 46 *DchGRAS* genes was constructed using the maximum likelihood method. **(B)** Determination of conserved motifs in the *DchGRAS* gene using default parameters on the MEME website. **(C)** Based on gff files, TBtool software was used to visualize the structure of genes. Motif1–10 sequence and logo in **Supplementary Figure 2**.

To further understand the gene structure of *D. chrysotoxum*, the conserved protein motifs were analysed on the MEME website and set as Motif1–Motif10. The results showed that most of the conserved motifs of the *DchGRAS* gene existed in the C-terminal domain and were arranged in the order of Motif10, Motif3, Motif1, Motif7, Motif9, Motif8, Motif2, Motif4, Motif6, and Motif5, with Motif8 being the most highly conserved. The conserved motifs of genes in the same subfamily are the same. Among them, the deletion of Motif4 and Motif9 occurred in members of the HAM, SCR, SHR, SCL4/7, and Os19 subfamilies. PAT1 and DELLA have a very stable conserved C-terminal domain, and no motif deletion exists in those subfamilies (**Figure 2B**).

By analysing the intron−exon structure, it was found that 69.59% of the genes in *D. chrysotoxum* had no introns, and only 14 genes had introns; among the genes with introns, *DchGRAS32*, *DchGRAS26* and *DchGRAS17* had longer introns (**Figure 2C**). The gene structures of the same subfamily are basically the same, and the conserved motifs also have high similarities (**Figure 2**). Among the 46 *DchGRAS* proteins, only *DchGRAS17* and *DchGRAS29* showed motif addition, and both genes contained introns. We also found that most genes without introns have different degrees of motif deletion.

**Figure 3:**
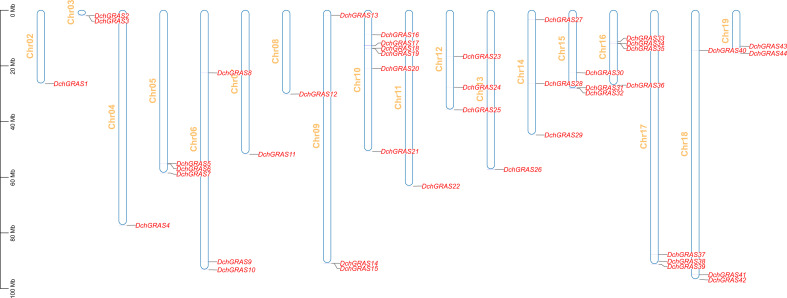
GRAS gene distribution on chromosomes of *D. chrysotoxum*.

The visualization of genes on chromosomes showed that 44 *DchGRAS* genes were distributed on 18 chromosomes, Chr02–Chr19, and the remaining two genes were distributed on unknown chromosomes (**Figure 3**). Chr10 contained the greatest number of *DchGRAS* genes, with six genes; Chr02, Chr04, Chr07, Chr08, Chr11, and Chr13 all contained only one *DchGRAS* gene.

### Calculation of *K*a and *K*s ratios

Calculating *K*a (nonsynonymous substitution), *K*s (synonymous substitution), and *K*a/*K*s (evolutionary selection pressure) is of great significance for determining phylogeny and understanding the evolutionary dynamics within (among) species (Wang et al., 2021). In this study, 12 pairs of *DchGRAS* genes with consistency greater than 60% were selected by DNAMAN software for calculation and analysis. The results showed that the *K*a/*K*s values of 12 pairs of *DchGRAS* genes were less than 1, indicating that almost all *DchGRAS* genes experienced negative selection (**Table 2**), which made them more evolutionarily stable. The divergence time of these 12 gene pairs was between 0.66 Mya for *DchGRAS45*–*DchGRAS7* and 117.98 Mya for *DchGRAS41*–*DchGRAS32*.

**Table 2:** *K*a/*K*s analysis of GRAS genes in *D. chrysotoxum*.

Gene pairs	*K*a^1^	*K*s^2^	*K*a*/K*s^3^	Time (Mya)^4^	Subfamily
*DchGRAS38–DchGRAS28*	0.216967677	1.134528645	0.19124037	62.33673874	DELLA
*DchGRAS28–DchGRAS33*	0.179621381	1.581916811	0.113546667	86.9185061	DELLA
*DchGRAS28–DchGRAS17*	0.159038717	1.360038863	0.116936891	74.72741005	DELLA
*DchGRAS33–DchGRAS17*	0.132467162	1.230910978	0.107617175	67.63247132	DELLA
*DchGRAS3–DchGRAS30*	0.114861954	0.980996956	0.117086963	53.90093165	SCL3
*DchGRAS11–DchGRAS22*	2.536793813	1.657769547	1.530245152	91.08623885	SCR
*DchGRAS29–DchGRAS46*	0.03801653	0.046311005	0.820896242	2.544560714	SCR
*DchGRAS45–DchGRAS7*	0.001554203	0.012033953	0.12915153	0.661206209	SCL4/7
*DchGRAS41–DchGRAS31*	0.202752185	2.086842634	0.097157391	114.6616832	PAT
*DchGRAS41–DchGRAS32*	0.205621812	2.147275637	0.095759393	117.9821779	PAT
*DchGRAS31–DchGRAS32*	0.004552366	0.017442647	0.260990555	0.958387198	PAT
*DchGRAS21–DchGRAS36*	0.131329889	1.200829768	0.10936595	65.97965758	SHR

^1^Ka, non-synonymous rate; ^2^Ks, synonymous substitution; ^3^Ka/Ks, evolutionary constraint; ^4^Divergence time (T) was calculated by using the formula T= Ks/(2×9.1×10^-9^) ×10^-6^ million years ago (Wang et al., 2021).

### Promoter analysis of *DchGRAS* genes

**Figure 4:**
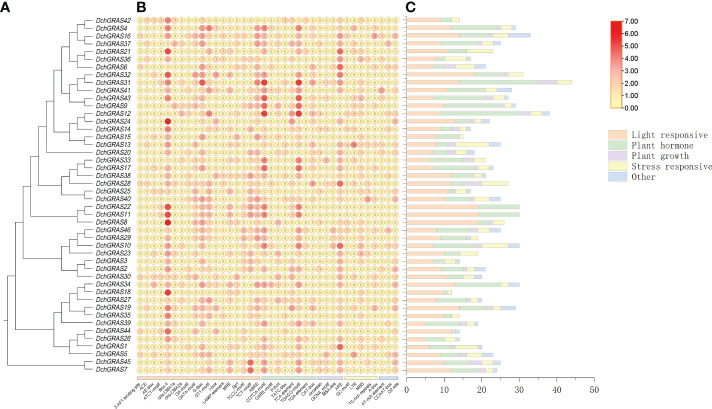
Promoter analysis of the *DchGRAS* gene. **(A)** *D. chrysotoxum* GRAS gene phylogenetic tree. **(B)** The numbers in the circle exhibit the number of *cis*-acting elements in *DchGRAS*. **(C)** The number of light response, plant growth and development, stress response, and phytohormone response elements. The types and quantities of *cis*-acting elements are shown in **Supplementary Table 3**.

In order to investigate the regulatory functions of *DchGRAS* genes, the 2,000 bp promoter regions of *DchGRAS* genes were retrieved for the identification of putative *cis*-elements. A total of 1068 *cis*-acting regulatory elements were obtained, and Box 4, which is related to light reactions, had the most elements, with 137 *cis*-acting regulatory elements. There are eight *cis*-acting regulatory elements related to hormone regulation, with a total of 447 elements, accounting for 41.85%; 17 *cis*-acting regulatory elements related to light response, with a total of 328 elements, accounting for 30.71%; four *cis*-acting regulatory elements related to plant growth and development, with a total of 56 elements, accounting for 5.24%; and five *cis*-acting regulatory elements related to environmental stress, with a total of 145 elements, accounting for 13.58%. *DchGRAS31* contains the largest number of components, a total of 44; *DchGRAS18* contains the least number of components, only 12. The 46 *DchGRAS* genes all contain light-responsive and hormone-regulated elements, *DchGRAS11* and *DchGRAS44* do not contain stress-related *cis*-acting regulatory elements, and 16 genes, including *DchGRAS36*, *DchGRAS3*, and *DchGRAS21*, do not contain growth-related elements (**Figure 4**, **Supplementary Table 3**).

### RT−qPCR analysis

In this study, three groups of *D. chrysotoxum* were treated with high temperature, salt and drought stress respectively, and there was no significant change in plant phenotype before and after treatment (**Supplementary Figure 4**). In order to explore the expression pattern of the *DchGRAS* gene under three abiotic stresses (high temperature, salt, and drought), we performed real-time quantitative PCR (RT−qPCR) on 12 genes from different subfamilies. The results showed that the expression levels of these 12 genes in leaves after high temperature, salt, and drought stress all showed an upwards-regulated trend to varying degrees.

**Figure 5:**
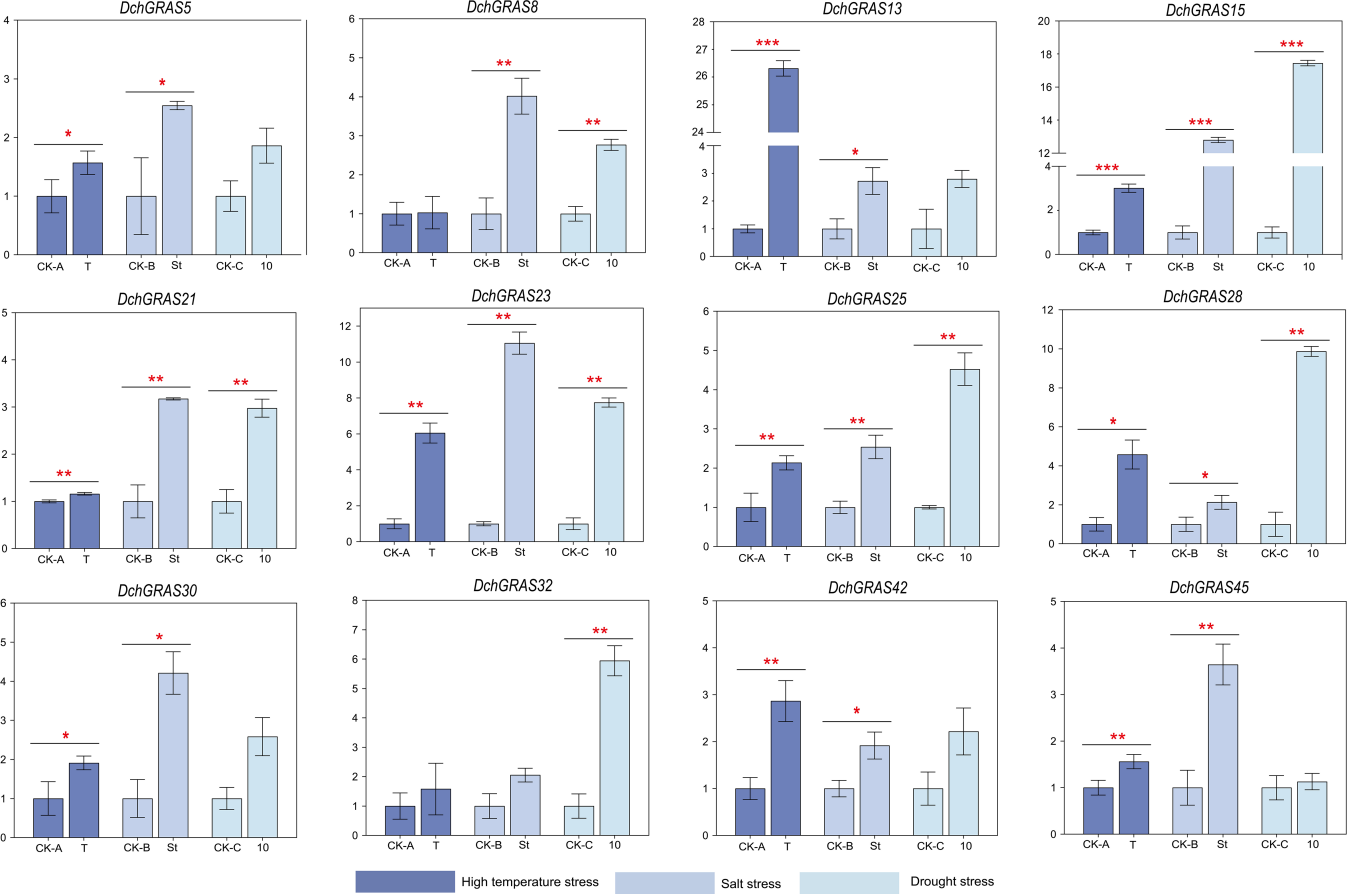
Real-time reverse transcription quantitative PCR (RT−qPCR) validation of 12 *DchGRAS* genes under high temperature, salt, and drought stress. CK-A: Control sample before high-temperature stress. T: Samples treated under high temperature stress of 30°C/38°C for 48 h. CK-B: Control sample before salt stress. St: Samples treated under 0.5 M salt stress for 48 hours. CK-C: Control sample before drought stress. T: Samples after 10 days of drought treatment when substrate humidity dropped below 1.0. Y-axis represents relative expression values. Bars represent the mean values of three technical replicates ± SE. Red asterisks indicate significant upregulation of corresponding genes after abiotic stress treatment (*P < 0.05, **P < 0.01, ***P < 0.001, Student’s t test). Primers and RT-qPCR analysis of 12 *DchGRAS* genes are shown in **Supplementary Table 5**.

After induction at 30°C/38°C for 48 h, the expression levels of *DchGRAS13* and *DchGRAS23* were significantly upregulated by more than five-fold, while those of *DchGRAS8* and *DchGRAS21* showed almost no upregulation. After 48 h of treatment with 0.5 M NaCl solution, the expression levels of *DchGRAS15* and *DchGRAS23* were significantly upregulated; after ten days of drought, the expression levels of *DchGRAS32*, *DchGRAS15*, *DchGRAS23*, and *DchGRAS28* were significantly upregulated, while that of *DchGRAS45* was hardly upregulated (**Figure 5**).

## Discussion

The ecotypes of orchids are diverse, with 70% being epiphytic (Zhang et al., 2016; Zhang et al., 2022). *D. chrysotoxum*, as a perennial epiphytic herb, is widely cultivated and used (Xiao et al., 2014). It has high ornamental, medicinal, and health value. In recent years, the molecular mechanism of flower development regulation of *D. chrysotoxum* and the formation mechanism of medicinal components have been extensively studied (Zhang et al., 2021a). However, there are few studies on the molecular mechanism of *D. chrysotoxum* under adverse conditions. The GRAS gene family has been widely identified and studied in a variety of plants, which proves that it is closely related to plant growth, development, abiotic stress, and hormone regulation (Jaiswal et al., 2022). In this study, 46 *DchGRAS* genes were obtained through comparison and screening based on GRAS-specific domains (**Table 1**). The identification results were similar to those of *Cicer arietinum* (46) (Yadav et al., 2022), *Prunus mume* (47) (Lu et al., 2015), *Dendrobium catenatum* (47) (Zeng et al., 2019); more than those of *A. thaliana* (32) (Tian et al., 2004) and *Pinus* (32) (Abarca et al., 2014); and less than those of *Rosa chinensis* (59) (Kumari et al., 2022), *O. sativa* (63) (Tian et al., 2004), *Manihot esculenta* (77) (Shan et al., 2020), and *Zea mays* (86) (Guo et al., 2017). These differences may be due to gene duplication events or the different frequencies of retained copies after duplication events.

In general, the evolutionary relationship of species is inferred or evaluated through phylogenetic analysis and the construction of an evolutionary tree. The phylogenetic tree constructed in the study shows that the phylogenetic relationships of the GRAS gene family in *D. chrysotoxum* are more closely related to *O. sativa* rather than *A. thaliana*. All 140 GRAS genes of three species are divided into ten subfamilies (**Figure 1**). HAM protein was found to be related to the maintenance of apical or lateral meristem in *Petunia hybrida* plants (Jaiswal et al., 2022). The HAM subfamily has the most members (10/46) in our study. The SCR and SHR subfamilies can form a tissue specific network with BIRD/IDD proteins (Moreno-Risueno et al., 2015), which plays an important role in the regulation of root and leaf radiation morphology. In this study, the members of SCR (6/46) and SHR (7/46) subfamilies may help *D. chrysotoxum* form a divergent root system, which can better adhere to trees or rocks. The DELLA and PAT1 subfamilies are widely studied in many plants, especially the DELLA subfamily. DELLA protein can stimulate GA signal transduction, which is crucial in seed germination, stem and root elongation and flower development (Niu et al., 2016a). DELLA (6/46), PAT1 (5/46), and LISCL (5/46) subfamilies also had more members in this study, and these subfamilies play a crucial part in other plants in coping with abiotic stresses such as drought, salt and photooxidative stress (Khan et al., 2021). GRAS proteins are fully involved in all stages of plant growth and development, and some specific subfamilies are differentiated during the process of contraction or expansion. Such as the Os19 subfamily is a specific subfamily of *O. sativa*, and the phylogenetic tree constructed in this study shows that the Os19 subfamily contains only two members, *DchGRAS20* and *OsGRAS21* (Liu and Widmer, 2014). Most plant GRAS gene families contain members of the Os19 subfamily, but *A. thaliana* (Liu and Widmer, 2014), *Z. mays* (Guo et al., 2017), *Phaseolus vulgaris* (Laskar et al., 2021) and other plants do not contain members of this subfamily. This suggests that *DchGRAS20* may be formed after differentiation of the common ancestor with *A. thaliana*.

**Figure 6:**
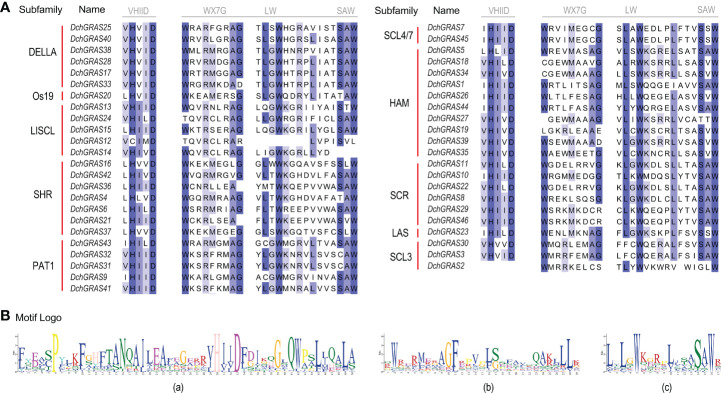
VHIID and SAW motifs in the GRAS protein amino acid sequences. **(A)** *DchGRAS* protein sequence alignment results. **(B)** Sequence logo of motifs. (a) Sequence of the VHIID domain. (b) and (c) Sequence of the SAW domain.

The structure of genes may also affect the phylogenetic relationship. A gene family may be structured differently in different species. The N-terminal intrinsically disordered region (IDR) sequence of GRAS proteins binds to different proteins when expressed, resulting in specific functions (Sun et al., 2012); VHIID is a core structure among five conserved domains at the C-terminus of GRAS proteins (Pysh et al., 1999). The results of the sequence alignment of the *DchGRAS* gene in this study showed that, except for *DchGRAS2*, all 45 members have a VHIID domain, in which aspartic acid (D) is extremely stable, and almost all subfamilies have different degrees of amino acid substitution (**Figure 6**). For example, all members of the SHR subfamily have valine (V) replaced by leucine (L), while all members of the DELLA family with a complete motif backbone have isoleucine (I) replaced by valine (V), and the PAT1 subfamily also has a few amino acid substitutions. The PAT1 and DELLA subfamilies play an important role in regulating plant photosensitive signal transduction, GA signal transduction, and stress resistance (Torres-Galea et al., 2006; Wang et al., 2014; Zhang et al., 2021c). In this study, they have a complete motif skeleton structure without deletion. Therefore, the large number of amino acid substitutions in these two relatively stable subgroups may be the embodiment of the slow evolution of the *D. chrysotoxum* GRAS gene family to adapt to environmental changes. In addition, SAW is also a relatively conserved domain at the C-terminus of the GRAS protein. It is composed of three consecutive parts: WX7G, LW and SAW (Hirsch et al., 2009). In this study, most *DchGRAS* genes had this domain, and there was less substitution than seen in the prior study. Although the function of the SAW domain is unclear, these conserved elements play a vital role in maintaining the integrity of the domain and playing a role in stabilizing the protein (Sun et al., 2011).

**Figure 7:**
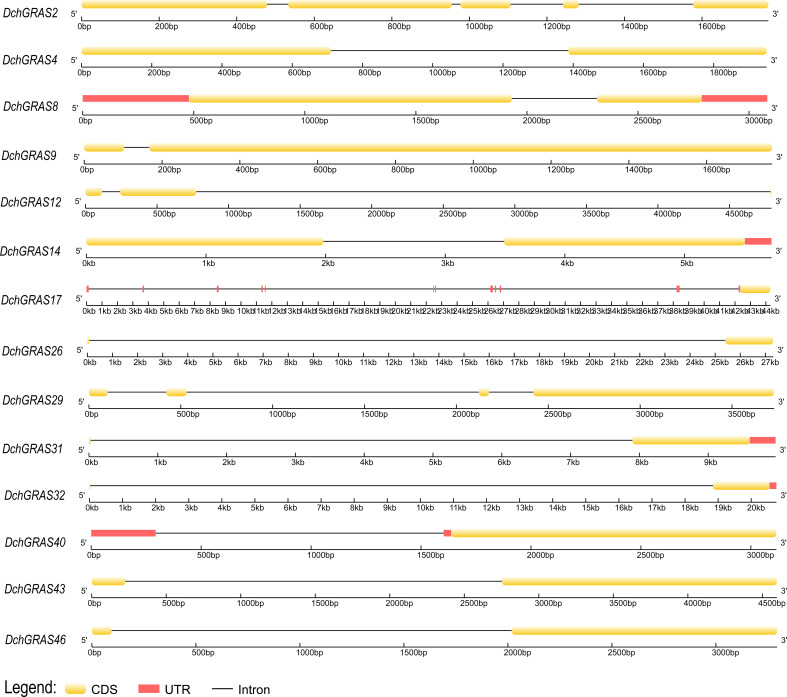
The structures of 14 *DchGRAS* genes of *D. chrysotoxum* containing introns. The intron-exon structure was drawn using a platform on the GSDS website.

During evolution, introns are considered to be one of the important reasons for the formation of new genes. 69.56% of *DchGRAS* genes in this study lacked introns (**Figure 2C**) and more than 70% of GRAS genes in other plants such as *Z. mays* (Guo et al., 2017), *Glycine max* (Wang et al., 2020) *and Melilotus albus* (Wang et al., 2022) lacked introns. This phenomenon is parallel to the early reports on the conserved characteristics of GRAS genes. Some studies have found that intron-free genes also belong to large families that regulate physiological and biochemical processes and participate in growth and development processes, such as signal transduction, protein synthesis and conversion, and metabolism (Jain et al., 2008). Although intron-free genes have no advantage in species evolution or recombination, they often respond quickly to stress (Sang et al., 2016). Therefore, many *DchGRAS* members may be able to respond quickly to environmental changes, and these intron-free genes are the main driving force of plant tissue-specific evolution. Orchids have the characteristics of high heterozygosity, high repetition, and long introns (Cai et al., 2015; Lu et al., 2019). Three genes in this study (*DchGRAS17*, *DchGRAS26*, and *DchGRAS32*) have introns over 10,000 bp in length, probably because they contain a large number of transposable elements (**Figure 7**). Genes containing introns promote the evolution of plant species by participating in translation and energy metabolism, and these genes with long introns are important for the abundance of orchid species (Jain et al., 2008).

*Cis*-acting elements are involved in the dynamic network of gene regulation. This study identified many anti-stress responsive *cis*-acting elements in the upstream 2000 bp promoter region of 46 *DchGRAS* genes (**Figure 4**). At the same time, some hormone-responsive elements, such as ABRE (ABA response), P-box (ABA and ethylene response), and TATC-box (GA response) (Cao et al., 2021), are also widely present in *DchGRAS* genes. Plants need to adapt to changes in the external environment through gene regulation. In recent years, an increasing number of studies have shown that GA anabolism and signal transduction pathways are involved in abiotic stress in plants. When GA content decreases, the corresponding *cis*-acting elements begin to respond, and DELLA proteins bind to transcription factors, inhibiting downstream gene expression and thereby inhibiting plant growth (Achard et al., 2008; Shan et al., 2020). This may be a key pathway for GRAS proteins to regulate plant growth and development under stress conditions.

We selected 12 *DchGRAS* genes from different subfamilies for RT−qPCR validation analysis. The results showed that after high temperature, drought and salt treatment, the expression levels of 12 genes were significantly different under different stresses, and there were also differences in expression among members of the same subfamily (**Figure 5**). For example, *DchGRAS13* and *DchGRAS15* all belong to the LISCL subfamily, but the RT−qPCR results showed that *DchGRAS13* is significantly induced under high temperature stress and increases up to 25-fold, while *DchGRAS15* does not change significantly under high temperature stress but is upregulated ten-fold under salt stress and drought stress. This phenomenon also exists in GRAS genes from different subgroups of other plants after stress treatment, such as *Solanum lycopersicum* (Niu et al., 2016b), *Populus* (Liu and Widmer, 2014), and *Camellia sinensis* (Wang et al., 2018). This suggests that the genes of the same subfamily may have different roles in the signalling pathways of abiotic stress responses. A large number of studies have shown that the DELLA and PAT1 subfamily are fully involved in the growth and development processes of multispecies abiotic stress, light signal transduction, seed germination, and other processes and control plant height by influencing the growth of inflorescence stems (Khan et al., 2021; Jaiswal et al., 2022). In this study, members of the DELLA subfamily (*DchGRAS25* and *DchGRAS28*) were induced under all three stress conditions, and their expression was significantly upregulated under drought stress. The expression of *DchGRAS32* of the PAT1 subfamily was upregulated under all three stress conditions, but especially under drought stress, under which its expression was upregulated by more than five-fold. LAS subfamily is closely related to the development of plant lateral buds (Schumacher et al., 1999). *OsMOC1* in rice belongs to LAS subfamily, and its overexpression can increase the number of rice tillers, thereby reducing plant height (Li et al., 2003). LAS homologous genes with similar functions have been found in *S. lycopersicum*, *Nicotiana tabacum*, *Cucumis sativus*, *Zoysia tenuifolia* and other crops (Chang et al., 2013). In this study, the expression of *DchGRAS23*, a member of the LAS subfamily, was also significantly upregulated under stress conditions, which indicated that LAS subfamily members likely function in stress tolerance and provided a reference for exploring more functions of LAS subfamily members. In addition, studies have shown that *AtGRAS14* of the LISCL subfamily is also a key gene regulating plant development and stress and is related to the occurrence of anther microspores in *Lilium longiflorum* (Morohashi et al., 2003). *DchGRAS15* and *AthGRAS14* are homologous genes, which are also up-regulated under abiotic stress. To sum up, this study speculates that the normal growth and development of *D. chrysotoxum* and the maintenance of the swollen pseudobulb under abiotic stress may be regulated by members of the GRAS gene family.

## Conclusion

In this study, 46 *DchGRAS* genes were identified from the *D. chrysotoxum* genome and divided into ten subfamilies according to their phylogenetic relationships, and members of the same subfamily had similar gene structures and conserved domains. The results of real-time reverse transcription quantitative PCR (RT−qPCR) experiments showed significant differences in the expression levels of 12 genes under abiotic stress, among which LISCL, LAS, and DELLA subfamily members were the most sensitive to stress. This result will provide valuable information for further studying of *D. chrysotoxum* and other orchids under abiotic stress.

## Data availability statement

The datasets presented in this study can be found in online repositories. The names of the repository/repositories and accession number(s) can be found in the article/**Supplementary Material**.

## Author contributions

SL, Z-JL, and DZ contributed to conceptualization and validation. XZ, D-KL, and CZ prepared the original draft. SK, YL, Q-QW, and QZ analysed the data. All authors contributed to the article and approved the submitted version.

## Funding

This work was supported by the Forestry Peak Discipline Construction Project of Fujian Agriculture and Forestry University (72202200205), and the National Key Research and Development Program of China (2019YFD1000400).

## Conflict of interest

The authors declare that the research was conducted in the absence of any commercial or financial relationships that could be construed as a potential conflict of interest.

## Publisher’s note

All claims expressed in this article are solely those of the authors and do not necessarily represent those of their affiliated organizations, or those of the publisher, the editors and the reviewers. Any product that may be evaluated in this article, or claim that may be made by its manufacturer, is not guaranteed or endorsed by the publisher.

## Supplementary material

Supplementary Table 1 GRAS protein sequences used in the phylogenetic tree;

Supplementary Figure 2 Sequence and logo of Motif1–10;

Supplementary Table 3 Promoter and *cis*-acting elements analysis of *DchGRAS* genes;

Supplementary Figure 4 Plant images of *D. chrysotoxum* before and after different abiotic stresses;

Supplementary Table 5 Primers and RT-qPCR analysis of 12 *DchGRAS* genes.
