# Supplementary material for: Genome-wide identification and expression analysis of the GRAS gene family in Dendrobium chrysotoxum
Source: Front Plant Sci. 2022 Nov 28;13:1058287. doi: 10.3389/fpls.2022.1058287 (PMC9742484; doi:10.3389/fpls.2022.1058287)
Supplement: Supplementary Table 1 — GRAS protein sequences used in the phylogenetic tree; [file Table_1.pdf]

>DchGRAS1
MLFHALEDEEDFFSNRTRKSSSKEGGEPRSVLDNQRSPSSPTSTSTPTSSDTIGVAAVFDVP
AHKCQQVSASSNGEWASELKPITMSLGSVEGWDSMLLEPPPVETAELSSVHSLSRWFMVD
SNSGAMLEGSGIGLEILDPGFCFQHIGGMGGDGSIIPMVPDPPSNLLPNSGFTNKSLKAPAF
ANSNFIINQNPHENYMPQTYLGACAIPIQYPNEAAWLQHAVIDLLFEAAELVESGNTINAQ
RILARLNHQVPSPYGKPYIRSAFYFKEALLTISNRGGEQCGFRLASPLDILLKLSAYKAFAEI
SPVLKFISFTSTQLILEELAGANIIHIIDFDVGVGSQWPALIQELSERRSAVTGVPLQLRITAFF
SISSHHQLELHLIEENLNFFAANLNVPFELNFLPIESFDPAAIISSSSHSGEAIAVNFAFGFCSH
HISTHALLLLIKQLSPKILISANLGYDRGDLSFSHHVLHSFQSSTFLLDSIEASGVSPEITDRI
ERYLLRPMIESSMFGRYRTREKFLPWRTLITSAGFVPMQFSNFTKTMADCLLKKNQVRGF
EVEKRQAAMMLSWQQGEIAVVSAWSC
>DchGRAS2
MAGTADELSSSSATSSHLLSLMSMSPTTITSPYAPFFRDLNADERGLFLIHLLLNCATHVAA
GNIAQANTFLEHISLLSSPDGDTMQRIASYFTEALAARLLRSWPGLYRALNPILPDTAAASN
HGRRLFLDLFPFLRLSFLISNQAIFRSDGRGESRPLSGRPEGPPHLRIITAVGEDRDFLTQTAL
VLTEEAEKLDIPFQFHAVVSQLDGLNIESLRVKTGEALAITSVLKLHTLLATDEATATPRQLF
SPIDKIESFLASLWGLSPKLMVVTEQESNHNGTNFNERFMEALNFYAALTTAVCGAVEVEK
LVFREEIKNIIACEGLERKERHEKLHKWMRRKELCSLFQLGDLEGECCGGIIARPSLVDCE
KCRVMPAPNASGGQFGCVTIPNTLYWVKWRVWIGLWKHLTSARNNHS
>DchGRAS3
MQRIASYFTEALAARLLRSWPGLYRALNPILPDTAAASNHGRRLFLDLFPFLRLSFLISNQA
ILEAMEGEKVVHVIDLGGSDPSHWLAFLQAVSGRPEGPPHLRITAVGEDRDFLTQTALVLT
EEAEKLDIPFQFHAVVSQLDGLNIESLRVKTGEALAITSVLKLHTLLATDEATATPRQLFSPI
DKIESFLASLWGLSPKLMVVTEQESNHNGTNFNERFMEALNFYAALFDCLELAVPRQSAE
RLKVEKLVFREEIKNIIACEGLERKERHEKLHKWMRRFEMAGFGRVGLSHYGLLQARRM
LQGFGCEGYKVKEEKGCFLFCWQERALFSISAWRFRRYD
>DchGRAS4
MQFTENPPPPPLYPTRPWPDFPNSAKSLGSFNNAACMEQLLVHCAHAIEANDATLAQQLL
WVLHNIAPPDGDSNQRLTSAFLRALLLRASRSGSSISGPATPHHPQLPLRLSAISLAGFIDLT
PWHRFGFSAANSAIADAADGFPVLHLVDLTTTHCMQIPTLIDVLASRPEGPPFLRLTLPSTP
ASTSPPPTLDISLDELGSRLVNFARSRNVGMDFRVVPSTPADGFHSLLEYLGANCNNAGEA
LVINCHMMPHRIPDESAGGILSPRTVFAKSLRSLDPSLVVLVDEEADFTSSDVVTRLRAAFN
YMWIPFDAVETFLPAGSEQRRKYEAAVCWKIENVIAQEGVQRVERLEPRGRWGQRMRAA
GFVGIGFGEEAVMEVKSMLDEHSAGWGMKRDDDDLVLTWKGHDVAFATAWIPSSATS
>DchGRAS5
MKITKSSTGGDSHAALHHLPKPIDSSPLYEPTSVLDPHITSSPSAAAAAAPVTGGVCPAPSL
PWDPASIPLALNSSASAASDDWSDPLSFLLADMDDPCSFFPDLSHPTPPPPEAESSHLDLLL
RAAHAVEIGDLNSSLSILARLNFLLPCSTGSPLHRAAFHFKDALLSLLPIPDRPIPEPPLSAIE
LVLRIAAHKAFSDLSPIPQFTSFTANQTLLESVDAAPSLHLIDFDLGLGGQWSSFAHDLAAR
SRAARLPPPPLRITAVVSEETAETALAADNLRDFARGIGLRLTVEFVRLSALAGVRLAPGEA
VAVVLTPTIFRLIGVSVLRFVRRVAARVVLVVDGEGYCGSVGSFRRRFAGGLEYYAAAME
VVDAWAAAAGAGEEAVRRIERALFRPRIFAAVAAAAAAVGDWREVMASAGMVVAELSEF
TESQAEWLLRRAPVEGFHVARREGALVLSWKGRELSATSAWRCR
>DchGRAS6

MDEDFSSSSSSSKHFQHPDQPPAPPPPPDLPLPDLFSDPNRRWASDLLLDCARAVASRDSSR
VQQLMWMLNELASPYGDFDQKLASYFLQALFARLTSSGPRTLRTLSSASDRTASFDSTRRT
ALRFQELSPWSSFGHVAANGAILESFLLHSPSSTPSRLHILDLSTTFCTQWPTLLEALATRSA
ADEDTPHLSITTVVPSTSPTARRVMREIAARMEKFARLMAVPFRFSVVHHPDADLSALDLD
TLISSDSSSAATALAVNCVNSLHGVSPSARHRDVLLGKIRRLRPRIVTVVEEEAELDFDEDD
EEGFLKGFREGLRFFSAYFDSLEESFPKTSNERLALERAAGRAMVDLVACPASQSAERRDT
AAGWSRRMRIAGFSPLAFSDDLADDLRALLRRYKEGWSMRAAVAPDEADESTSSGGGGG
GGAGCGMFLTWREEPVVWASAWKV
>DchGRAS7
MAYMCADSGNLMAIAQQVIKQKQQQQQRHQHQQQNQEPVIPAIDVASSDGDPFAAVPPQ
WGNQHQQTTPIRDHFPFALPEHTFHDPFAAGVFGLPPPTHNDHAPFRIPEFDSDEWMESLI
GESPTESSEIMADVWQRTDLPPIFPDAFASCSTAISLPSQASTSDLDRVVFPDSCKIVAPHSLS
FDPPPPPQPKKDAVDSKSPPLLFKSLLDCARIIDTEPDQAAKTIDHIRDLASEHGDPTERVAF
YFAEALNSRLTGCSDPDFREEEIALCYRAFNDACPYSMFVHLTANQAILEATESAEIIHIIDF
GISQGIQWAALLQALATRPTGKPTRIRISGIPAASLGGSPATSLAATGNRLLDFARLLDLDFE
FEPVLHPVQDLTAASFRVEPGEAVAVNFTLQLYTLLGDSDEAVNRVIRLAKSVRPSVVTLGE
YECSLNRIGFVERFRNALEYFAAVFDSVESSRGRDSDERARIERVILGPRILYTVGPEDGRQ
RRMRMEAKEKWRVIMEGCGLEMVPLSNYAVSQAKLLLWNYDYSPKYKLLDSSTGILSLA
WEDLPLFTVSSWR
>DchGRAS8
MASTSCLLLPHPHDPSNASPSSINSQEDPLIFLQETSFTPLPAVKMARKRPASELDLQADPPR
RPPPPFSSDQLLLSETPQSLALSSLPPLPPSAPAPSTLCSFSGLPLFTSEPNRNATAPQNSTFD
AEDTSSSAWIDGIIRDLINSAAANVSIPQIIHNVREVIFPCNPSLAAVLEFRLRSLSSDAQTPA
NKRREPSQCNLVPSWEEQQNDNNATTYPPNAAVSASSEEAAAAPPPPPPPPPTALASATRK
EELRQQKRDEDGLHLLTLLLQCAEAVAADNLEEANRLLLEISELSTPFGTSAQRVAAYFSE
AMSARLISSCLGIYAPLPPIAVHRQRLASAFQVFNGISPFVKFSHFTANQAIQEAFEREECVH
IIDLDIMQGLQWPGLFHILASRPGGPPRVRLTGLGSSLEALQATGKRLSDFAETLGLPFEFCP
VADKVGNLDPDRLGVSRREAVAVHWLHHSLYDVTGSDTNTLWLLQRLSPKVVTMVEQD
LSQAGSFLARFVEAIHYYSALFDSLGASYGEESQERHIVEQQLLSREIRNVLSVGGPARSGE
VKFGNWREKLSQSGFRGVSLAGNAHAQATLLLGMFPSDGYTLMEENGTLKLGWKDLCL
LTASAWKPIQTPVTTCVGYGGVAR
>DchGRAS9
MEKGCAFISGLSFLWFGAEEKAAVVATASDLQTKGPGRDDPRGALRFGVESTGGLFKRSL
MTEQEAQNAVFVRSVKQKNHLELPISALSPTSSIQSSLSSASIPLRGQGNLKFTSWSLPAPTS
VEPEKKSTITNRLLQLERQLLDDEDDELSVSDGSAVTNSEWNETMQKLIFPQKPLSNSPTS
SSSSSSSSSITSSRQMLLDTATAISAGNLDTAAANLALLKGAANKLGNPEQRLTAMMVTAL
ALRINPRGPATIAVTSDIFTPEHLTSSQMLYEAAPCFKLALMTANLAIIEATKDHPKIHIIDFD
VGQGLQLASLLHSLAERHRHISVKITAFCDPTLPFNSLAGNLNLVGDRLAKLAEGLGIGLR
FSTVNRRTTELDRAALDCGTEEALVVNFAFLLSRIADESVSPANPRDELLRRVKALGPKVV
TLVEQEMNGNTAPFATRFAEACAHYGALLESMEAIIVGRESNERARVEACLARKACNSVA
REGADRVERCEVYGKWKARLGMAGFKPVQLGPGVVETVKRRLASLGNNPGFMVKEED
GRVACGWMGRVINVASAWH
>DchGRAS10
MGTQRLDLPCSFTRKEAAMVSLSLERPAESRGSCSFRPRPVTISSSAIIAQTASWESRREIGR

ELWNRKRSFKKFHQCGLFEDPDIDRAKKKRTNSSGEGDYNDEKKPSFLAGQFQAGTSGCP
NIKSGSSTSGCPNIKSGSSSSSDTHGLSPKLSEDSSHNEEENPSGEVSGFESAVKKTKTEHEG
LELLSFLIECAQAISSSNHTAANFYLARLGDMSSPTGTSIQRLVAYFTEALAFRAAKKWQHI
FSIAPPRELTEMNDHDEAMALRILNNVTPIIRFIHFTLNERLLTAFEGKDRIHIIDFDIKQGLQ
WPSLLHSLASMPNPPTHVRVTGIGESKQELQDTGTRLAGLAQALKLPFEFHSVVDRIEDV
RLWMLHVKENECVAVNCVLQLHRALYATNHIVLMAMLGLIRSTNPSIVLMAETEAEHNEL
RWEKRFANALKYYSAVFDLLDYSFTGDSASRIKIEEMFARKIRNLVAYEGVERIERHERFD
MWRGMMEDGGFRCVGFNEREIIQSKLLLKMYNCENYSVDKEGGDGFGLTLKWMDQSL
YTVSAWAPMEIVGTSSALQPG
>DchGRAS11
MKPSKRSSPSSNSDELPAGKKPHYSPLAEAGATSSSTSTSPSPEQESRILRLLTLLLHCAESIA
LDNLSYALTLLPEISSLSSPFGSSPERVAAYFAEALHARILSSFLGTYSPLSLKPLTLTLTRRIS
RSFQFLNSISPLLKFSHFTSNQAIFQALASHDRVHILDFDIMHGLQWPGLFHMLSSRARPIQ
SIRITGIGPAAEILDSTGRSLADFAAGFRIPFDFTPIEGKIGDLTDLTPFRPSDPSEAIVVHWTQ
HCLYDVTGSDLAAAQILKSIRPRIVTVVEQDLGQGGDFLCRFVEALHYYSALFDAIGDGG
GGGEERWEVERGVLGGEIRNIVAIGGPKRTGEVRVERWGDELRRVGFRPVSLAGSPASQA
SLLLGMMPWKGYTLVEEGGRLKLGWKDLSLLTASAWRPAAGAGETEGGEGEEWDTSDS
LVI
>DchGRAS12
MIYHFLNQTILKVIGNTSRVCIMDFSIYSDFHWPPFMKIFHIPFVYQVLASKLDEVKVEDLH
LKEEDVLIINCLFRINSFGNETMNVDFTRDKFLNNLRKLNSALFVNVTTMEPLELHFFVMR
FREVLYHLSRLFNRLEVTALREYEQRMLVERDLYGNSMIDIISCEGAGRLESPKMYKQTQV
RCLRARFEQLPFSSEIAIIHVKLVPISVLVKPF
>DchGRAS13
MDRRTRELSGVDGLSCEDFFSYQNPGGFKLNYPQPSVMDSSSLHGSKFDASQLFPVQNNL
HGHKYSVADPNLNQSNHANYEFSGSGSVSDGNPPSTSSRSHEVDYPEDSEIFSDIVLSYISR
ILMEEDVDEKIYPFNQEKSALQAAEKPFYDILGQQYPPPSPNKSFLDEDSRLESPEETSSSY
GVFSGESSSSGVVDPSWIQDSSVRNQLTFSSSSVGSVDPLPVPSLLIESQPAWQFKRGMEEA
QKFLPSENKLAIDVNVKNLALNYNLKEKGFTFDTEHKVEKDQLLPIVGSRGRKKPLSEDL
DLQEGRSIKQSAKSSDDAVRSEMFDNVLLCQGVKTYGANKMNGYHMAMQNSANKSSQ
NGRKGRSKKQQKKDVVDLRTLLIHCAQAVAADDRRSANELLKQIRQYSSPFGDGTQRLA
HCLADGLEARLAGTGSQIYHSLVAKRTTATDILKAYKLYLSACPFKKISHFFSNQTILNVSE
KASRVHIIDFGIYFGFQWPCLIQRLSLRPGGPPKLRITGIDVPQPGFRPTERIEETGKRLADY
AESFNVPFQYNAIASKWETIRVEDLKIDEDEVLIVNCLYRFRNLVDETVVVDSPRNKVLSTI
RKANPDVFIHGIVNGSYSAPFFVTRFREALFHFSSLFDMLETTVPREDAQRMLIERDLFGR
EALNVISCEGSERVERPETYKQWQVRNLRAGFTQLQLNPEIMKRAKDRVRSCYHRDFVID
QDSRWLLQGWKGRIIYAISTWKPSEK
>DchGRAS14
MSKDSITAESLKSSDFLGQPLNDRISFFESPVSNPRSNEGESQERSDIFSDELLCSINQMLME
EDADDKYVMLHSHPDLEATEKPFYELIGKKYPPSPDRPPLYSSPSPESFGDNRGDNKVISGI
DFFQANSEIAEYQNWDIYPFHQSQEDQFTDHFFKNLPASEYKRGVEEAKKFLPSEHKLVVS
VGEADLSVPQELIERAGRKQNRHSDASGYDEARRTKQSAFSSEESMISLEALDETLLCYG
DKFPKVVKTLRENIYKEASKYPKNTNPKEVGSSLKYPRNKQTSEEEEADINTLLLQCAQT
VAIGDNPRAYALLKQIRQHSSLHGDAFQRVAHYFADGLEARLSGTGTEVYRSLVYKRRTIK

DVLNAYQFYLASCPFKKVSYHFANQTILDTIQNATRVHIVDFGIYFGFQWPVLLKLLSLRP
GRPPKLRITGIDIPQPGFRPTEIIDETGRRLADYAEQFGVPFEYHGIAAKFDDINVEELYIKEE
ETLVVNCLFRLHSPADETVVENCPRDKVLNTIRKMSPAVFVNVVLNGTYGSPFYMSRVRE
ALYHFSALFDMLDSTTPRESERRLLVERNLYGPLLINAISCEGMEREMPETYKQWQVRCL
RAGFEMVPNSKDFVKKVKDGVRSIYHKDFNMDEDGRWLLIGWKGRLLYD
>DchGRAS15
MQTNLHLQAFLSKEIPTSDASSNPRPSSGQDSHEDAEIFSDIVLTYMNQILMEEDSDEKLD
MLQEHPALLAAEKSFYDILADTQFSSSSSNRPTFPSNHHSSDNDSLNPQGEPPAKSTNSRRT
LQGVDNLEAEEGRSLKQSALGFDTEVPPEMLDALLFKECKGNRQVETCYKTSHGGTKSG
TKKKLEKKESVVLDSLLIQCAQAISENDLPHAHKLLNQIRQHSTPFGDSNQRLAHCFANGL
EARLTGGGGGGSVSYYFQSPPPATVSDMLKAHQIYLAAAPFKKISDFFAHQNILNVSGKSR
RLHIIDFGIYHGFMWPCFLQRLSTLPGGAPMLRITGIDFPQPGFRPAERVEETGRRLSEYAR
RFDIPFQYHPIACKWEALQLEDFGIEEDEVLVVNCMNRLQNLADESVSPDNPRDQVLNTIR
KLKPDVFVLGIVNGTYNTPFFLRRFKEAMFHFSALLDMLETNVGREDEHRRLLERHFFER
EAFNVISCEGLERVERPETYKQWKTRSERAGLVQVPLDADIVRKSRWKLKRCYHKDFMV
VEDGKWLLQGWKGRILYGLSAWRTRDRALKESS
>DchGRAS16
MDWLEDSIFPLPFLDDPYTLTEDIARYDWWPQTQNQIQSPTIAAVVPPATVNPKAITPKSEL
SKKRKFQSNPPAAQRRRTGNEGHNEKKTHGGVTGGSREARWAEQLLNPLAAAIESGNISR
AQHLLYVLQELASPTGDPNHRLAFYGLQALTHCLSGGYAKAEPKGFRSALIKFHEVSPWF
ALPNCLANAAIVSASAGAKRLHVVDAGVSHGVQWPTMLEALTRRPGGPPSMVKLTVVD
AVSNGPFGLAPEGYDFGPPLMRYAKSIEVDLIVERGEMVERRRVGDEERVVVCAQFRAGR
AGLAFLRAVREIEPDLVVLSEGEEEGEGEGLGFGRRAEVMWRFLESTSAAFKGKECEERR
VMEGEAARVLEERREGGRERWKEKMEGLGFREQVFGEEVVDAGRALLRKYDGNWEMT
CGSEGRVGLWWKGQAVSFSSLWKPARHKTTATAAVAAAGCGNGVRHY
>DchGRAS17
MKREHLETVGGRGIGGYAPAAPGMAGTGKGKMMGLDEEADGGLDELLAAFGYNVRSS
DMADVAEKLEQLEMAMGGSAAHDDALLIHLASDTVHYNPSDLSNWLETMLSELSAPPPQ
LPQAIHPNSSSYNLPQAPASAHSSTAFFDPADALTTTESSTVTSVDFPAQPPPVVFPDFAARQ
SGSGGNRVVYGSEPQIDSGTRDRKRMKTSSSSSSSRGGSGGGVIVQSSPTIGSLLAPASGTE
VSSALPVVMVDTQEVGIRLVHALMACAEAVNQENFKVADVLVRQITVLASSQGGAMRKV
AGYFAEALARKIYRLHPQQDCSLDSAFSDILQMHFYESCPYLKFAHFTANQAILEAFAGCR
RVHVIDFSMKQGMQWPALMQALALRPGGPPSFRLTGIGPPQPDNTDALQQVGWKLAQLA
DTIHVDFEYRGFVANSLADLEPFMLETLSPASGGSAEPEVVAINSVFELHRLLGRPGALEK
VLATVRAVRPRIVTVVEQEANHNAGPFMERFTEALHYYSTMFDSLEGGAGAAGEAQQDQ
LMSEVYLGRQICNVVACEGSERTERHETLTQWRTRMGGAGFEPVHIGSNAFKQASMLLA
LFAGGDGYRVEEKEGCLTLGWHTRPLIATSAWRVAGATDSTSASAAADAH
>DchGRAS18
MKNIPELIIPASSSTTTTTADEDNNWDDWSSDINWCQVSGDFRSDLPSFILPTAISSYPAGEA
DHLAGAHAIHDWSYPSTTTNSHATTPSTEGPTRNELYNFNEPTSVSPRLIHLLIAAAEAISG
DQKSPHLARVILVRLREIIPTDHAAASGIERLASHFTEALLALLDNPHQPHQPATPTGEVLIA
FKLLHDMSPCVSFGHLTANQAILEAIAGERRVHILDYDIGEGVQWASLIQALISKSSPAAPH
LRITALTNGIKGSASGAQEVGRRLSAFAASVGQPFSFRICRLDHTKRFTPAAIKVVKGEALV
VNCVLQPAQLSSAASLTSFLTGAVALGPRILTIIEEESTPFVARAERGFVGEFMDEVERYMAI

WESLETGFPMQGRVREMVERVILRPRIAAAVARAYGRQDGGEVAAERCGEWMAAVGFER
LELSSFNVCQAQLLLGLFNDGFCIDKDSPNKLALRWKSRRLLSASVWGLPPPPSPSASLPF
>DchGRAS19
MNEMMEREIFGFSSTNSIPINADLVWRDWSPGINWDDFTGADNFHGQIESMMPAAVDLLD
WYTCVSPTSTNCPGSPAENLIPSSDDTATVRLVHLLKAAAESLTGDLKNPFLTEAILARLKE
LLARSASTSMERIAAHFTNALHSLLDAAGDIEEPLHQPCEFLGAFLLMQDMSPCIKFGHFT
ANQAILESVAGERRVHIVDFDIAEGVQWASLMQALVSINNGSLPPHLKITAVERYRRSVQE
TGRRLAAFAASVGLPFSFGQCRADYDGQFRPAGIKVVRGEALVFNCILQPGSAAGSSSFIS
GAAALGARVVTIVDEESEGVGFEGRFMEEMMRYSAIWDALDAGFEKQGREREMVERVF
LGPRIARAVERAYKGIEAWENLGKRLEAAEFRRMGLSFFNICQARLLLCLFSEGYQIEEEG
TNKLVLCWKSCRLTSASVWSAPPLLSPEEESYVSS
>DchGRAS20
MKGEVADDLLNLSLSLGQTKKRKRTDHHSYSPNKNLTIAAVLHARNRMLHPTTPIFLDHN
IDGLHLIRLLLLSASAADLHDFSTAVESLLHLYPQLSLSGDPIQRVAAYFSDAILATLLAPSSP
LYSSISPSPTPHEEFSAFTSLYLASPYLQFAHFTANQAIIDAFEAEEHWNGGSLHVIDFDVSY
GFQWPSLIQSLADKAKKDKPFKLRITGFGRSEAELKETEARLSVFSKGCRNLYFEFEGKNS
EINRLKDLNLWENSTVVVNSVFNLHMLKSSTLQLIQSLNPSLLILVEKEGGRTETRSKSFLS
RFMESLQYFAAMFDSLDDCLPAGSKERLRIEKNYLGREIRNAVDMKQEEDEKEVLMKYG
TFATWKEAMERSGFEGVMMSSRSVSQAKLLLKIKSHCSPMEEHGGGAGFRISERDDGRAI
SLGWQDRYLITATAWRCNSQ
>DchGRAS21
MQNSNFFLQQGDPSVANKWAANLLKECAKAISDKDSAKIQHILWMLNELASPYGDCEQK
IASYFLQALFCKVTESGEQSYRTLVSAAEKSHSFESARKVILKFQEVSPWTTFGHVAANSAI
LEALEGEPKLHIIDISNTYCTQWPTLLESLATRSDETPHLKLSVVVIGGGAGGSVMKEIGQR
MEKFARLMGVPFEFNVINRNSSLSELREKEFGVKEDEAVAVNCIGAFRKVGVERREEFVR
MISSLRPRVVTVVEEEVDFSSNRDDFFKCFEECLRFYSMFFEMLEESFVPTSNERLMLERE
CSRSILGLLACGSDGECAERREKGSQWCKRLSEAFVAASFSEDAIDDVKALLKRYQLGWS
LLPASGDSSGLFLTWKEEPVVWASVWKPV
>DchGRAS22
MKPSKRSSPSSNSDELPAGKKPHYSPLAEAGATSSSTSTSPSPEQESRILRLLTLLLHCAESIA
LDNLSYALTLLPEISSLSSPFGSSPERVAAYFAEALHARILSSFLGTYSPLSLKPLTLTLTRRIS
RSFQFLNSISPLLKFSHFTSNQAIFQALASHDRVHILDFDIMHGLQWPGLFHMLSSRARPIQ
SIRITGIGPAAEILDSTGRSLADFAAGFRIPFDFTPIEGKIGDLTDLTPFRPSDPSEAIVVHWTQ
HCLYDVTGSDLAAAQILKSIRPRIVTVVEQDLGQGGDFLCRFVEALHYYSALFDAIGDGG
GGGEERWEVERGVLGGEIRNIVAIGGPKRTGEVRVERWGDELRRVGFRPVSLAGSPASQA
SLLLGMMPWKGYTLVEEGGRLKLGWKDLSLLTASAWRPAAGAGETEGGEGEEWDTSDS
LVI
>DchGRAS23
MLTISSPPLDQMLTSFTSHEEDHNQLQQQQHHHLLLHPRPEPASNRSVQSHRLLLHCAEL
IHHADFAAARHAISILTTTSSATGDAADRLTHHFTSALSLRLHLLPPSPHDPNFILSFNQLTPF
LRFSHLTANQAILEAVDGHRSIHILDFDTGHGLQWPPLLQAIADRSHFPNPPTIHITGTGTDP
SLLSQTSRRLQSFADSLGLCFHFHPLCISPTTSFSFQTNGGEILAVNCTMFLHKLIKDDGLDE
LKQFLLAIKGMNPAVVTVAEKEAKHSSPLFLQRFAEALSYYSAVFESLEATLPPSSRERMV
VEKMWIGREIEDIVAGEGEGRKERHERFERWENLMKNAGFLIKPLSYFALAQARLLLRLH

YPSEGYRVEMMKGSMFLGWKSKPLFSVSSWF
>DchGRAS24
MNNPMSNEANDDFKYKDFFSVDEFLAQEESLATNPNFGEPQLFNEQNYFDELPNSSYLSH
EADSQGNSDDFSDLVLDYIGQMLMEEDMDDKFDMLESNPALEATEKPFYEIIGEKYPPSPD
RPPIYSSPSSESSEGHRSHNFHSSGITGVNGIADNNQDPAYAESTEFPHSQISSYQTSISSDYS
VASFLEGDPDDFSDLLFKNLPASQFEKGVEEAMKFLPSEVKLVVDAGDIGFESPHELKEDL
ASIEGRAEDASGRRQKGRHDINFDLEEQRSSKQSAISYEEPTVSSEMFDMVLLCSNEKFPE
VITTIRETIQNEAASKNSSEITEVKEPGKGRKSRSKKKQPKKEVVDLQTLLMQCAQTVAAG
DHQRAYDLLRQIRQHSNPHGDAWQRLAHYFADGLEARLAGTGSVIYNALVSKRKTASDV
LKAYQIYLAASPLKMVSYHFSNQTIINAIGKATRVHILDFGIYFGFQWPPFMKMLSALPGG
PPKLRITGIDVPQPGFRPEERIEETGRRLHHYAERFHIPFAYQVLASKLDEVKVEDLHLEEE
EVLIVNCVFRINTLGDETMNVDCPRDKFLNNIRKLNPALFVNVATNGTLGAPFFVTRFREA
LYHFSSLFDMLEVTMPREHEQRMLVERDLYGKNAINIISCEGADRLERPETYKQTQVRCLR
AGFEQLPISSEITKMVKSRVRVHYHKDFSVDEDGKWLLLGWRGRIFICLSAWKPSYYLSS
>DchGRAS25
MGESSYGQESSANKSITTSLEIDGLLAGVGYAIRPSDLPHVAERLEKLDSAIIAAAAAGLPP
EFLCSGAVHYNPSDLAAWVDSMSMLTSLSSPPIHPAPAPWPVQIYAPAPLSTPTVDLLAPED
AGINLVHLLMSCADSIHRDDHPSAANLIHEMQILLPRVGTEFGIGKVGAYFIDALDRRLRS
RPSPPAEDEILYHYFYESSPYLRFAHLTANQAILESFNGLDRVHVIDLNLKHGLQWPALIQA
LALRPGGPPFIRLTGFGPPAICEVGRRLADFARSVNVPFAFTLLDEVQPNLLRVEPGETVAV
NSVLQLHQFLSDPGSPGPIDSVLSWVKALRPRIVTVVEQEADHNRPGFLDRFTEALFYYST
VFDSLESAAPGAGPEVYLQKEICNIVCCDGEERVERHEPLASWRARFGRAGLWPVPHGSS
AFRQASMLLTLFSGEGYSIEEVEGCLTLSWHGRAVISTSAWHCGDGVDAPPLAGNICF
>DchGRAS26
MQRQKMMTGRKFFAEEEDVSFLLPMKGVPFNLQYWNGALQIAESQQTGTVVEEKGSAF
WVPAGHKRTKGGVLEPLSSLNQTRSPSSPSSTSTLTSLGGGSAGSIDTAGAAVVFNNVALK
CPNSNFTSTAAANYLNDWMLSDSTASPGQEQTFLHLIMGDLEDKGLGLIDSVVGFAPISFA
SLASSDDLFQISCDSNRNNPSSLQRQPPCVKNPNFSPLATSVTSPPQISLPPPMFFQESMVEK
SQLFGPNFFFHPYQPNEDSLNTGIFFPLPSCTPSQQPDQSLNFTQVHHFPYHQQQQQQVVV
DMLFKAAELVESGNFVGAHVILARLNHHLPSRIGKPLLRSAFYFKGALQVLVNSVNPQAT
ASSPLDVVLKLSAYKAFSEVSPIPQFICFTATQALIEELGAADCIHIIDFDISFGTQWSSFMQE
LAQRQSPRILLLKITAFVSPSAFHPLELHLIKENLCRFAADLNIPFEIRITNIEAFDPAEILILSA
GTNEAIAVNLPVEAWQVPTFATLLGLVKQLLPKIVISVDHGFDRFGLPFSHHFLHALQSCV
TVLDSIDAAGTNFDVISKIEKFVLQPKIENCIIRCHRATEKSLPWLTLFESAGFKPVQFSNFT
EMQAECLLKRMQISGFSVEKRRASLHLLWQEGELASVSVWRS
>DchGRAS27
MEEDTLPLQISHYFSTTFNADIGWAHNSPATDWSSFSGNGLAIPFYGFDDPIFSSLLSENPPL
VSASDEKSLRILHLLMAAAEALSGPQKQRELAGVILFRLKELLVPTESAATIVERLAVHFTK
ALQSLLAGKGPSFHRHPAADELPALQLMQDMSPYISFGYLTANQAILDAVSGFQQVHIVDF
DIKEGLQWPSLIQALVSHKNGNFTSPSLRITAVASGKWSMAKVEETGQRLTAFAASVSLPFS
FDHCRLGRNQRFRPAAVRTVKGDAVILNCVLQWPQTPNRTATSVKSFIGGAAAVGACLVT
VVEEENDGGEATVTAAAGFTGKFMDEMKRHWAMMEALEEGFPKQRMAREMVEKEILA
PRIAAAVWKAYCEEEGGVGEWMAAAGFRRVDLSGFNVSQARMLIKLFNYGYRVEEENP
NKLVLIWKSRRLVCATTWAVPPAAAGEAARETCL

>DchGRAS28
MKTEHVESCRGGGIGGFLPSKGKMMAMEETDAGMDEQLAALGYKVRASDMANVAQRL
EQLDMAIGGSAAQDDALLSHLASDTVHYNPSDISNWLENMLSELNAPPIPAPTATHPKSIS
FDFPSEDLSSSVPNPLDPDYCLRSDYQVEGRSRNRKRIRTSSSSSSSSRTAAPAIESIPAVVAA
TEVSSTNPVVVMVDSQEAGIRLVHALLACAESLQQENYKAADAILKQISILASSQAGAMR
KVAGYFAEALARRIYRLHPTQDTFLDSAFSDILEMHFYESCPYLKFAHFTANQAILEAFSGC
RRVHVIDFGIKQGMQWPALMQALALRPGGPPSFRLTGIGPPSPDNSNALQQVGWKLAQL
ADAIHVDFEYRGLVTNNLADLEPFMLESDESEAVAINSVFELHNLLARPGAIEILLSTVRSL
RPRIMTVVEQEANHNAGSFLERFTESLHYYSTMFDSLEGVAGGEGQQDQVMSEVFLGRQI
CNVVACEGPERTERHETLAQWRVRMDGAGLRPVNLGSNAFKQASMLLALFAGGDGYRV
QENEGCLTLGWHTRPLIATSAWCVAGAGESG
>DchGRAS29
MDGKLRRSGYRLRSVHHLVVNGEVDTSFQRSLARRLQKLNELDQEILGYIDRRIQASSLR
GFTAEEMLHFKRSMEMVDAYAQLQTLNFQPNASRFSSKLQRTDKIEVPNAKNEPHNESSE
NRLWREIIGLSSQKENTQTEDQSIELVSFLTHCAEAIITGDHASINYFLSRLGEMASPTGSTV
HRLAAYFTEGLALSAVKLWPHVFSINPPRDVIEPIDDDEATALRLLDQATPIPKFIHFTLNER
LMRAFEGKDKVHIIDLDIKQGLQWPSLFQSLASRPNPPIHVRVTGIGESKPELQNTGARLA
ALADSLNLPFQFHAVVDQLEDVRLWMLHVKEGECLAVNCVLQLHKTLYDDTGGSLRDL
MSVIGSTNPAIVLVAEEEADHNHHLLQLRFANSLKYYAAIFDALNVSLPEDSSERAKIEKMF
ARKIRNIIAREGTERHENFSQWSRKMKDCRFKNVGIGDREKVQSQVILRMYSDSSYSVEKI
EEDGEALCLKWQEQPLYTVSAWAPMDIVGSSTAAQPD
>DchGRAS30
MAGIADELSSSVTSSPLASFMSISPTSLGSPYAPWLRDLKSDERGLFLIHLLLNCATHVAAG
NIDQANAFLEQISFLSSPDGDTMQRIASYFTEALARRLLRSWPGLYRALNPDLPESSSASTF
HARRLFLDLFPFLRLSFLVSNQAILEAMEGEKVVHVVDLGGSDPSQWIALLQSLAARQEG
PPHLRITAVGESRDFLTHMASILSKEAEKLDIPFQFHAVISRLDTLNVESLRVKTGEALAITS
LLQLHPLLAPNDDAAAPRKHFSTKIESFLTSLWGLSPKIMVVTEQESNHNGSTLKERFMEA
LYFYAAMFDCLESTVPRQSAERARVEKMLFGEEIKNIIACEGLERKERHEKLDRWMQRLE
MAGFWRVGLSYYGLMQARRLLQGFGSDGYQVKEQNGSFFFCWQERALFSVSAWRFRR
>DchGRAS31
MPPIAGGLLETKGISTRTITSLLRQSIPTPSTKSLVGSMSMQASKKYKNFDKPQQFYMNSSN
SARESESHYLPQKHQLVNHLCSNDTFGSRNSPHQVSDIQYCTLESSSVAGSYGIRQSPSISL
SSTTGSSVSQHDSLSDNTYGSPASVSCLTEDPNDLKHKMRELETAMLGSEMDNGDSFETT
FDRQLFLEPEKWKLLEIVRGDLKKGLIACARAVEDDDLMMAELLMTELRQMVSVSGEPI
QRLGAYLLEGLIARVASTGSSIYKALKCKEPTSSELLSYMHILYEVCPYFKFGYFSANGAIA
EALKGENAVHIIDFQIAQGSQWISLIQALAARPGGPPRVRITGIDDSISEYARGSGLHVVGQ
RLSRLAQSCNVPFEFHAAPLSGDKVHVEHLFVHPGEALAVNFAYQLHHMPDESVSTENHR
DRILRMVKSLSPKVVTLVEQESNTNTAPFFPRFLETLDYYTAIFESIDAVLPRDHKERISVEQ
HCLARDIVNVIACEGAERVERHELLGKWKSRFRMAGFSPYPLSPFVNATIKSLLENYCEHY
KLEDRDEALYLGWKNRVLSVSCAWL
>DchGRAS32
MPPIAGGLLETKGISTRKITSLLRQSIPTPSTKSLVGSMSMQASKKYKNFDKPQQFYMNSS
NSARESESHYLPQKHQLVNHLCSNDASGSRNSPHQASDIQYCTLESSSVAGSYGIRQSPSIS
LSSTTGSSVSQHDSLSDNTYGSPASVSCLTEDPNDLKHKMRELETAMLGSEMDNGDSFET

TFDRQLFLEPEKWKFLEIVRGDLKKGLIACARAVEDDDLMMAELLMTELRQMVSVSGEPI
QRLGAYLLEGLIARVASTGSSIYKALKCKEPTSSELLSYMHILYEVCPYFKFGYFSANGAIA
EALKGENAVHIIDFQIAQGSQWISLIQALAARPGGPPRVRITGIDDSISEYARGSGLHVVGQ
RLSRLAQSCNVPFEFHAAPLSGDKVHVEHLFVHPGEALAVNFAYQLHHMPDESVSTENHR
DRILRMVKSLSPKVVTLVEQESNTNTAPFFPRFLETLDYYTAIFESIDAVLPRDHKERISVEQ
HCLARDIVNVIACEGAERVERHELLGKWKSRFRMAGFSPYPLSPFVNATIKSLLENYCEHY
KLEDRDGALYLGWKNRVLSVSCAWL
>DchGRAS33
MKRENLDGVARGGIVRNVAPAPARMAMMGKAKMMGFDEETEDEHLAVLGYKVRSSDM
EIVAQKLQQLEMAMGAGAPQDDALLNHLAADTVHYNPSDISNWLESMLSELSTPPPPLPP
VMHPNSGVFDFPPAPMSSHSSSNFFNSAETLATMESSTVTSIELSTPSAAMIPADYSLRSNS
AGGNRAAYGSETQMENTGPRDRKRMKTSSSSSTSRGGSGGVIGRSVPAIGSMSTSTCGSE
VSSAVPVVMADSQEAGIRLVHALMACAEAVQQENYKASDILVKQISMLASSQGGAMRKV
AGYFAEALARRIYCLNPQQDCSLDSAFSDILQMHFYESCPYLKFAHFTANQAILEAFAGYR
RVHVIDFSMKQGMQWPALMQALALRPGGPPSFRLTGIGPPQPDNTDALQQVGWKLAQLA
DTIHVDFEYRGFVANSLADLEHYMLESPSSANSGSDEPEVVAVNSVFELHGLLARPGALEK
VLGTVRALKPRIVTIVEQDANHNAGPFMDRFTEALHFYSTMFDSLEGGGIADGQQDQFM
SEVYLGRQICNVVACEGTERTERHETLSQWRGRMKDADFEPVHLGSNAFKQASMLLALF
AGGDGYRVEEKDGCLTLGWHTRPLIATSAWRPAGSGESFSAPAASDAR
>DchGRAS34
MELVLDDLPAFIFSAGSSTTTTTTTTTTTTTVNNDISWDDWPSPHLNWSHEDFHPFFAAAG
DHYFATPPHNIQNQSSSTSSTTTTPEEDPTPPTKFPVESDKTQRLLHLLTAAAEALSGDLNG
QDLARVILVRLKELLSTDNAGSGIERLTNHFTEALNALLDGDATFPGRSEFNQFPYRHYPA
AEVLTAFQLLQDMSPIASFGHLTANQAIIEAISGERRVHIVDYDIAEGIQWASLIQAMASRN
DGLPPPHLKITAVTNGRRRSVSAAQETGRRLSDFAASVRQPFSFRLCRLDQQKRFQPAAVK
VVKGEPVVVNCVLYSGQVGYGHGSVTSVGSFLLGAVALRARLVTLIEEDIGAAAGEEKGS
FLQRFMIELERYMAIWESLEAGFPKQGRVREMVERVILGPRITGAVRRAYGRNGNCEPVAE
ECGEWMAAAGFARVGLSFFNLCQARLLLGLFNDGYKVEDDASNKLLLSWKSRRLLSASV
WTIPTRMSPSPSPSLAI
>DchGRAS35
MEVDMEYMMELAFSGCSSTSSTTGFGDLAWVDWSPAVDWGHFSVENDFNGLLEHYGLP
CTPATHDWSTNVSPTSTNCPGTPAEEFQPQVPPPMEFERDNDRGLRLVHLLMAAAEALTG
ANKSSELARVILARLRELLAQGGATNMERLAEHFTDALQRLLDGVGRHDDQPPNSGEVL
EAFQLLQDMSPYVKFGHFTANQAILESVAGERRVHIVDYDIMEGVQWASLMQAMVSQQE
TPPLAPPPHLRITAVTRSGGSRRSGTTVLDTGRRLAAFAASVGLSFSFGQCRLDADDQLRP
AAVKVVKGEAVVLNCALHPPHLPWRSAASIASFLGSAGELGARIVTVVEEAVVVGGSDDS
GGFVGSFMEEMKRYSAMWDSLEAGFPMQGKARGVVEKLILGPMIAGSVGRAYRRREEG
GEEREGWAEWMEETGFQRIGLSLFNLCQSRLLLGLFNDGYKVDDEGPNKLVLCWKNCRL
LSASVWSAPAIESPAASGFSW
>DchGRAS36
MQNSSQFLQQEDPNATKKWAVNLLKECAKAIFEKDSVKIHHILWMLNELASPYGDCEQK
LAFYFLQALFYKLTESGERSYKTLVSAAEKSQSFDSARKVILKFQEVSPWTTFGHVASNSA
ILEAFEGKSKLHIIDISNTYCTQWPTLLESLATRNDDTPHLKLSVVVTDGARGSIMKEIGYR
MQKFARLMGVPFEFKTISFFSRLSDLDRDKFGVKEDEAVAVNCIGAFRKLGVEERSEFIQM

VRSLQPRVVTVVEEEADFSSKEDEFFKCFEECLKFYSMFFEMLEESFAPTSNERLMLEREC
SRTIIKLLACGRDDSEPERREKGIQWCNRLLEAFSPACFSEDTIDDVKALLKRYQAGWSLLP
ASGDASGLYMTWKQEPVVWASAWKL
>DchGRAS37
MTLEEGLDASNLSSHHGNHHHHHLMDWLEDSISFLPTFPNEPYSVPEEIDHCQWWVQDQ
NQPLTAAAAVAAVAAAPSIAIVTAKPEPLPEMPKAELPKKRKLAARQTASVFEEERKPQVN
NGSKKGHGRGSMSNSGSGGGGGASKDVRWAEQLLNPLAAAIDAANLSRAQHLIYVLQE
LASPTGDANHRLAFYGLQVLSRNFTSSLSSPLTYSSTDPRLFRSALIKFHEVSAWFAFPNCL
ANAAILQAAMGGRAGQVRSGSLHVVDVGVSHGLQWPTLMEALTRRGMGLVGQGMVKL
TVVETGSGGPFGSAPAGYDFVPQLMRYGKSIDLNLRVERGEVGRGPGKEIVVVCLQFRAG
RVGPGFWKAVREMEPDLVVLTEMEEGRGRGEENGFGRRAEMLWGFLESTSVAFKGRDSE
ERRVVEGEAARLLEEEGEEGEGWGNERWKEKMEGEGFKEEGFGEEAVEAGRALLKKYD
GNWEMKIGTDGAVGLSWKGQTVSFCSLWKPAYKTTAGVLSSGGRRH
>DchGRAS38
MELLGNGGGKVKQEYPMGKRKMMAMDQDMDAGMDELLAELGYKVRSSSMADLAQK
LELLDRAMGNSSALDDIFLSHLASDSVHYNPSDMSRCVDNMLSELDSPPSATFPSSRVVDL
SPINVSSWLGYPVQPTLSIQSDCGSEPHLGMESREWKRIRTSSSSTACSGGLIRPSEVVYSSA
MPMMMVESDHEAGIRLVHALVACAEAIQEENYKSADAIMKQITFLTSSQHGAMRKVGVY
FAEALGRRIYNLHPKQDHYLDSAFSDILQMHFYETCPYLKFAHFTANQAILEAFAGHRRVH
VIDFGIKQGMQWPALMQALALRPGGPPSFRLTGITPPQSDNSDSLQQLGLKLAQLARTINV
EFEFRGLVANSLADLEPYMLDVEPEVIAINSVFELHGLLARPGAIEKVLDTVRSLRPRIVTI
VEQEANHNAHGFVERFTEALHYYSTLFDSMDGGSDEVSPEKLMTEVFVGRQICNVVACE
GFERTERHETLEQWMLRMRGAGFEPANLGSNAFKQASMLLALFGRGDGYRIEEKEGCLT
LGWHNRPVIATSAWLVAGAGDGVEAAR
>DchGRAS39
MDVAMANAADLYYSGYNAAAATNVGWLNWSPAIGWLSISPEEDLHDEKEAMAWQYSD
DYALAGSSPETLDHWSSSTVSEMTLSSAAGTPAEEPLKLSSVGEIDKGIRLIHLLTAAAEAL
AGDQKSHELSKVILARLKELLPSPASGSSMERLAGHFAKALHCLLVDGEAEDNCSGIETTA
ITAASQLLQDMSPYVNFGHFTANQAIVEAVGGERRVHIVDFDIMEGAQWAPLLQAFMSM
KDGPPPSHVKITAVTAGRKSASAAVQETGRMLAAFAASISLPFSFAQCRLDRDGCFRPAAL
KVLRGEAIVFNCALHAPHHHHHSPASIRSFLASAADFGARLVTVVEEEGRDTAGGGKGGF
LVGFMEELQRYSTLWDALEEGFPKQSKAREMVENLVFGPRIAGAVEEAFRRREDDVTEC
WSEWMAAADFRKVALSFFNQWQAKLLLGLFNEGYRVEEEAPNKLVLGWKSRRLVSASV
WSAQPPPADAED
>DchGRAS40
MGRNPYHPQPTDEDSSSASETDTLLAGVGYTVRSSDLLHVADRLEKLDSTIIAAVATGVLP
EFLCSSAVHYNPSDLASWIDSLLTEVPPRSSPSMWTDAICLPPPSSFSIDAHRLGEEEDAGIH
LVHLLMTCADSIQREDHVLARSLIDEMRLLLPLIGVGYGIGKVSIFFLDALDRRISFLPQPT
KSVLEDEILYSHFYEACPNLKFAHFTANQAILEAFNGCDPVHVIDLSLMHGLQWPALIQAL
ALRPGGPPALRLTGIGPHTPKVGVRLAELARSVGVPFTFHAVAAGRLDSVRVKPGEAVAIN
SVLQLHLLLSETGPIDSVLTLIRELRPKILTVVEQEADHNKPAFLDRFTEALFYYSTLFDSLA
ASGGGRVDGGCSAAAEVYLQQEICNIVCCEGSERVERHEPMVRWRVRLGRAGFEPMRLG
SNAFRQASMLLKLFSSEGYAVEEVEGCLSLSWHGRSLISASAWRARADSAVAPHACHRYI
VL

>DchGRAS41
MQASDNRRNSGKSQQSHSLQNSMHLRESGPNYLPQNFKSSRHGSQMRKISPQSSNVQYC
TLESSSAVATHTYSSPPSTASFSSASESQGSHQDCHSDNASESPVSASCVTEDLNDLKGKLK
ELESVMLGPELDAVDSFDTFELLMELPSGDLRQVLTACARAVGENNFSAAEWLITELRQM
VSVSGEPIERLGAYLLEGLVARLSSSGSSIYKALKCKEPTSSDLMSYMHILYEVCPYFKFGY
MSANGAIAEALKNEDRVHIIDFQIAQGSQWVTLIQALAARPGGPPHVRITGIDDSFNAYAR
GGGLHIVGQRLSRLAESCKVPFEFHPALMSGCEVEVENLGIRPGEALAVNLAFQLHHIPDE
SVSTTNHRDRILRLVKSLTPKVVTLVEQESNTNTAAFFPRFMETLDYYTAIFESIDVTLPRE
NKERINVEQHCLARDIVNIIACEGIERVERHEVHGKWKSRFKMAGFTPYPLSSLVNATIKSL
LENYCDKYRLEEIDGALYLGWMNRALVVSSAWR
>DchGRAS42
MEQLLAHCAHAIDSHDATLAQQLLWVLHNIAPPDGDTNQRLTSAFLRALLLRASRSSCPA
LSSINHSPPSHLPLSATSLAAFIDLTPCHRFGFSAANSAIAEATDSFPILHIVDLSTTHGMQLP
TLIETLAARRPESPPFLRVTIPSLPPDTSPPPTLEIPLQELGARLVNFARSRNLAMEFNIVQSN
PADGFSSLIEHLRLQQLVAEPGAEALVINSHFMLHFTPDESSAPPNISQSHSNIVPRTMFLNA
LRGLDPTILIVVEEDADFTACNVVERLRAAFNYMWIPFDAVDSFLPKGGEQRRRYEEAVC
WKIENAIAQEGLERVERLETRSKWVQRMRGVGFRGVGFGEEAVGELKSMVDEHSAGWG
VKKDEEEILVLTWKGHDVLFASAWMPAPPPPLPAPPPLIL
>DchGRAS43
MELRASSSGPTELRTLGGGLAELKALGSGLVELRKSSGGPAELRMSGGGPAELRTSGGGPA
ELRTSGGGPMELRGFLPPSPETEAGDRSQHRRRMASGFPGRDLRGGFGFGAGSAVQMQY
QQRSDGGAAIGAAGLLKRSLTEMERQQQMQHAIYLRSVKQKTHFASPTPVSHISAADLSA
ASTTTTSSILSSLSSASASVPFVGQGIPNSTTWSFQATFADAEIASEKKSSMTTRLLELERQL
LDDEEEELSISGSAVTSNEWSETMEKLMSPPPPPPPIAAVVNLYPLSPSPTTSSTSSSGSSSSC
TPSSATPSRQMLLDTVTAITEGNQETATANLAVLKRTANPRGDPEQRLTAMMVAALVSRIN
PPAPGSSLPVADVCSAEHQAASHLLYEASPCFKLGLMAANLAILEATKDNPKIHILDFDIGQ
GSQLASLIHAFSERHRQRSPALPPPCIKITSVCPLQGSDTTNLRIVGDRLAKLAESRGVGLR
FNIVNSRTQELNQTTLLCEPGEALAVNFAFFLSRVADESVSTANPRDELLRRVKSLSPRVVT
LVEQEMNGNTAPFATRFTEACAHFGALLESLDATAARESTERARVEACLARKAANSIARE
GADRVERCELYGKWRARMGMAGLNPVPLGSIVSEPVKLRLSSLRNNPGFTIKEEAAWLG
CGWMGRVLTVASAWR
>DchGRAS44
MRGLPFNYQRKGTVLLLESQARGTGVEEKTGVLWSSSVQNRKKGVILEPRSVLDQRSPSP
PTSTSTLSSPLGGGAARFTDTAGVAAVSGKTTFKWPNTAGEEGGRKEEWAPELHPVPASLE
MDWDNMLSDSAASPGQEQTFLGWIMGDIDDPAKQQHLMLSSPTSMAEIDSGSHGVGFGL
IDPGFGFESNASASAISSQPPPQLDSSPGEKNPIFTASLPLSLPPAMFFQETTEEKPQFFGSNF
LLNQFQQTQVPANPAFFVPLPSFSSSMENLVSTPHPGAGSELFLRSQPNQGLNFHQLHPVP
YHLQHRPLKPKSLSAGDEAIAIQQQQQQQALVDQLLKAAELVEAGNLVGAHGILARLNH
QLPSAFGEPFLRSAFYFKEALQLLVNSANPQAATSSPSHHHRTPFTSPLASPLDVVLKLSAY
KAFSEVSPILQFTNFTATQALLEEIGAADCIHIIDFDIGVGGQWSSFMQELAQRRSSSSAISM
LKITAFVSPYAFHPLELHLTKENLSHFAADLNIPFEINIASIESFDPAEILVLSAGSNEAIAVNL
PVGVWQGPSFPTLLRLVKQLLPKIVISMDQGCDRGDLLFSHYLRHALQSCIVLLDSFDAA
GTDQDLTNKIERFVLQPRIENCVIGRHCTAEKMLPWRTLFASAGFVPFQFSNFTEAQAECL
LKRVQVRGFHVEKRQAALYLYWQRGELASVSAWRC

>DchGRAS45
MAYMCADSGNLMAIAQQVIKQKQQQQRHQHQQQNQEPVIPAIDVASSDGDPFAAVPPQ
WGNQHQQTTPIRDHFPFALPEHTFHDPFAAGVFGLPPPTHNDHAPFRIPEFDSDEWMESLI
GESPTESSEIMADVWQRTDLPPIFPDAFASCSTAISLPSQASTSDLDRVVFPDSCKIVAPHSLP
FDPPPPPQPKKDAVDSKSPPLLFKSLLDCARIIDTEPDQAAKTIDHIRDLASEHGDPTERVAF
YFAEALNSRLTGCSDPDFREEEIALCYRAFNDACPYSMFVHLTANQAILEATESAEIIHIIDF
GISQGIQWAALLQALATRPTGKPTRIRISGIPAASLGGSPATSLAATGNRLLDFARLLDLDFE
FEPVLHPVQDLTAASFRVEPGEAVAVNFTLQLYTLLGDSDEAVNRVIRLAKSVRPSVVTLGE
YECSLNRIGFVERFRNALEYFAAVFDSVESSRGRDSDERARIERVILGPRILYTVVPEDGRQ
RRMRMEAKEKWRVIMEGCGLEMVPLSNYAVSQAKLLLWNYDYSPKYKLLDSSTGILSLA
WEDLPLFTVSSWR
>DchGRAS46
MSLIKRSSDILTGESKQAACVGLQLKKCSISRTDKIEVPNAKNEPHNESSENRLWREIIGLSS
QKENTQTEDQSIELVSFLTHCAEAIITGDHASINYFLSRLGEMASPTGSTVHRLAAYFTEGL
ALSAVKLWPHVFSINPPRDVIEPIDDDEATALRLLDQATPIPKFIHFTLNERLMRAFEGKDK
VHIIDLDIKQGLQWPSLFQSLASRPNPPIHVRVTGIGESKPELQNTGARLAALADSLNLPFQ
FHAVVDQLEDVRLWMLHVKEGECLAVNCVLQLHKTLYDDTGGSLRDLMSVIGSTNPAIV
LVAEEEADHNHHLLQLRFANSLKYYAAIFDALNVSLPEDSSERAKIEKMFARKIRNIIAREG
TERHENFSQWSRKMKDCRFKNVGIGDREKVQSQVILRMYSDSSYSVEKIEEDGEALCLK
WQEQPLYTVSAWAPMDIVGSSTAAQPD
>AtGRAS1
MESNYSGVVNGYDVSFLPTSIPDLGFGVPSSSDFDLRMDQYYHQPSIWVPDQDHHFSPPA
DEIDSENTLLKYVNQLLMEESLAEKQSIFYDSLALRQTEEMLQQVISDSQTQSSIPNNSITT
SSSSNSGDYSNSSNSSVRIENEVLFDNKHLGDSGVVSFPGSNMLRGGEQFGQPANEILVRS
MFSDAESVLQFKRGLEEASKFLPNTDQWIFNLEPEMERVVPVKVEEGWSAISKTRKNHHE
REEEEDDLEEARRRSKQFAVNEEDGKLTEMFDKVLLLDGECDPQIIEDGENGSSKALVKK
GRAKKKSRAVDFRTLLTLCAQSVSAGDKITADDLLRQIRKQCSPVGDASQRLAHFFANAL
EARLEGSTGTMIQSYYDSISSKKRTAAQILKSYSVFLSASPFMTLIYFFSNKMILDAAKDAS
VLHIVDFGILYGFQWPMFIQHLSKSNPGLRKLRITGIEIPQHGLRPTERIQDTGRRLTEYCKR
FGVPFEYNAIASKNWETIKMEEFKIRPNEVLAVNAVLRFKNLRDVIPGEEDCPRDGFLKLIR
DMNPNVFLSSTVNGSFNAPFFTTRFKEALFHYSALFDLFGATLSKENPERIHFEGEFYGRE
VMNVIACEGVDRVERPETYKQWQVRMIRAGFKQKPVEAELVQLFREKMKKWGYHKDF
VLDEDSNWFLQGWKGRILFSSSCWVPS
>AtGRAS2
MGSYPDGFPGSMDELDFNKDFDLPPSSNQTLGLANGFYLDDLDFSSLDPPEAYPSQNNNN
NNINNKAVAGDLLSSSSDDADFSDSVLKYISQVLMEEDMEEKPCMFHDALALQAAEKSLY
EALGEKYPSSSSASSVDHPERLASDSPDGSCSGGAFSDYASTTTTTSSDSHWSVDGLENRP
SWLHTPMPSNFVFQSTSRSNSVTGGGGGGNSAVYGSGFGDDLVSNMFKDDELAMQFKK
GVEEASKFLPKSSQLFIDVDSYIPMNSGSKENGSEVFVKTEKKDETEHHHHHSYAPPPNRL
TGKKSHWRDEDEDFVEERSNKQSAVYVEESELSEMFDKILVCGPGKPVCILNQNFPTESA
KVVTAQSNGAKIRGKKSTSTSHSNDSKKETADLRTLLVLCAQAVSVDDRRTANEMLRQIR
EHSSPLGNGSERLAHYFANSLEARLAGTGTQIYTALSSKKTSAADMLKAYQTYMSVCPFK
KAAIIFANHSMMRFTANANTIHIIDFGISYGFQWPALIHRLSLSRPGGSPKLRITGIELPQRGF
RPAEGVQETGHRLARYCQRHNVPFEYNAIAQKWETIQVEDLKLRQGEYVVVNSLFRFRN

LLDETVLVNSPRDAVLKLIRKINPNVFIPAILSGNYNAPFFVTRFREALFHYSAVFDMCDSK
LAREDEMRLMYEKEFYGREIVNVVACEGTERVERPETYKQWQARLIRAGFRQLPLEKEL
MQNLKLKIENGYDKNFDVDQNGNWLLQGWKGRIVYASSLWVPSSS
>AtGRAS3
MKRDHHHHHHQDKKTMMMNEEDDGNGMDELLAVLGYKVRSSEMADVAQKLEQLEV
MMSNVQEDDLSQLATETVHYNPAELYTWLDSMLTDLNPPSSNAEYDLKAIPGDAILNQFA
IDSASSSNQGGGGDTYTTNKRLKCSNGVVETTTATAESTRHVVLVDSQENGVRLVHALLA
CAEAVQKENLTVAEALVKQIGFLAVSQIGAMRKVATYFAEALARRIYRLSPSQSPIDHSLSD
TLQMHFYETCPYLKFAHFTANQAILEAFQGKKRVHVIDFSMSQGLQWPALMQALALRPG
GPPVFRLTGIGPPAPDNFDYLHEVGCKLAHLAEAIHVEFEYRGFVANTLADLDASMLELRP
SEIESVAVNSVFELHKLLGRPGAIDKVLGVVNQIKPEIFTVVEQESNHNSPIFLDRFTESLHY
YSTLFDSLEGVPSGQDKVMSEVYLGKQICNVVACDGPDRVERHETLSQWRNRFGSAGFA
AAHIGSNAFKQASMLLALFNGGEGYRVEESDGCLMLGWHTRPLIATSAWKLSTN
>AtGRAS4
MVEQTVVREHIKARVMSLVRSAEPSSYRNPKLYTLNENGNNNGVSSAQIFDPDRSKNPCL
TDDSYPSQSYEKYFLDSPTDEFVQHPIGSGASVSSFGSLDSFPYQSRPVLGCSMEFQLPLDS
TSTSSTRLLGDYQAVSYSPSMDVVEEFDDEQMRSKIQELERALLGDEDDKMVGIDNLMEI
DSEWSYQNESEQHQDSPKESSSADSNSHVSSKEVVSQATPKQILISCARALSEGKLEEALS
MVNELRQIVSIQGDPSQRIAAYMVEGLAARMAASGKFIYRALKCKEPPSDERLAAMQVLF
EVCPCFKFGFLAANGAILEAIKGEEEVHIIDFDINQGNQYMTLIRSIAELPGKRPRLRLTGID
DPESVQRSIGGLRIIGLRLEQLAEDNGVSFKFKAMPSKTSIVSPSTLGCKPGETLIVNFAFQL
HHMPDESVTTVNQRDELLHMVKSLNPKLVTVVEQDVNTNTSPFFPRFIEAYEYYSAVFES
LDMTLPRESQERMNVERQCLARDIVNIVACEGEERIERYEAAGKWRARMMMAGFNPKP
MSAKVTNNIQNLIKQQYCNKYKLKEEMGELHFCWEEKSLIVASAWR
>AtGRAS5
MVAMFQEDNGTSSVASSPLQVFSTMSLNRPTLLASSSPFHCLKDLKPEERGLYLIHLLLTC
ANHVASGSLQNANAALEQLSHLASPDGDTMQRIAAYFTEALANRILKSWPGLYKALNAT
QTRTNNVSEEIHVRRLFFEMFPILKVSYLLTNRAILEAMEGEKMVHVIDLDASEPAQWLAL
LQAFNSRPEGPPHLRITGVHHQKEVLEQMAHRLIEEAEKLDIPFQFNPVVSRLDCLNVEQL
RVKTGEALAVSSVLQLHTFLASDDDLMRKNCALRFQNNPSGVDLQRVLMMSHGSAAEA
RENDMSNNNGYSPSGDSASSLPLPSSGRTDSFLNAIWGLSPKVMVVTEQDSDHNGSTLME
RLLESLYTYAALFDCLETKVPRTSQDRIKVEKMLFGEEIKNIISCEGFERRERHEKLEKWSQ
RIDLAGFGNVPLSYYAMLQARRLLQGCGFDGYRIKEESGCAVICWQDRPLYSVSAWRCRK
>AtGRAS6
MRLSVFIIPLVESRQASGIINKQSTSLLIRFSLYLEASISTKSFFSKSQRISQTQSPICLSANYY
QPDNLDMEATQKHMIQEGSSMFYHQPSSVKQMDLSVQTFDSYCTLESSSGTKSHPCLNN
KNNSSSTTSFSSNESPISQANNNNLSRFNNHSPEENNNSPLSGSSATNTNETELSLMLKDLE
TAMMEPDVDNSYNNQGGFGQQHGVVSSAMYRSMEMISRGDLKGVLYECAKAVENYDL
EMTDWLISQLQQMVSVSGEPVQRLGAYMLEGLVARLASSGSSIYKALRCKDPTGPELLTY
MHILYEACPYFKFGYESANGAIAEAVKNESFVHIIDFQISQGGQWVSLIRALGARPGGPPN
VRITGIDDPRSSFARQGGLELVGQRLGKLAEMCGVPFEFHGAALCCTEVEIEKLGVRNGE
ALAVNFPLVLHHMPDESVTVENHRDRLLRLVKHLSPNVVTLVEQEANTNTAPFLPRFVET
MNHYLAVFESIDVKLARDHKERINVEQHCLAREVVNLIACEGVEREERHEPLGKWRSRFH
MAGFKPYPLSSYVNATIKGLLESYSEKYTLEERDGALYLGWKNQPLITSCAWR

>AtGRAS7
MLTSFKSSSSSSEDATATTTENPPPLCIASSSAATSASHHLRRLLFTAANFVSQSNFTAAQNL
LSILSLNSSPHGDSTERLVHLFTKALSVRINRQQQDQTAETVATWTTNEMTMSNSTVFTSS
VCKEQFLFRTKNNNSDFESCYYLWLNQLTPFIRFGHLTANQAILDATETNDNGALHILDLDI
SQGLQWPPLMQALAERSSNPSSPPPSLRITGCGRDVTGLNRTGDRLTRFADSLGLQFQFHT
LVIVEEDLAGLLLQIRLLALSAVQGETIAVNCVHFLHKIFNDDGDMIGHFLSAIKSLNSRIVT
MAEREANHGDHSFLNRFSEAVDHYMAIFDSLEATLPPNSRERLTLEQRWFGKEILDVVAA
EETERKQRHRRFEIWEEMMKRFGFVNVPIGSFALSQAKLLLRLHYPSEGYNLQFLNNSLFL
GWQNRPLFSVSSWK
>AtGRAS8
MLAGCSSSSLLSPTRRLRSEAVAATSATVSAHFPMNTQRLDLPCSSSFSRKETPSSRPLGRSI
SLDNSNNNNNKPIERKTKTSGCSLKQNIKLPPLATTRGNGEGFSWNNDNNNRGKSLKRLA
EEDESCLSRAKRTKCENEGGFWFEHFTGQDSSSPALPFSLTCSGDDEEKVCFVPSEVISQPL
PNWVDSVITELAGIGDKDVESSLPAAVKEASGGSSTSASSESRSLSHRVPEPTNGSRNPYSH
RGATEERTTGNINNNNNRNDLQRDFELVNLLTGCLDAIRSRNIAAINHFIARTGDLASPRGR
TPMTRLIAYYIEALALRVARMWPHIFHIAPPREFDRTVEDESGNALRFLNQVTPIPKFIHFTA
NEMLLRAFEGKERVHIIDFDIKQGLQWPSFFQSLASRINPPHHVRITGIGESKLELNETGDR
LHGFAEAMNLQFEFHPVVDRLEDVRLWMLHVKEGESVAVNCVMQMHKTLYDGTGAAIR
DFLGLIRSTNPIALVLAEQEAEHNSEQLETRVCNSLKYYSAMFDAIHTNLATDSLMRVKVE
EMLFGREIRNIVACEGSHRQERHVGFRHWRRMLEQLGFRSLGVSEREVLQSKMLLRMYG
SDNEGFFNVERSDEDNGGEGGRGGGVTLRWSEQPLYTISAWTTGGN
>AtGRAS9
MKREHNHRESSAGEGGSSSMTTVIKEEAAGVDELLVVLGYKVRSSDMADVAHKLEQLE
MVLGDGISNLSDETVHYNPSDLSGWVESMLSDLDPTRIQEKPDSEYDLRAIPGSAVYPRDE
HVTRRSKRTRIESELSSTRSVVVLDSQETGVRLVHALLACAEAVQQNNLKLADALVKHVG
LLASSQAGAMRKVATYFAEGLARRIYRIYPRDDVALSSFSDTLQIHFYESCPYLKFAHFTAN
QAILEVFATAEKVHVIDLGLNHGLQWPALIQALALRPNGPPDFRLTGIGYSLTDIQEVGWK
LGQLASTIGVNFEFKSIALNNLSDLKPEMLDIRPGLESVAVNSVFELHRLLAHPGSIDKFLST
IKSIRPDIMTVVEQEANHNGTVFLDRFTESLHYYSSLFDSLEGPPSQDRVMSELFLGRQILN
LVACEGEDRVERHETLNQWRNRFGLGGFKPVSIGSNAYKQASMLLALYAGADGYNVEEN
EGCLLLGWQTRPLIATSAWRINRVE
>AtGRAS10
MKRDHHQFQGRLSNHGTSSSSSSISKDKMMMVKKEEDGGGNMDDELLAVLGYKVRSSE
MAEVALKLEQLETMMSNVQEDGLSHLATDTVHYNPSELYSWLDNMLSELNPPPLPASSN
GLDPVLPSPEICGFPASDYDLKVIPGNAIYQFPAIDSSSSSNNQNKRLKSCSSPDSMVTSTST
GTQIGGVIGTTVTTTTTTTTAAGESTRSVILVDSQENGVRLVHALMACAEAIQQNNLTLAE
ALVKQIGCLAVSQAGAMRKVATYFAEALARRIYRLSPPQNQIDHCLSDTLQMHFYETCPY
LKFAHFTANQAILEAFEGKKRVHVIDFSMNQGLQWPALMQALALREGGPPTFRLTGIGPPA
PDNSDHLHEVGCKLAQLAEAIHVEFEYRGFVANSLADLDASMLELRPSDTEAVAVNSVFE
LHKLLGRPGGIEKVLGVVKQIKPVIFTVVEQESNHNGPVFLDRFTESLHYYSTLFDSLEGV
PNSQDKVMSEVYLGKQICNLVACEGPDRVERHETLSQWGNRFGSSGLAPAHLGSNAFKQ
ASMLLSVFNSGQGYRVEESNGCLMLGWHTRPLITTSAWKLSTAAY
>AtGRAS11
MDNVRGSIMLQPLPEIAESIDDAICHELSMWPDDAKDLLLIVEAISRGDLKLVLVACAKAV

SENNLLMARWCMGELRGMVSISGEPIQRLGAYMLEGLVARLAASGSSIYKSLQSREPESY
EFLSYVYVLHEVCPYFKFGYMSANGAIAEAMKDEERIHIIDFQIGQGSQWIALIQAFAARP
GGAPNIRITGVGDGSVLVTVKKRLEKLAKKFDVPFRFNAVSRPSCEVEVENLDVRDGEAL
GVNFAYMLHHLPDESVSMENHRDRLLRMVKSLSPKVVTLVEQECNTNTSPFLPRFLETLS
YYTAMFESIDVMLPRNHKERINIEQHCMARDVVNIIACEGAERIERHELLGKWKSRFSMA
GFEPYPLSSIISATIRALLRDYSNGYAIEERDGALYLGWMDRILVSSCAWK
>AtGRAS12
MGSYSAGFPGSLDWFDFPGLGNGSYLNDQPLLDIGSVPPPLDPYPQQNLASADADFSDSV
LKYISQVLMEEDMEDKPCMFHDALSLQAAEKSLYEALGEKYPVDDSDQPLTTTTSLAQLV
SSPGGSSYASSTTTTSSDSQWSFDCLENNRPSSWLQTPIPSNFIFQSTSTRASSGNAVFGSSFS
GDLVSNMFNDTDLALQFKKGMEEASKFLPKSSQLVIDNSVPNRLTGKKSHWREEEHLTEE
RSKKQSAIYVDETDELTDMFDNILIFGEAKEQPVCILNESFPKEPAKASTFSKSPKGEKPEA
SGNSYTKETPDLRTMLVSCAQAVSINDRRTADELLSRIRQHSSSYGDGTERLAHYFANSLE
ARLAGIGTQVYTALSSKKTSTSDMLKAYQTYISVCPFKKIAIIFANHSIMRLASSANAKTIHI
IDFGISDGFQWPSLIHRLAWRRGSSCKLRITGIELPQRGFRPAEGVIETGRRLAKYCQKFNIP
FEYNAIAQKWESIKLEDLKLKEGEFVAVNSLFRFRNLLDETVAVHSPRDTVLKLIRKIKPDV
FIPGILSGSYNAPFFVTRFREVLFHYSSLFDMCDTNLTREDPMRVMFEKEFYGREIMNVVA
CEGTERVERPESYKQWQARAMRAGFRQIPLEKELVQKLKLMVESGYKPKEFDVDQDCH
WLLQGWKGRIVYGSSIWVPL
>AtGRAS13
MDPNFSESLNGFEYFDGNPNLLTDPMEDQYPPPSDTLLKYVSEILMEESNGDYKQSMFYD
SLALRKTEEMLQQVITDSQNQSFSPADSLITNSWDASGSIDESAYSADPQPVNEIMVKSMF
SDAESALQFKKGVEEASKFLPNSDQWVINLDIERSERRDSVKEEMGLDQLRVKKNHERDF
EEVRSSKQFASNVEDSKVTDMFDKVLLLDGECDPQTLLDSEIQAIRSSKNIGEKGKKKKK
KKSQVVDFRTLLTHCAQAISTGDKTTALEFLLQIRQQSSPLGDAGQRLAHCFANALEARLQ
GSTGPMIQTYYNALTSSLKDTAADTIRAYRVYLSSSPFVTLMYFFSIWMILDVAKDAPVLHI
VDFGILYGFQWPMFIQSISDRKDVPRKLRITGIELPQCGFRPAERIEETGRRLAEYCKRFNV
PFEYKAIASQNWETIRIEDLDIRPNEVLAVNAGLRLKNLQDETGSEENCPRDAVLKLIRNM
NPDVFIHAIVNGSFNAPFFISRFKEAVYHYSALFDMFDSTLPRDNKERIRFEREFYGREAMN
VIACEEADRVERPETYRQWQVRMVRAGFKQKTIKPELVELFRGKLKKWRYHKDFVVDE
NSKWLLQGWKGRTLYASSCWVPA
>AtGRAS14
MITEPSLTGISGMVNRNRLSGLPDQPSSHSFTPVTLYDGFNYNLSSDHINTVVAAPENSVFI
REEEEEEDPADDFDFSDAVLGYISQMLNEEDMDDKVCMLQESLDLEAAERSLYEAIGKKY
PPSPERNLAFAERNSENLDRVVPGNYTGGDCIGFGNGGIKPLSSGFTLDFRNPQSCSSILSV
PQSNGLITIYGDGIDESSKNNRENHQSVWLFRREIEEANRFNPEENELIVNFREENCVSKAR
KNSSRDEICVEEERSSKLPAVFGEDILRSDVVDKILVHVPGGESMKEFNALRDVLKKGVEK
KKASDAQGGKRRARGRGRGRGRGGGGGGQNGKKEVVDLRSLLIHCAQAVAADDRRCAG
QLLKQIRLHSTPFGDGNQRLAHCFANGLEARLAGTGSQIYKGIVSKPRSAAAVLKAHQLF
LACCPFRKLSYFITNKTIRDLVGNSQRVHVIDFGILYGFQWPTLIHRFSMYGSPKVRITGIEF
PQPGFRPAQRVEETGQRLAAYAKLFGVPFEYKAIAKKWDAIQLEDLDIDRDEITVVNCLYR
AENLHDESVKVESCRDTVLNLIGKINPDLFVFGIVNGAYNAPFFVTRFREALFHFSSIFDML
ETIVPREDEERMFLEMEVFGREALNVIACEGWERVERPETYKQWHVRAMRSGLVQVPFD
PSIMKTSLHKVHTFYHKDFVIDQDNRWLLQGWKGRTVMALSVWKPESKA

>AtGRAS15
MPLSFERFQGEGVFGLSSSSFYSDSQKIWSNQDKTEAKQEDLGYVVGGFLPEPTSVLDAL
RSPSPLASYSSTTTTLSSSHGGGGTTVTNTTVTAGDDNNNNKCSQMGLDDLDGVLSASSP
GQEQSILRLIMDPGSAFGVFDPGFGFGSGSGPVSAPVSDNSNLLCNFPFQEITNPAEALINPS
NHCLFYNPPLSPPAKRFNSGSLHQPVFPLSDPDPGHDPVRRQHQFQFPFYHNNQQQQFPSS
SSSTAVAMVPVPSPGMAGDDQSVIIEQLFNAAELIGTTGNNNGDHTVLAQGILARLNHHL
NTSSNHKSPFQRAASHIAEALLSLIHNESSPPLITPENLILRIAAYRSFSETSPFLQFVNFTAN
QSILESCNESGFDRIHIIDFDVGYGGQWSSLMQELASGVGGRRRNRASSLKLTVFAPPPSTV
SDEFELRFTEENLKTFAGEVKIPFEIELLSVELLLNPAYWPLSLRSSEKEAIAVNLPVNSVAS
GYLPLILRFLKQLSPNIVVCSDRGCDRNDAPFPNAVIHSLQYHTSLLESLDANQNQDDSSIE
RFWVQPSIEKLLMKRHRWIERSPPWRILFTQCGFSPASLSQMAEAQAECLLQRNPVRGFH
VEKRQSSLVMCWQRKELVTVSAWKC
>AtGRAS16
MKRGYGETWDPPPKPLPASRSGEGPSMADKKKADDDNNNSNMDDELLAVLGYKVRSSE
MAEVAQKLEQLEMVLSNDDVGSTVLNDSVHYNPSDLSNWVESMLSELNNPASSDLDTTR
SCVDRSEYDLRAIPGLSAFPKEEEVFDEEASSKRIRLGSWCESSDESTRSVVLVDSQETGVR
LVHALVACAEAIHQENLNLADALVKRVGTLAGSQAGAMGKVATYFAQALARRIYRDYTA
ETDVCAAVNPSFEEVLEMHFYESCPYLKFAHFTANQAILEAVTTARRVHVIDLGLNQGMQ
WPALMQALALRPGGPPSFRLTGIGPPQTENSDSLQQLGWKLAQFAQNMGVEFEFKGLAA
ESLSDLEPEMFETRPESETLVVNSVFELHRLLARSGSIEKLLNTVKAIKPSIVTVVEQEANH
NGIVFLDRFNEALHYYSSLFDSLEDSYSLPSQDRVMSEVYLGRQILNVVAAEGSDRVERHE
TAAQWRIRMKSAGFDPIHLGSSAFKQASMLLSLYATGDGYRVEENDGCLMIGWQTRPLIT
TSAWKLA
>AtGRAS17
MLLEETEPPNQTLDHVLSWLEDSVSLSPLPGFDDSYLLHEFDGSQTWEWDQTQDPEHGFI
QSYSQDLSAAYVGCEATNLEVVTEAPSIDLDLPPEIQQPNDQSRKRSHDGFLEAQQVKKS
ARSKRKAIKSSEKSSKDGNKEGRWAEKLLNPCALAITASNSSRVQHYLCVLSELASSSGDA
NRRLAAFGLRALQHHLSSSSVSSSFWPVFTFASAEVKMFQKTLLKFYEVSPWFALPNNMA
NSAILQILAQDPKDKKDLHIIDIGVSHGMQWPTLLEALSCRLEGPPPRVRITVISDLTADIPF
SVGPPGYNYGSQLLGFARSLKINLQISVLDKLQLIDTSPHENLIVCAQFRLHHLKHSINDER
GETLKAVRSLRPKGVVLCENNGECSSSADFAAGFSKKLEYVWKFLDSTSSGFKEENSEER
KLMEGEATKVLMNAGDMNEGKEKWYERMREAGFFVEAFEEDAVDGAKSLLRKYDNN
WEIRMEDGDTFAGLMWKGEAVSFCSLWK
>AtGRAS18
MDAILPVPVDGFRFDTGSGSCCKPRNNLESGTTNRFTCFNESESQSNPSPTESKVCSDYLP
VFKYINDMLMEEDLEGQSCMLEDSLALQAAERSFFEVLQDQTPISGDLEDGSLGNFSSITS
LHQPEVSEESTRRYRHRDDDEDDDLESGRKSKLPAISTVDELAEKFEEVLLVCQKNDQGE
ATEKKTRHVKGSSNRYKQQKSDQPVDMRNLLMQCAQAVASFDQRRAFEKLKEIREHSSR
HGDATQRLGYHFAEALEARITGTMTTPISATSSRTSMVDILKAYKGFVQACPTLIMCYFTA
NRTINELASKATTLHIIDFGILYGFQWPCLIQALSKRDIGPPLLRVTGIELPQSGFRPSERVEE
TGRRLKRFCDKFNVPFEYSFIAKNWENITLDDLVINSGETTVVNCILRLQYTPDETVSLNSP
RDTALKLFRDINPDLFVFAEINGTYNSPFFLTRFREALFHCSSLFDMYETTLSEDDNCRTLV
ERELIIRDAMSVIACEGSERFARPETYKQWQVRILRAGFRPAKLSKQIVKDGKEIVKERYH
KDFVIDNDNHWMFQGWKGRVLYAVSCWKPAKK

>AtGRAS19
MTKTRILNPTRFPSPKPLRGCGDANFMEQLLLHCATAIDSNDAALTHQILWVLNNIAPPDG
DSTQRLTSAFLRALLSRAVSKTPTLSSTISFLPQADELHRFSVVELAAFVDLTPWHRFGFIA
ANAAILTAVEGYSTVHIVDLSLTHCMQIPTLIDAMASRLNKPPPLLKLTVVSSSDHFPPFINI
SYEELGSKLVNFATTRNITMEFTIVPSTYSDGFSSLLQQLRIYPSSFNEALVVNCHMMLRYIP
EEPLTSSSSSLRTVFLKQLRSLNPRIVTLIEEDVDLTSENLVNRLKSAFNYFWIPFDTTDTFM
SEQRRWYEAEISWKIENVVAKEGAERVERTETKRRWIERMREAEFGGVRVKEDAVADVK
AMLEEHAVGWGMKKEDDDESLVLTWKGHSVVFATVWVPI
>AtGRAS20
MAYMCTDSGNLMAIAQQLIKQKQQQSQHQQQEEQEQEPNPWPNPSFGFTLPGSGFSDP
FQVTNDPGFHFPHLEHHQNAAVASEEFDSDEWMESLINGGDASQTNPDFPIYGHDPFVSFP
SRLSAPSYLNRVNKDDSASQQLPPPPASTAIWSPSPPSPQHPPPPPPQPDFDLNQPIFKAIHD
YARKPETKPDTLIRIKESVSESGDPIQRVGYYFAEALSHKETESPSSSSSSSLEDFILSYKTLN
DACPYSKFAHLTANQAILEATNQSNNIHIVDFGIFQGIQWSALLQALATRSSGKPTRIRISGIP
APSLGDSPGPSLIATGNRLRDFAAILDLNFEFYPVLTPIQLLNGSSFRVDPDEVLVVNFMLEL
YKLLDETATTVGTALRLARSLNPRIVTLGEYEVSLNRVEFANRVKNSLRFYSAVFESLEPNL
DRDSKERLRVERVLFGRRIMDLVRSDDDNNKPGTRFGLMEEKEQWRVLMEKAGFEPVKP
SNYAVSQAKLLLWNYNYSTLYSLVESEPGFISLAWNNVPLLTVSSWR
>AtGRAS21
MAESGDFNGGQPPPHSPLRTTSSGSSSSNNRGPPPPPPPPLVMVRKRLASEMSSNPDYNNS
SRPPRRVSHLLDSNYNTVTPQQPPSLTAAATVSSQPNPPLSVCGFSGLPVFPSDRGGRNVM
MSVQPMDQDSSSSSASPTVWVDAIIRDLIHSSTSVSIPQLIQNVRDIIFPCNPNLGALLEYRL
RSLMLLDPSSSSDPSPQTFEPLYQISNNPSPPQQQQQHQQQQQQHKPPPPPIQQQERENSST
DAPPQPETVTATVPAVQTNTAEALRERKEEIKRQKQDEEGLHLLTLLLQCAEAVSADNLEE
ANKLLLEISQLSTPYGTSAQRVAAYFSEAMSARLLNSCLGIYAALPSRWMPQTHSLKMVS
AFQVFNGISPLVKFSHFTANQAIQEAFEKEDSVHIIDLDIMQGLQWPGLFHILASRPGGPPH
VRLTGLGTSMEALQATGKRLSDFADKLGLPFEFCPLAEKVGNLDTERLNVRKREAVAVH
WLQHSLYDVTGSDAHTLWLLQRLAPKVVTVVEQDLSHAGSFLGRFVEAIHYYSALFDSL
GASYGEESEERHVVEQQLLSKEIRNVLAVGGPSRSGEVKFESWREKMQQCGFKGISLAGN
AATQATLLLGMFPSDGYTLVDDNGTLKLGWKDLSLLTASAWTPRS
>AtGRAS22
MPLPFEQFQGKGVLGFLDSSSSPGYKIWANPEKLHGRVEEDLCFVVNNGGFSEPTSVLDS
VRSPSPFVSSSTTTLSSSHGGPSGGGAAAATFSGADGKCDQMGFEDLDGVLSGGSPGQEQ
SIFRLIMAGDVVDPGSEFVGFDIGSGSDPVIDNPNPLFGYGFPFQNAPEEEKFQISINPNPGF
FSDPPSSPPAKRLNSGQPGSQHLQWVFPFSDPGHESHDPFLTPPKIAGEDQNDQDQSAVIID
QLFSAAAELTTNGGDNNPVLAQGILARLNHNLNNNNDDTNNNPKPPFHRAASYITEALHS
LLQDSSLSPPSLSPPQNLIFRIAAYRAFSETSPFLQFVNFTANQTILESFEGFDRIHIVDFDIGY
GGQWASLIQELAGKRNRSSSAPSLKITAFASPSTVSDEFELRFTEENLRSFAGETGVSFEIEL
LNMEILLNPTYWPLSLFRSSEKEAIAVNLPISSMVSGYLPLILRFLKQISPNVVVCSDRSCDR
NNDAPFPNGVINALQYYTSLLESLDSGNLNNAEAATSIERFCVQPSIQKLLTNRYRWMERS
PPWRSLFGQCGFTPVTLSQTAETQAEYLLQRNPMRGFHLEKRQSSSPSLVLCWQRKELVT
VSAWKC
>AtGRAS23
MPLPFEEFQGKGISCFSSFSSSFPQPPSSPLLSHRKARGGEEEEEEVPAAEPTSVLDSLISPTS

SSTVSSSHGGNSAVGGGGDATTDEQCGAIGLGDWEEQVPHDHEQSILGLIMGDSTDPSLE
LNSILQTSPTFHDSDYSSPGFGVVDTGFGLDHHSVPPSHVSGLLINQSQTHYTQNPAAIFYG
HHHHTPPPAKRLNPGPVGITEQLVKAAEVIESDTCLAQGILARLNQQLSSPVGKPLERAAF
YFKEALNNLLHNVSQTLNPYSLIFKIAAYKSFSEISPVLQFANFTSNQALLESFHGFHRLHII
DFDIGYGGQWASLMQELVLRDNAAPLSLKITVFASPANHDQLELGFTQDNLKHFASEINIS
LDIQVLSLDLLGSISWPNSSEKEAVAVNISAASFSHLPLVLRFVKHLSPTIIVCSDRGCERTDL
PFSQQLAHSLHSHTALFESLDAVNANLDAMQKIERFLIQPEIEKLVLDRSRPIERPMMTWQ
AMFLQMGFSPVTHSNFTESQAECLVQRTPVRGFHVEKKHNSLLLCWQRTELVGVSAWRC
RSS
>AtGRAS24
MNYPYEDFLDLFFSTHTDPLATAASTSSNGYSLNDLDIDWDCDFRDVIESIMGDEGAMME
PESEAVPMLHDQEGLCNSASTGLSVADGVSFGEPKTDESKGLRLVHLLVAAADASTGANK
SRELTRVILARLKDLVSPGDRTNMERLAAHFTNGLSKLLERDSVLCPQQHRDDVYDQADV
ISAFELLQNMSPYVNFGYLTATQAILEAVKYERRIHIVDYDINEGVQWASLMQALVSRNTG
PSAQHLRITALSRATNGKKSVAAVQETGRRLTAFADSIGQPFSYQHCKLDTNAFSTSSLKLV
RGEAVVINCMLHLPRFSHQTPSSVISFLSEAKTLNPKLVTLVHEEVGLMGNQGFLYRFMDL
LHQFSAIFDSLEAGLSIANPARGFVERVFIGPWVANWLTRITANDAEVESFASWPQWLETN
GFKPLEVSFTNRCQAKLLLSLFNDGFRVEELGQNGLVLGWKSRRLVSASFWASCQTNQ
>AtGRAS25
MQTSQKHHSAAGLHMLYPQVYCSPQFQAKDNKGFSDIPSKENFFTLESSTASGSLPSYDSP
SVSITSGRSPFSPQGSQSCISDLHHSPDNVYGSPLSGVSSLAYDEAGVKSKIRELEVSLLSGD
TKVEEFSGFSPAAGKSWNWDELLALTPQLDLKEVLVEAARAVADGDFATAYGFLDVLEQ
MVSVSGSPIQRLGTYMAEGLRARLEGSGSNIYKSLKCNEPTGRELMSYMSVLYEICPYWK
FAYTTANVEILEAIAGETRVHIIDFQIAQGSQYMFLIQELAKRPGGPPLLRVTGVDDSQSTY
ARGGGLSLVGERLATLAQSCGVPFEFHDAIMSGCKVQREHLGLEPGFAVVVNFPYVLHH
MPDESVSVENHRDRLLHLIKSLSPKLVTLVEQESNTNTSPFLSRFVETLDYYTAMFESIDAA
RPRDDKQRISAEQHCVARDIVNMIACEESERVERHEVLGIWRVRMMMAGFTGWPVSTSA
AFAASEMLKAYDKNYKLGGHEGALYLFWKRRPMATCSVWKPNPN
>AtGRAS26
MKIPASSPQDTTNNNNNTNSTDSNHLSMDEHVMRSMDWDSIMKELELDDDSAPNSLKTG
FTTTTTDSTILPLYAVDSNLPGFPDQIQPSDFESSSDVYPGQNQTTGYGFNSLDSVDNGGFD
FIEDLIRVVDCVESDELQLAQVVLSRLNQRLRSPAGRPLQRAAFYFKEALGSFLTGSNRNPI
RLSSWSEIVQRIRAIKEYSGISPIPLFSHFTANQAILDSLSSQSSSPFVHVVDFEIGFGGQYAS
LMREITEKSVSGGFLRVTAVVAEECAVETRLVKENLTQFAAEMKIRFQIEFVLMKTFEMLSF
KAIRFVEGERTVVLISPAIFRRLSGITDFVNNLRRVSPKVVVFVDSEGWTEIAGSGSFRREFV
SALEFYTMVLESLDAAAPPGDLVKKIVEAFVLRPKISAAVETAADRRHTGEMTWREAFCA
AGMRPIQLSQFADFQAECLLEKAQVRGFHVAKRQGELVLCWHGRALVATSAWRF
>AtGRAS27
MDTLFRLVSLQQQQQSDSIITNQSSLSRTSTTTTGSPQTAYHYNFPQNDVVEECFNFFMDE
EDLSSSSSHHNHHNHNNPNTYYSPFTTPTQYHPATSSTPSSTAAAAALASPYSSSGHHNDP
SAFSIPQTPPSFDFSANAKWADSVLLEAARAFSDKDTARAQQILWTLNELSSPYGDTEQKL
ASYFLQALFNRMTGSGERCYRTMVTAAATEKTCSFESTRKTVLKFQEVSPWATFGHVAAN
GAILEAVDGEAKIHIVDISSTFCTQWPTLLEALATRSDDTPHLRLTTVVVANKFVNDQTASH
RMMKEIGNRMEKFARLMGVPFKFNIIHHVGDLSEFDLNELDVKPDEVLAINCVGAMHGI

ASRGSPRDAVISSFRRLRPRIVTVVEEEADLVGEEEGGFDDEFLRGFGECLRWFRVCFESW
EESFPRTSNERLMLERAAGRAIVDLVACEPSDSTERRETARKWSRRMRNSGFGAVGYSDE
VADDVRALLRRYKEGVWSMVQCPDAAGIFLCWRDQPVVWASAWRPT
>AtGRAS28
MKRSHQETSVEEEAPSMVEKLENGCGGGGDDNMDEFLAVLGYKVRSSDMADVAQKLEQ
LEMVLSNDIASSSNAFNDTVHYNPSDLSGWAQSMLSDLNYYPDLDPNRICDLRPITDDDE
CCSSNSNSNKRIRLGPWCDSVTSESTRSVVLIEETGVRLVQALVACAEAVQLENLSLADAL
VKRVGLLAASQAGAMGKVATYFAEALARRIYRIHPSAAAIDPSFEEILQMNFYDSCPYLKF
AHFTANQAILEAVTTSRVVHVIDLGLNQGMQWPALMQALALRPGGPPSFRLTGVGNPSNR
EGIQELGWKLAQLAQAIGVEFKFNGLTTERLSDLEPDMFETRTESETLVVNSVFELHPVLS
QPGSIEKLLATVKAVKPGLVTVVEQEANHNGDVFLDRFNEALHYYSSLFDSLEDGVVIPSQ
DRVMSEVYLGRQILNLVATEGSDRIERHETLAQWRKRMGSAGFDPVNLGSDAFKQASLLL
ALSGGGDGYRVEENDGSLMLAWQTKPLIAASAWKLAAELRR
>AtGRAS29
MTTKRIDRDLPSSDDPSSAKRRIEFPEETLENDGAAAIKLLSLLLQCAEYVATDHLREASTL
LSEISEICSPFGSSPERVVAYFAQALQTRVISSYLSGACSPLSEKPLTVVQSQKIFSALQTYNS
VSPLIKFSHFTANQAIFQALDGEDSVHIIDLDVMQGLQWPALFHILASRPRKLRSIRITGFGS
SSDLLASTGRRLADFASSLNLPFEFHPIEGIIGNLIDPSQLATRQGEAVVVHWMQHRLYDVT
GNNLETLEILRRLKPNLITVVEQELSYDDGGSFLGRFVEALHYYSALFDALGDGLGEESGE
RFTVEQIVLGTEIRNIVAHGGGRRKRMKWKEELSRVGFRPVSLRGNPATQAGLLLGMLPW
NGYTLVEENGTLRLGWKDLSLLTASAWKSQPFD
>AtGRAS30
MYKQPRQELEAYYFEPNSVEKLRYLPVNNSRKRFCTLEPFPDSPPYNALSTATYDDTCGSC
VTDELNDFKHKIREIETVMMGPDSLDLLVDCTDSFDSTASQEINGWRSTLEAISRRDLRAD
LVSCAKAMSENDLMMAHSMMEKLRQMVSVSGEPIQRLGAYLLEGLVAQLASSGSSIYKA
LNRCPEPASTELLSYMHILYEVCPYFKFGYMSANGAIAEAMKEENRVHIIDFQIGQGSQWV
TLIQAFAARPGGPPRIRITGIDDMTSAYARGGGLSIVGNRLAKLAKQFNVPFEFNSVSVSVS
EVKPKNLGVRPGEALAVNFAFVLHHMPDESVSTENHRDRLLRMVKSLSPKVVTLVEQES
NTNTAAFFPRFMETMNYYAAMFESIDVTLPRDHKQRINVEQHCLARDVVNIIACEGADRV
ERHELLGKWRSRFGMAGFTPYPLSPLVNSTIKSLLRNYSDKYRLEERDGALYLGWMHRD
LVASCAWK
>AtGRAS31
MESGFSGGGGGGSDFYGGGGGRSIPGGPGTVINVGNNNPQTTYRNQIPGIFFDQIGNRVAGG
NGFSGKRTLADFQAAQQHQQQQQQQPFYSQAALNAFLSRSVKPRNYQNFQSPSPMIDLTS
VNDMSLFGGSGSSQRYGLPVPRSQTQQQQSDYGLFGGIRMGIGSGINNYPTLTGVPCIEPV
QNRVHESENMLNSLRELEKQLLDDDDESGGDDDVSVITNSNSDWIQNLVTPNPNPNPVLS
FSPSSSSSSSSPSTASTTTSVCSRQTVMEIATAIAEGKTEIATEILARVSQTPNLERNSEEKLVD
FMVAALRSRIASPVTELYGKEHLISTQLLYELSPCFKLGFEAANLAILDAADNNDGGMMIP
HVIDFDIGEGGQYVNLLRTLSTRRNGKSQSQNSPVVKITAVANNVYGCLVDDGGEERLKA
VGDLLSQLGDRLGISVSFNVVTSLRLGDLNRESLGCDPDETLAVNLAFKLYRVPDESVCTE
NPRDELLRRVKGLKPRVVTLVEQEMNSNTAPFLGRVSESCACYGALLESVESTVPSTNSDR
AKVEEGIGRKLVNAVACEGIDRIERCEVFGKWRMRMSMAGFELMPLSEKIAESMKSRGNR
VHPGFTVKEDNGGVCFGWMGRALTVASAWR
>AtGRAS32

MDALLQVSVDGFRFENGSGSCCKPRNNLESGNNLFPDFHESQNQSSPNDSPPTVCLDNSP
VLKYINDMLMDEEDFVGISRDDLALQAAERSFYEIIQQQSPESDQNTSSSSDQNSGDQDFC
FPSTTTDSSALVSSGESQRKYRHRNDEEDDLENNRRNKQPAIFVSEMEELAVKLEHVLLVC
KTNQEEEEERTVITKQSTPNRAGRAKGSSNKSKTHKTNTVDLRSLLTQCAQAVASFDQRR
ATDKLKEIRAHSSSNGDGTQRLAFYFAEALEARITGNISPPVSNPFPSSTTSMVDILKAYKL
FVHTCPIYVTDYFAANKSIYELAMKATKLHIVDFGVLYGFQWPCLLRALSKRPGGPPMLR
VTGIELPQAGFRPSDRVEETGRRLKRFCDQFNVPFEFNFIAKKWETITLDELMINPGETTVV
NCIHRLQYTPDETVSLDSPRDTVLKLFRDINPDLFVFAEINGMYNSPFFMTRFREALFHYSS
LFDMFDTTIHAEDEYKNRSLLERELLVRDAMSVISCEGAERFARPETYKQWRVRILRAGF
KPATISKQIMKEAKEIVRKRYHRDFVIDSDNNWMLQGWKGRVIYAFSCWKPAEKFTNNNL
NI
>AtGRAS33
MAYMCTDSGNLMAIAQQVIKQKQQQEQQQQQHHQDHQIFGINPLSLNPWPNTSLGFGLS
GSAFPDPFQVTGGGDSNDPGFPFPNLDHHHATTGGGFRLSDFGGGTGGGEFESDEWME
TLISGGDSVADGPDCDTWHDNPDYVIYGPDPFDTYPSRLSVQPSDLNRVIDTSSPLPPPTLW
PPSSPLSIPPLTHESPTKEDPETNDSEDDDFDLEPPLLKAIYDCARISDSDPNEASKTLLQIRE
SVSELGDPTERVAFYFTEALSNRLSPNSPATSSSSSSTEDLILSYKTLNDACPYSKFAHLTAN
QAILEATEKSNKIHIVDFGIVQGIQWPALLQALATRTSGKPTQIRVSGIPAPSLGESPEPSLIAT
GNRLRDFAKVLDLNFDFIPILTPIHLLNGSSFRVDPDEVLAVNFMLQLYKLLDETPTIVDTA
LRLAKSLNPRVVTLGEYEVSLNRVGFANRVKNALQFYSAVFESLEPNLGRDSEERVRVERE
LFGRRISGLIGPEKTGIHRERMEEKEQWRVLMENAGFESVKLSNYAVSQAKILLWNYNYS
NLYSIVESKPGFISLAWNDLPLLTLSSWR
>AtGRAS34
MQIPTLIDSMANKLHKKPPPLLKLTVIASDAEFHPPPLLGISYEELGSKLVNFATTRNVAME
FRIISSSYSDGLSSLIEQLRIDPFVFNEALVVNCHMMLHYIPDEILTSNLRSVFLKELRDLNPT
IVTLIDEDSDFTSTNFISRLRSLYNYMWIPYDTAEMFLTRGSEQRQWYEADISWKIDNVVA
KEGAERVERLEPKSR
>OsGRAS1
MAMDTFPFQWPMDPAASSGLDAGFLPPPAAVAPDDGVGYYDPPAGADVDAAALPEFAAA
FPPCAPDAAAAVLAMRREEEEVAGIRLVHLLMSCAGAIEAGDHALASAQLADSHAALAA
VSAASGIGRVAVHFTTALSRRLFPSPVAPPTTDAEHAFLYHHFYEACPYLKFAHFTANQAIL
EAFHGCDHVHVIDFSLMQGLQWPALIQALALRPGGPPFLRITGIGPPSPTGRDELRDVGLR
LADLARSVRVRFSFRGVAANSLDEVRPWMLQIAPGEAVAFNSVLQLHRLLGDPADQAPID
AVLDCVASVRPKIFTVIEQEADHNKTGFLDRFTEALFYYSAVFDSLDAASASGGAGNAMA
EAYLQREICDIVCGEGAARRERHEPLSRWRDRLTRAGLSAVPLGSNALRQARMLVGLFSG
EGHSVEEADGCLTLGWHGRPLFSASAWEAAGDGGGDNNNNSNSNVSGSSGSDSNNSGSS
NGKSSGARDGSSVCL
>OsGRAS2
MVMDAGVHDVCTMLPGSKRDAHLPLPIYPQIAAANGFATAEEFDPLLFLSPDAVCGGGG
GDYLNIVSAQPISAASTNGASPPRDVSVSASAASSAAAQHDDSEAFSDIVLGYINRMLMA
EDIDEKFEHYPVNADDLLAAEKPFLEILADQSPYSGGSSVESPDGSSAANSCNSLSPCNCSS
SSDGLGAVPQTPVLEFPTAAFSQTPQLYGDLIPTGGMVESGGAWPYDPTEFYQLQTKPVRE
NLPSQSSSFASSNGSSVTFSEGFESLLSPAGVLPDVSLNDFVVQNQQALQFRRGFQEASKFL
PDESKLVIDVDKLYSGDEGSRFLGEVRQEKKLVKVKTETSDVESAGHRGKKHFYGDDLD

AEEGRCSKHSAQGIDTDHLVRDLMDKVLLCNGETCSKGVKELREALQHDVAKHSGGGH
GKGSSHGKGRGKKQPKKEVVDLETLLIHCAQSVATDDRRSATELLKQIRQHAHANGDGD
QRLAHCFANGLEARLAGTGSQIYKNYTITRLPCTDVLKAYQLYLAACPFKKISHYFANQTI
LNAVEKAKKVHIVDYGIYYGFQWPCLIQRLSNRPGGPPKLRITGIDTPQPGFRPAERTEETG
RYLSDYAQTFNVPFEFQAIASRFEAVRMEDLHIEEDEVLIVNCMFKFKNLMDESVVAESPR
NMALKTIRKMNPHVFIHGVVNGSYNAPFFVTRFREALFHYSAIFDMLETNIPKDNEQRLLI
ESALFSREAINVISCEGLERMERPETYKQWQVRNQRVGFKQLPLNQDMMKRAREKVRCY
HKDFIIDEDNRWLLQGWKGRILFALSTWKPDNRSSS

>OsGRAS3

MSFIRRADPSTTYADNLYIHKFGTPNSNFAARRYASDTQLFRYGPEPYNPENSFYNQQASP
MPYMVTADGHSPSSADNSCSDVAKDSPLVSNVSQQNSQSISDNQSSELEVEFDEDDIRMK
LQELEHALLDDSDDILYEISQAGSINDEWADPMKNVILPNSPKESESSISCAGSNNGEPRTP
KQLLFDCAMALSDYNVDEAQAIITDLRQMVSIQGDPSQRIAAYLVEGLAARIVASGKGIYK
ALSCKEPPTLYQLSAMQILFEICPCFRFGFMAANYAILEACKGEDRVHIIDFDINQGSQYITL
IQFLKNNANKPRHLRITGVDDPETVQRTVGGLKVIGQRLEKLAEDCGISFEFRAVGANIGD
VTPAMLDCCPGEALVVNFAFQLHHLPDESVSIMNERDQLLRMVKGLQPKLVTLVEQDAN
TNTAPFQTRFREVYDYYAALFDSLDATLPRESPDRMNVERQCLAREIVNILACEGPDRVER
YEVAGKWRARMTMAGFTPCPFSSNVISGIRSLLKSYCDRYKFEEDHGGLHFGWGEKTLIV
SSAWQ

>OsGRAS4

MGMSPEPCNSISDCSQQQSHHLTLTQQQDSTIICTNQELDYYYRFYDVDEAAFDGNEVEL
VSRFSKVTRMDHMISSPYQPTWSPAQAAVDVVGSSETSRVRKKRFWDVLESCKQKVEAM
EAMDTPATATFRVGAGDGGGGGGGGAGGGGGGADGMRLVQLLVACAEAVACRDRAQA
AALLRELQAGAPVHGTAFQRVASCFVQGLADRLPLAHPPALGPASMAFCIPPSSCAGRDG
ARGEALALAYELCPYLRFAHFVANACMLEAFEGESNVHVVDLGMTLGLDRGHQWRGLL
DGLAARASGKPARVRVTGVGARMDTMRAIGRELEAYAEGLGMYLEFRGINRGLESLHID
DLGVDADEAVAINSVLELHSVVKESRGALNSVLQTIRKLSPRAFVLVEQDAGHNGPFFLG
RFMEALHYYAALFDALDAALPRYDARRARVEQFHFGAEIRNVVGCEGAARVERHERAD
QWRRRMSRAGFQSVPIKMAAKAREWLDENAGGGGYTVAEEKGCLVLGWKGKPVIAAS
CWKC

>OsGRAS5

MEMDKFSSSYQPSWPPAQATVDVVGSVETPARRAFQRVASFFVQELLDRRASAGAGPCEH
GVLHPAVVVREAYAEGLGMYLEFRGINRGLESLHIDDLGVDADEAVAINSVLELHSVVKES
RGALNSVLQTIRKLSPRAFVLVEQDAGHNAPFFLGRFMEALHYYAALFDALDAALPRYD
ARRARVEQFHFGAEIRNVVGCEGAARVERHERADQWRRRMSRAGFQSVPIKMAAKARE
WLDENAGGDGYTVADEKVCLVLGWKGKPVIAASCWKC

>OsGRAS6

MVQDEGSSSSVTSSPLHNFSNMPLHPAAAASPTPPWMVRELRSDERGLCLIHLLLNCAAA
AAAGRLDAANAALEHIASLAAPDGDAMQRVAAAFAEALARRALRAWPGLCRALLLPRA
SPTPAEVAAARRHFLDLCPFLRLAGAAANQSILEAMESEKIVHVIDLGGADATQWLELLHL
LAARPEGPPHLRLTSVHEHKELLTQTAMALTKEAERLDVPFQFNPVVSRLDALDVESLRV
KTGEALAICSSLQLHCLLASDDDAAAVAGGDKERRSPESGLSPSTSRADAFLGALWGLSP
KVMVVAEQEASHNAAGLTERFVEALNYYAALFDCLEVGAARGSVERARVERWLLGEEIK
NIVACDGGERRERHERLERWARRLEGAGFGRVPLSYYALLQARRVAQGLGCDGFKVREE

KGNFFLCWQDRALFSVSAWRGRRFD
>OsGRAS7
MLGSSPARDRGGGDDAEASEQPQPQPQPPLSPRAGGGEARGLVLACADLVHRGDLDGAR
RVAEAVLAAADPRGEAGDRLAHHFARALLALRGGGKGGHGGGGGGVVPSSAAYLAYIKI
APFLRFAHLTANQAILEAAAADAGGAHRRVLHIVDLDAAHGVQWPPLLQAIADRADPAV
GPPPEVRLTGAGTDRDVLLRTGDRLRAFSSSLNLPFRFHPLILPCTAELAADPTAALELHPD
ETLAVNCVLFLHKLGGDGELAAFLRWVKSMNPAVVTIAEREGVLGGDVDDDNVPDELPR
RVAAAMDYYSSVFDALEATVPPASADRLAVEQEILSREIDAAVAAPGAGGGGRARDFDAW
ASAARAAGLAPRPLSAFAASQARLLLRLHYPSEGYKADDDGGRGACFLRWQQRPLMSVS
SWQPQP
>OsGRAS8
MTSDWGDTIQRLNSVTAASSPSLPLLTVVNNTALLARSPTNSSSSTASSSASSSPPISAASSR
QLLSEAAAAIANGNHIVAASLLSALKLSVNPQGDAEQRLVAMMVAALSSCVGTSPSQHLA
DLYIGVGRRRWSEDRERRKERLDS
>OsGRAS9
MRAALFGAERSGVVDHGGGDKEELFWPAGKGGLVVVEPRSVLDCTRSPSPPYSTSTLSSS
LGGGSADSTGVAAVSESSTAAAGATKWGAPGEHGGGGKEEWGGGCELPPIPGALDVGLV
GGEGWDATATLGNAAGPDQSFLNWIIGAAGDLEQPGPPLPVLQQPLIDNAGFGIPAVDTM
GFSLDHPLSGVASDLSSSGAHTATGGGGKASLGFGLFSPEATSLEQPPPSMLFHEGIDTKPP
LLGAQPQFLLNHYQPQPPNPAAALFMPLPPFPEHNHQSPHLQPPLKRHHAIPDDLYLARNQ
QQSSAVAPGLAYSPPLHGPAPFQLHPSPPPIRGAMKSTAAEAAQQQLLDELAAAAKATEAG
NSVGAREILARLNQQLPQLGKPFLRSASYLKEALLLALADSHHGSSGVTSPLDVALKLAA
YKSFSDLSPVLQFTNFTATQALLDEIGGMATSCIHVIDFDLGVGGQWASFLQELAHRRGAG
GMALPLLKLTAFMSTASHHPLELHLTQDNLSQFAAELRIPFEFNAVSLDAFNPAELISSSGD
EVVAVSLPVGCSARAPPLPAILRLVKQLCPKVVVAIDHGGDRADLPFSQHFLNCFQSCVFL
LDSLDAAGIDADSACKIERFLIQPRVEDAVIGRHKAQKAIAWRSVFAATGFKPVQLSNLAE
AQADCLLKRVQVRGFHVEKRGAALTLYWQRGELVSISSWRC
>OsGRAS10
MRAALFGAERSGVVDLGGIGGGNRGLFWPAGKGGLVVVEPRSVLDCTRSPSPRNSTSTLS
SSQGGGGADSTGVAAVSESSAAAAEATKWGAPGEHGGGGGGGGGGGKEDWSSGCELPPI
PGTLDVGLVGGEGWDTMLGNAAAAAAGQDQSFLNWIIGAAGDLEQPGPPLLDNAGFGIP
AVDPLGFSLDHSLSGVASDLSSSGAHTATGGAGGGKASLGFGLFSPEATSLEQPPPPMLFHE
GIDTKPPLLGAQPPGLLNHYHHQPPNPAATFFMPHPSFPEHNHQSPLLQPPPKRHHSMPDD
IYLARNQLPPAAAAAQGLPFSPLHASVPFQLQPSPPPIRGAMKTTAAEAAQQQLLDELAAA
AKATEAGNSVGAREILARLNQQLPPLGKPFLRSASYLREALLLALADSHHGVSSVTTPLDV
ALKLAAYKSFSDLSPVLQFANFTATQALLDEIGGTATSCIHVIDFDLGVGGQWASFLQELA
HRRAAGGVTLPLLKLTAFVSTASHHPLELHLTQDNLSQFAADLGIPFEFNAVSLDAFNPGE
LISSTGDEVVAVSLPVGCSARAPPLPAILRLVKQLSPKIVVAIDHGADRADLSFSQHFLNCFQ
SCVFLLDSLDAAGIDADSACKIERFLIQPRVHDMVLGRHKVHKAIAWRSVFAAAGFKPVP
PSNLAEAQADCLLKRVQVRGFHVEKCGAALTLYWQRGELVSISSWRC
>OsGRAS11
MDPAGAPWCDPRRGYGGYGVGSAALQAAARQQSQQPRSDGAGGAGVTGGVLKRSLGE
MERWQQQRQVAAQQAMYLRSVRQRMDIGAVLGGAASSPAYGISGLSSGFGGISQQQPSS
TMSSLTTASRTVMPGMQQRRRMMAVPTAQNQAVARAPAARPATATELVLLQELEKQLLGD

DEEADAAGSGCGSGITSSDWGDTIQRLNSVTAASSPSLPLPTAVNSTALLARSPTNSSSSTA
SSSASSSPPISAASSRQLLSEAAAAVADGNHTAAASLLSALKLSANPRGDAEQRLVAMMVA
ALSSRVGTGPSQHLADLYSGEHRAACQLLQDVSPCFGLALHGANLAILDAVAGHRAIHLV
DFDVSAAQHVALIKALADRRVPATSLKVTVVADPTSPFTPAMTQSLAATCERLKKLAQQA
GIDFRFRAVSCRAPEIEASKLGCEPGEALAVNLAFTLSRVPDESVSPANPRDELLRRVRALG
PRVVTLVEQELNTNTAPMAARFSDASAHYGAVLESLDATLGRDSADRTRAEAALASKVAN
AVGREGPDRVERCEVFGKWRARFGMAGFRAVAIGEDIGGRVRARLGPALPAFDVKLDNG
RLGVGWMGRVVTVASAWR

>OsGRAS12

MSMQASNRPYRYPDNSQIPYYSRSSMHVGQNGTYHVQQNHEDLYASSDDGSQNGNSKA
QGLQAQYCTLDSSSGNFVYPAHSSTSSHISGSPISQQDSHSEHTSGSPASASCVTEVPGLRF
TTIEEIENAMFGPEPDTVSSDCSLLTDSAFYQDNWREHLGINTGDLKQVIAACGKAVDENS
WYRDLLISELRNMVSISGEPMQRLGAYMLEGLVARLSSTGHALYKSLKCKEPTSFELMSY
MHLLYEICPFFKFGYMSANGAIAEAVKGENFVHIIDFQIAQGSQWATMIQALAARPGGPPY
LRITGIDDSNSAHARGGGLDIVGRRLFNIAQSCGLPFEFNAVPAASHEVMLEHLDIRSGEVI
VVNFAYQLHHTPDESVGIENHRDRILRMVKGLSPRVVTLVEQEANTNTAPFFNRYLETLD
YYTAMFEAIDVACPRDDKKRISTEQHCVARDIVNLIACEGAERVERHEPFGKWRARLSMA
GFRPYPLSALVNNTIKKLLDSYHSYYKLEERDGALYLGWKNRKLVVSSAWR

>OsGRAS13

MEVTMEDVAGDFEFSGCGSTTTTSSASSLDDGTGMCYAWGELSPVADWANFCCSDDDGG
HDLHGLIESMLCDDTLVGVDDDGQAGLHHADMFRDDLYCYGNGSNPSSTTTTNPGSPVF
DDPTQGCPEKGLRLLHLLMAAAEALSGPHKSRELARVILVRLKEMVSHTASANAAASNM
ERLAAHFTDALQGLLDGSHPVGGSGRQAAAAASHHHAGDVLTAFQMLQDMSPYMKFG
HFTANQAILEAVSGDRRVHIVDYDIAEGIQWASLMQAMTSRADGVPAPHLRITAVSRSGGG
GARAVQEAGRRLSAFAASIGQPFSFGQCRLDSDERFRPATVRMVKGEALVANCVLHQAAA
TTTIRRPTGSVASFLSGMAALGAKLVTVVEEEGEAEKDDDGDSAGDAAAGGFVRQFMEE
LHRYSAVWDSLEAGFPTQSRVRGLVERVILAPNIAGAVSRAYRGVDGEGRCGWGQWMRG
SGFTAVPLSCFNHSQARLLLGLFNDGYTVEETGPNKIVLGWKARRLMSASVWAPPPLPVP
SSPPEGVCQPVVGMAPVATGGFARTEFDYIDSFLVEPAYALV

>OsGRAS14

MSYHEEERHGGNGLDWFEESMSSLLAADVDLAGGGGDAGGGGYAWWWAASPAAQQD
DIGSVVAQTLSPPSTAAPAAASPSIASPAASSPSDVPSSSSKKRKSPAHRAPGHTGGKKGGG
GKGGGGGSDRDMRWAEQLLNPCAVAVEAGNLSRVQHLFYVLGELESFSGDANHRLAAH
GLRALARWLPAAVGPAAAAAVRVPPCSERPTTAFAAAEPRLFRASLIRFHEVSPWFALPNA
LANAAIAQASTCGAAGATPRPLHVVDLGVSHGVQWPTLLESLTRQPGGRAPPSVRLTVVG
PGATATSPVAPFSASPPGYDFSPHLLRYAKSINLDLRISRAATLDDAVPGDDGEALVVCLQF
RLGHAAAEERREVLRKARGLNPELVVLSELDSGVGVVGGDGGSAAGEFAARLELLWRFL
ESTSAAFKGKDVEERRLLEAEAGAILAAADVAAAGEGREGWRERMAAAGFEEAPFGAEA
VESARSLLRKYDSGWEMSAPSPAAAAVALRWKGQPVSFCSLWRPAA

>OsGRAS15

MDTLFRLVSLHHHHHHQHAASPSPPDQPHKSYPSSRGSTSSPSSHHTHNHTYYHHSHSHY
NNNSNTNYYYQGGGGGGGGYYYAEEQQPAAYLEECGNGHQFYMDEDFSSSSSSRQFHS
GTGAPSSAPVPPPPSATTSSAGGHGLFEAADFSFPQVDISLDFGGSPAVPSSSGAGAGAGAA
PSSSGRWAAQLLMECARAVAGRDSQRVQQLMWMLNELASPYGDVDQKLASYFLQGLFA

RLTTSGPRTLRTLATASDRNASFDSTRRTALKFQELSPWTPFGHVAANGAILESFLEAAAAG
AAASSSSSSSSSTPPTRLHILDLSNTFCTQWPTLLEALATRSSDDTPHLSITTVVPTAAPSAA
AQRVMREIGQRLEKFARLMGVPFSFRAVHHSGDLADLDLAALDLREGGATAALAVNCVN
ALRGVARGRDAFVASLRRLEPRVVTVVEEEADLAAPEADASSEADTDAAFVKVFGEGLR
FFSAYMDSLEESFPKTSNERLSLERAVGRAIVDLVSCPASQSAERRETAASWARRMRSAGF
SPAAFSEDVADDVRSLLRRYKEGWSMRDAGGATDDAAGAAAAGAFLAWKEQPVVWAS
AWKP

>OsGRAS16

MGAGRRRRQKMMPPPLSNFDARNGVPTPGQAEQETMELVRMLTACADSVSAGNHEAAI
YYLARLCEMASLAGPMPIHRVAAYFIEVLTLRVVRMWPHMFNISPPRELTNDAFSGDDDA
MALRILNTITPILLLGKHS

>OsGRAS17

MSASQNLHRLPEFCVIPATACSREPGLPRIVIRSNMGSDDNKRFDDFHVITNGPGSDAYSNE
ESINLDSYIAPSTSFCSKIYNPQLYVEKGNGTTDWCQTSGGDYPEKSSINSDITLSYIDKILM
QEDIDDRGNEDTALQAMEEPFYELLGEKYPAFPQQQPLCVCDHLQNLSANTDKSNGHAC
NTWSVTRMTNISSSMNSNGNFQGFQFPWSLSSITRETEQFTHHSNRMVVGLKVDGLSISE
KPSQDNCSLQIDAHYMRKHPLFEVHDRKSYPCIEDLDLLEGRSNKQYAIYYDEPIRDEMF
DNVLLCSDHKPLDEGVSLSRAMTNNSSKSSQIGQGKTSARRKTTGKRIQKRDVVDLRTLL
INCAQAVSVSNHSLASDILKIIRHHASPTGDDSQRLALCLAYCLDVRLTGTGSQIYHKFITK
RRNVKDILKVHIIDFGICFGFQWPSLFEELAKIEDGPPKLRITGIELPESGFRPYARSNNIGLR
LADYAKTFNIPFEYQHISSNKWEALSPEDFNIEKDEVLIVNCIYRIKDLGDETISINSARSRV
LNTIRMMKPKVFVQGVLNGSYGVPFFLTRFKEVMYHYNSLFDMLDKNIPRDNETRMIIER
DIYQYIMLNVIACEGPERIERPESYKKWKVRNLKAGLVQLPLNPAIVRETQDMSSDKASIR
LCLVCYGNILSWPHPPEAEGTYWSGVESDRVYRDCSLNNRGPLCNGIPFLNGMIPVSDDF
YHVSGDTREISCDTYQVLDDFYHVSGDTYEISDDTYRISGDSY

>OsGRAS18

MGLDNNFGELSGMFCGLSYDGYTDHGSQSDYFRFADPQPAIVPQMDAGPSSAASSTASRA
AVSSGTDNPEDWEFISDESLNYISRMLMEEDIDEKVSMYQEESAALRAAAKPFYDILGHKF
PPSPDRQLVAWPLDSPSESSTSSYPHSLASSVTSSNISGAVDSSQRRYVGHSEYRSLSGHSSQ
PPVGPSSDVRNAMETLEDPLISNGRIPEYLFESFPTWDFRRGVDEAQKFLPGSDKVVIDLE
AGGVAKRQEAGKAISLNVSKAEVLKVKKNRQSEDLDVMEGRNSKQSAFCSDEPDWIEMF
DVLLRQTEKKATDLRKMMRFEASKNSQVAQPKGPSGTRSRGRKPTKKDVVDLRTLLIHC
AQAVAADDRRTANELLKQIRQHAKPNGDGSQRLAYCFADGLEARLAGTGSQLYHKLVAK
RTTASDMLKAYHLYLAACPFKRLSHFLSNQTILSLTKNASKVHIIDFGIYFGFQWPCLIRRL
FKREGGPPKLRITGIDVPQPGFRPTERIEETGQRLAEYAEKIGVPFEYQGIASKWETICVEDL
NIKKDEVVIVNCLYRFRNLIDETVAIDSPRNRVLNTIRQVNPAIFIHGIVNGSYSVPFFITRFR
EALFHFSALFDMLETTVPRDDAQRALIERDLFGREALNVIACEGSDRVERPETYKQWQVR
NLRAGFVQSPLNQDIVLKAKDKVKDIYHKDFVIDEDSEWLLQGWKGRIIYAISTWKPNNN

>OsGRAS19

MKREYQEAGGSSGGGSSADMGSCKDKVMAGAAGEEEDVDELLAALGYKVRSSDMADV
AQKLEQLEMAMGMGGVSAPGAADDGFVSHLATDTVHYNPSDLSSWVESMLSELNAPLP
PIPPAPPAARHASTSSTVTGGGGSGFFELPAAADSSSSTYALRPISLPVVATADPSAADSARD
TKRMRTGGGSTSSSSSSSSSLGGGASRGSVVEAAPPATQGAAAANAPAVPVVVVDTQEAG
IRLVHALLACAEAVQQENFAAAEALVKQIPTLAASQGGAMRKVAAYFGEALARRVYRFRP

ADSTLLDAAFADLLHAHFYESCPYLKFAHFTANQAILEAFAGCHRVHVVDFGIKQGMQWP
ALLQALALRPGGPPSFRLTGVGPPQPDETDALQQVGWKLAQFAHTIRVDFQYRGLVAATL
ADLEPFMLQPEGEADANEEPEVIAVNSVFELHRLLAQPGALEKVLGTVHAVRPRIVTVVE
QEANHNSGSFLDRFTESLHYYSTMFDSLEGGSSGQAELSPPAAGGGGGTDQVMSEVYLG
RQICNVVACEGAERTERHETLGQWRNRLGRAGFEPVHLGSNAYKQASTLLALFAGGDGY
RVEEKEGCLTLGWHTRPLIATSAWRVAAA
>OsGRAS20
MAYMCADSGNLMAIAQQVIQQQQQQQQQQQRHHHHHHLPPPPPPQSMAPHHHQQKHH
HHHQQMPAMPQAPPSSHGQIPGQLAYGGGAAWPAGEHFFADAFGASAGDAVFSDLAAA
ADFDSDGWMESLIGDAPFQDSDLERLIFTTPPPPVPSPPPTHAAATATATAATAAPRPEAAPA
LLPQPAAATPVACSSPSPSSADASCSAPILQSLLSCSRAAATDPGLAAAELASVRAAATDAG
DPSERLAFYFADALSRRLACGTGAPPSAEPDARFASDELTLCYKTLNDACPYSKFAHLTAN
QAILEATGAATKIHIVDFGIVQGIQWAALLQALATRPEGKPTRIRITGVPSPLLGPQPAASLA
ATNTRLRDFAKLLGVDFEFVPLLRPVHELNKSDFLVEPDEAVAVNFMLQLYHLLGDSDELV
RRVLRLAKSLSPAVVTLGEYEVSLNRAGFVDRFANALSYYRSLFESLDVAMTRDSPERVRV
ERWMFGERIQRAVGPEEGADRTERMAGSSEWQTLMEWCGFEPVPLSNYARSQADLLLW
NYDSKYKYSLVELPPAFLSLAWEKRPLLTVSAWR
>OsGRAS21
MNLKLSLAIDGGGGGDAAAAVAKKSKVVGGGAVVVDGVGSSAICGGDRGSRVRDRMVK
KAEEFDHENGMAATSSDGGGGGGGGMELVRLLLSAVAAGEAGDARAAAAALREVDRRA
SCRGGGDPAQRVAACYAAALAPRLAAGLRPARSSPAAPAAARAEQFLAYTMFYQASPFYQ
FAHFTANQAIVEAFESGGRRRLHVVDFDVSYGFQWPSLIQSLSDAAAAATSSSSHDDDDN
GGGCGDGPVSLRITGFGASADELRETEARLRRFAAGCPNLRFEFEGILNNGSNTRHDCTRI
DDDATVVVNLVFPASSREACAATRMAYINSLNPSMVFLIEKHDGGGGLTGGDNTTTGRSA
SLLPRFAANLRYFAAVFDSLHECLPADSAERLAIERDHLGREIADAVASLDHQHRRRHGGG
GGGGDHAAASWNWKAAMEGAGLDGVKLSSRTVSQAKLLLKMKSGCGGGGFRVVEGD
GGMAMSLAWRDMALATATLWRRRRRRRRCR
>OsGRAS22
MHYLRYYDAAFDAVDAAGLLETRPARAKVEEMFAREIRNAVAFEGAERFERHESFAGRRR
RMEDGGGLQWGSKAEEKCLLPSSSLARRPRRQPSPPVSLRPDGSLPPAVAAAPLVLPLPRA
SAAEPPRCAPMPPTAAPLVLPPPLPTEKERI
>OsGRAS23
MRAAPFSADGNGAAELAGSIAALLWPEDKGGGGGGGGGSLLVEPRSVLDCRGSPSPPNST
STLSSSHGSGAADSISTGVAAVSESSAAAAEATRWAAPGEHGGGGGGELPPIPGALDVGFV
AEESWDAMLGDAAAAAGQEQTFLNWIMAAPGDMEPQAPGLSQQQLLANAAGFGFPLQ
HHPGGVSSPAALASDLSSSGGRSLTSSSGSNSKATSAFGLLSPEAALQPPPATTAPFHNGAD
MKPPLLGLPSPTLLLNQHQPTPASTLFMPFPSFSDHQQQPLLQPPPKRHHSVPDNLFLLHN
QPQPPPPAPAQCLPFPTLHSAVPFQLQPSMQHPRNAMKSTAAAAAAQQQHLLDELAAAAK
ATEVGNSIGAREILARLNQQLPPIGKPFLRSASYLKDALLLALADGHHAATRLTSPLDVAL
KLTAYKSFSDLSPVLQFANFTVTQALLDEIASTTASCIRVIDFDLGVGGQWASFLQELAHRC
GSGGVSLPMLKLTAFVSAASHHPLELHLTQDNLSQFAADLGIPFEFNAINLDAFDPMELIAP
TADEVVAVSLPVGCSARTPLPAMLQLVKQLAPKIVVAIDYGSDRSDLPFSQHFLNCLQSCL
CLLESLDAAGTDADAVSKIERFLIQPRVEDAVLGRRRADKAIAWRTVLTSAGFAPQPLSNL
AEAQADCLLKRVQVRGFHVEKRGAGLALYWQRGELVSVSAWRC

>OsGRAS24
MEPGAPWRDPRQGYLYGVGSAVQMPMQQRSDAAAAGGVLKRSLGDMERWQQHQHQQ
RQIAMQQQLYLRTVRQRTAAASAAVSPLTSADIAAVLGGPPSQPLVLSGSSMGGAFGSPSS
TLSSITTASRAVAMPLMQPQLQRQQQVTYMASSPQVQAFGTARALPPAPATSDLSILQELE
KQLLGDDDEVEAAMSGTGSAVTGSEWEEQLNSITAAPSPPLTAATTPNNNNNAVGMTRSP
SNSSTSTASSSASCSPPTSATTSRQLLSEAAAAIADGHNETAATHLTALKRAANSRGDVEQR
LVAMMVAALSSRIGQTASVPDICGGETRAGSQLLHDISPCFRLALHAANVAIVDAVGDHRA
IHLVDFDVSAPQHADLIRCLAARRLPGTSLKVTAVTDPASPFTQSVTATLHLQKLAERAGID
YRFKMVSCRAGEIEASKLGCEAGEALAVNLAFALSHVPDESVSPANPRDEILRRVRALGPQ
VVALVEQELNSNTAPLTTRFTDACAHYGAILESLDATIPRESAERARAEAALGGRAANAVA
REGADRLERCEVFGKWRSRFGMAGFRPVALGPGIADQVLARQGPVAAGFAVKAENGVLR
LGWMGRVVTVASAWR
>OsGRAS25
MLDSGSYDDVDYGDLFSIPNPPAPHLLNFPLQFFPSNGFISSADDSHRSPAGMFGSTPSPTS
TTTELENSEDLSESADDAVLAYINQFLLEDEEDESCPGTITSVEDSALLAVEKPFVDILTASQ
EACQENSWIDSCCDFTGNGGLLDTFTTTHAACQPAPCEFEKEKGECAVHKGRKNPHDDC
LLFEEESRRSKQLAVSEEETVREMFDKVLLCNGECELRAPLPAEARNCGVYVKGSGNKRG
RKKGKSGASAEDDAVDLTTLLIHCAQAAAIDDHRNSNELLKQIRQRSSAYGDAGQRLAHC
FANALEARLAGTGSNIYRSLAAKRTSVYDILNAFKLYVTACPFKKISNFFSIEAILNASKGM
TRLHIVDYGIQYGFQWPIFFQRISKRPGGPPSVRITGVDLPQPGFRPAQLIEATGRRLHDYA
RMFNVPFEYHAIAAKWDTIRVEDLKIDKDKDELLVVNCLFRMRNMMDEMVTDDSPRMQ
VLKTIRKMNPNLFIHGVVNGTYNAPFFVTRFKEALFYYSSLFDMLETTASRVDENRLLIER
DLFGREALNVVACEGTERVERPETYKQWQVRNIRAGFKQLPLNQETVKKARYKVKKSYH
RDFLVDEDNKWMLQGWKGRIIFALSAWEPN
>OsGRAS26
MAVEIPRLALGGGGGGAGGERLPAAGEDSAPAATNAGKRPVVGLGFGSSLAAMAAAAA
AGIQPDAFALGGPAEYPAGDGERDVLMVSFLRSIAAFLADGTCQMQVNDGLSCVVDLAG
GDADGGGVGEGRSAQRLASAFAEALALRFILPCDGVCRSLHLTRAPPPPAVSAARQGFRA
MCPFVRLAAAAANLSIAEVMEAERAVVHVVDLGGGVDANQWVELVRLVAARPGGPPGL
LRLTVVNESEDFLSAVAAYVAAEAQRLDLSLQFHPVLSSIEELSATATGSIGSRLVVIPGQPL
AVVANLQIHRLLAFPDYVDGVASRRPAAEQSGSSQHTMTTATKTKADALLRAIRDLNPKL
VVLTENEADHNVAELGARVWNALNYYAALFDALEASSTPPAAVPPHERACVERWVLGEEI
KDIVVREGTGRRERHETLGRWAERMVAAGFSPVTAARALASTETLAQQMVAAGGGGAG
AGVLRAAHGGGCFPVICWCDVPVFSVSTWTARRVLVPAPPLWPPAAAGGAGPSGSGYGG
DGPSTASSSAAMWWVG
>OsGRAS27
MVAQAATAAATTTAAATTAAVPMTNFQLFGSMVPVPVASMATATAPAAVAAADNGGHGSS
SASQNASGSGEGQGGSMSLSLQLRPLGSTPTAAVAVSVPPMAAAPMMAGPAAAAPAPAPP
LATMAVAQNASLAAVASALAAHRRNQATHRSAALHGHLRRCAEALAASRPADADAELAS
IARMASSDGDAVQRVAAAFAEAMARVVIRPWRGVSAALFPSDAGAAGDALTAWEAEFAR
QSFLNLCPLLHLAAVAVNEIILETTRNDKFIHIVDLGGIHHAHWVELLQGLATRRAAVRPCL
RLTIVHEHKHFLGQAAQVLAAESDRHGVPLDLHIVESSVEALKLDALGVRSDHAVVIVST
LQLHRLVGAGILSTTAPPSPAAAAAASMITSPLPPANMSSKVDRLLRGFHLLSPRAIILTENE
ANHFVPSFTDRFASALPYYEQLFAAMEEAGAATVERKAAERYLLREEIKDVIACDHDGPR

WARHETLGRWVVRMGAAGFALAPAITVVTAAGRVRAVAARLPGGGDERRYGVTEGGGW
LILNREEKPMFCVSAWRRQ
>OsGRAS28
MQDSLGLMQFLDHHQYLYSSSSSNLPLQQPLLSHHHRFLEANEGCAGEDDSPEFVEPPAA
AAAAGTFEQRPELGACKEVYSEEGGAAEERTGVAMAGADVEQVAVEDEEEAHGVRMIA
LLMECAAAMSVGNLAGANGALLELSQMASPYAASCGERLVAYFARAMAARLVGSWVGV
VAPMAPPPSCGAINAAFRALYNVAPFARLAYLACNQAILEAFHGKRLVHIVDLDVVPGGA
LQWLSLLPALAARPGGPPVIRVTGFGMSASVLHDTGNQLAGLARKLCMFFEFYAVAKRPG
DADAVADMPGRRPGEAVAVHWLRHAMYDAAGDDGASMRLVRWLEPAAVTLVEQERAH
GGGGGHGRFLDRFVSALHHYSAVFDAMGASRPDGEDASRHLAEHGVLGREIANVLAVG
GPARSSGREGPGSWREVLARHGFAHAGGGGGGRAQLVAAACPGGLGYTVAGDHDGTVR
LGWKGTPLYAVSAWTWCSPPHARA
>OsGRAS29
MDMEHELRGGGVGAAAHGHGSICFSGGPVLVDGRRIQQLLLHCAAALESNDVTLAQQA
MWVLNNIASSQGDPSQRLTSWLLRALVARACRLCAAAPAGAAVEFLERGRAPPWGRAMS
VTELADYVDLTPWHRFGFTASNAAILRAVAGASAVHVVDLSVTHCMQWPTLIDVLSKRP
GGAPAIRITVPSVRPAVPPLLAVSSSELGARLAIFAKSKGVQLEFNVVESATTSPKKTSTTL
CQELASVLSDPPSLGLRDGEAVVVNCQSWLRHVAPDTRDLFLDTVRALNPCLLTVTDEDA
DLGSPSLASRMAGCFDFHWILLDALDMSAPKDSPRRLEQEAAVGRKIESVIGEEDGAERSE
PGARLAERMSRKGFAGVVFDEEAAAEVRRLLSEHATGWGVKREDDMLVLTWKGHAAVF
TGAWTPN
>OsGRAS30
MAQFGGFGGWSAMDVAAAAAAALGNVSGAVYHADPAAAVYASLVPGMAVVPGRAPPS
AVQIEAARRWKELEKMALRSVNLMVTCAGAIQAGDYAAAAGSLSDAREIFAKMPTTRTG
IGRVLTHFADALAERLFPAFPQSAPPPPPPRGEQRELFRGFYEAGPYLKFAHLAANQAILEA
FEGCNSVHVIDFALTDGIQWPSLIQALAVRPGGPPFLRITGIGPHAAGNRDELRDVGLRLAE
FARSCSVPFAFRGIAADQLDGLRPWMFQVAPGEAVAINSVLQLHRLLVDQDAAAAASFPA
PIDGVLDWVASMNPRVFTVVEQEADHNKSSLLERFTNSLFYYASMFDSLEAISRHGGGDG
AGNPLAEAYLQGEIADIVSREGSSRVERHEQMPRWVERLRRGGMTQLPLGATGLWQAAM
QLREFSGAGFGVQENGGFLTLTWHSQRLYSASAWRATAGKKMTMMASGAADAMEESQN
SNTNGGGGGSSGGGHGALNQIMQ
>OsGRAS31
MSSSSCHPHNPTTLPLPEPDSSKSPEPTSVLYNRSSPSTSLGSCSSKPPEDPPPPIAADDDCD
WDAVVDMHMHMLAPAPAPDSSFLRWIMDTGYADADTFPDHPSFDSDLLQLPMPMPSDHP
PQPLVDDLLDAARLLDAGDSTSAREILARLNHRLPSLPSPPGHAHPPLLRAAALLRDALLP
PTALPVSSTPLDVPLKLAAHKALADASPTVQFTTFTSTQAFLDALGSARRLHLLDFDVGFG
AHWPPLMQELAHHWRRAAGPPPNLKVTALVSPGSSHPLELHLTNESLTRFAAELGIPFEFT
ALVFDPLSSASPPLGLSAAPDEAVAVHLTAGSGAFSPAPAHLRVVKELRPAVVVCVDHGCE
RGALNLLQSCAALLESLDAAGASPDVVSKVEQFVLRPRVERLAVGGGDKLPPPLQSMLAS
AGFAALQVSNAAEAQAECLLRRTASHGFHVEKRQAALALWWQRSELVSVSAWRC
>OsGRAS32
MLAGCSFSSSRHQMSTAQRFDILPCGFSKRGSRGDGAAPRVAGDARSGATTCSFRTHPAPP
VTQSVSWGAKPEPGGNGNGAHRAVKRAHDEDAVEEYGPIVRAKRTRMGGDGDEVWFH
QSIAGTMQATAAGEGEEAEEEKVFLVPSAAAFPHGMAAAGPSLAAAKKEEYSKSPSDSSS

SSGTDGGSSAMMPPPQPPEFDARNGVPAPGQAEREALELVRALTACADSLSAGNHEAANY
YLARLGEMASPAGPTPMHRVAAYFTEALALRVVRMWPHMFDIGPPRELTDDAFGGGDDD
AMALRILNAITPIPRFLHFTLNERLLREFEGHERVHVIDFDIKQGLQWPGLLQSLAARAVPP
AHVRITGVGESRQELQETGARLARVAAALGLAFEFHAVVDRLEDVRLWMLHVKRGECVA
VNCVLAMHRLLRDDAALTDFLGLARSTGATILLLGEHEGGGLNSGRWEARFARALRYYA
AAFDAVDAAGLPEASPARAKAEEMFAREIRNAVAFEGPERFERHESFAGWRRRMEDGGGF
KNAGIGEREAMQGRMIARMFGPDKYTVQAHGGGGSGGGEALTLRWLDQPLYTVTAWTP
AGDGAGGSTVSASTTASHSQQS

>OsGRAS33

MALTKEAERLDVPFQFNPVVSRLDALDVESLRGLSLKVMVVTEQEVSHNAAGLTERFVE
ALNYYAALFDCLEVGGARGSVERTRVERWLLGEEIKNIVACDGGERRERHERLEGAGFGR
VPLSYYALLQARRVAQGLGCDGFKVREEKGNFFLCWQDRALFSVSAWRGRRFAALLPLP
PSTPLPCSRVIATLLSSPPPPCSPLHPYCPERRERMKGEKKGKRE

>OsGRAS34

MHESDELLLLVAFLTLGISLSPSCPLLALCEGWKQGISACCGCMTQMHMSLLVGNDGVQV
EVMIDCLIWGLRHSIQGCPMPCPHIHSQLVELSPSPPLFAIFHLFANVLYQDALDYMQCETL
TQLDQVWGVCLFLLQGSYLEAIINEDPTKGQNMRWLETWEAAIASPDVGRARSPSLLNPP
SSDAPSPPPPSPPPPPPSPAAHRRCSSSGGGGGDDDGYGGGDGMLRSLHSSSSSDTDNNSG
GCKNNGGGGGEAAAAVEGGGDQRAVAAAAPSTRDLLLACADLLQRGDLPAARRAAEIV
LAAAASPRGDAADRLAYHFARALALRVDAKAGHGHVVVGGGAARPASSGAYLAFNQIA
PFLRFAHLTANQAILEAVDGARRVHILDLDAVHGVQWPPLLQAIAERADPALGPPEVRVTG
AGADRDTLLRTGNRLRAFARSIHLPFHFTPLLLSCATTAPHHVAGTSTGAAAAASTAAAAT
GLEFHPDETLAVNCVMFLHNLAGHDELAAFLKWVKAMSPAVVTIAEREAGGGGGGGDHI
DDLPRRVGVAMDHYSAVFEALEATVPPGSRERLAVEQEVLGREIEAAVGPSGGRWWRGIE
RWGGAARAAGFAARPLSAFAVSQARLLLRLHYPSEGYLVQEARGACFLGWQTRPLLSVS
AWQPSSS

>OsGRAS35

MRSPPPLHADSTPERRRGEEMCEMGRVEFLKAIKDVAPKATSCGTRAPARLCLRRGVQPY
VHQRQSTCRRCTPAAALLADLSGSGKKRREEKRRREEKKKKEKEKEKCRRQDAAEMWV
PPYTMSAKSGKIGSIVPRDPFCTHRRGGRRGGQGEEGEAHCVRRDGFAGGPTPMPRVVAY
FAKALALRVVRMWPHMFEISAPWELTDDAFNSDDDAMALRILNTITRAKTETSPGRAKAE
EMFVRERMCNGTVAAAGVRLPERRLW

>OsGRAS36

MDLHQLLKYRLTGANVVYEIPTENNLQNSPWQANPLKYEFSDSPYTPLSSQFECDNLSAL
TNTPDNQSSTETISAQPISPLEADSSYRQAGILLQENIQVGADPLYATSRHNMQHALREIET
VLMAPDTDDATTSTKHEFEEIKPAQLVRQRSRTWSHESRQPLPGVGRSQFASGGYPTASYE
FRPEKRQRELREDPQIIVKQLLTRCAEALSEDRTEEFHKLVQEARGVVSINGEPIQRLGAYL
LEGLVARHGNSGTNIYRALKCREPESKELLSYMRILYNICPYFKFGYMAANGAIAEALRTE
NNIHIIDFQIAQGTQWITLIQALAARPGGPPRVRITGIDDPVSEYARGEGLDIVGKMLKSMS
EEFKIPLEFTPLSVYATQVTKEMLEIRPGEALSVNFTLQLHHTPDESVDVNNPRDGLLRMV
KGLSPKVTTLVEQESHTNTTPFLMRFGETMEYYSAMFESIDANLPRDNKERISVEQHCLA
KDIVNIIACEGKDRVERHELLGKWKSRLTMAGFRPYPLSSYVNSVIRKLLACYSDKYTLDE
KDGAMLLGWRSRKLISASAWH

>OsGRAS37

MLQGVLSRAPGADAAAMKAKRAADDEEEGGERERARGKRLAAEGKQGLVVVSTGEEE
EAAAETRGLRLLSLLLRCAEAVAMDQLPEARDLLPEIAELASPFGSSPERVAAYFGDALCA
RVLSSYLGAYSPLALRPLAAAQSRRISGAFQAYNALSPLVKFSHFTANQAIFQALDGEDRV
HVIDLDIMQGLQWPGLFHILASRPTKPRSLRITGLGASLDVLEATGRRLADFAASLGLPFEF
RPIEGKIGHVADAAALLGPRHHGEATVVHWMHHCLYDVTGSDAGTVRLLKSLRPKLITIV
EQDLGHSGDFLGRFVEALHYYSALFDALGDGAGAAEEEAAERHAVERQLLGAEIRNIVAV
GGPKRTGEVRVERWGDELRRAGFRPVTLAGSPAAQARLLLGMYPWKGYTLVEEDGCLK
LGWKDLSLLTASSWEPTDGDADADVAVAGDTHHESHDS
>OsGRAS38
MADTPTSRMIHPFSNIPSQNLKQFQYSDNPQHPCHPYRAPSDTHVVPHHYGLKSHSPDAG
YESQATPNKYTLDSSEGAGCMRHDSPSSQSFTTRSGSPLSQEDSHSDSTDGSPVGASCVTE
DPNDLKQKLKDLEAVMLGPDSEIVNSLENSVANQLSLEPEKWVRMMGIPRGNLKELLIAC
ARAVEEKNSFAIDMMIPELRKIVSVSGEPLERLGAYMVEGLVARLASSGISIYKALKCKEPK
SSDLLSYMHFLYEACPYFKFGYMSANGAIAEAVKGEDRIHIIDFHISQGAQWISLLQALAA
RPGGPPTVRITGIDDSVSAYARGGGLELVGRRLSHIASLCKVPFEFHPLAISGSKVEAAHLG
VIPGEALAVNFTLELHHIPDESVSTANHRDRLLRMVKSLSPKVLTLVEMESNTNTAPFPQRF
AETLDYYTAIFESIDLTLPRDDRERINMEQHCLAREIVNLIACEGEERAERYEPFGKWKARL
TMAGFRPSPLSSLVNATIRTLLQSYSDNYKLAERDGALYLGWKSRPLVVSSAWH
>OsGRAS39
MDTLFRLVSLQAASEQQQQQQQSASYNSRSTTSSGSRSSSHQTNASYSYYHHSSNSGGGG
GGGGGYYYGGQQPPPSQYYYLEPYQEECGNAPHHQLYMDEDFSSSSSSRHFHHGARVQQ
QQPPASSTPTGTAPTPPLSTSSTAAGAGHGLFEAADLSFPPDLNLDFSSPASSSGGGTASSGA
VGGGGGGRWASQLLLECARSVAARDSQRVQQLMWMLNELASPYGDVEQKLASYFLQGL
FARLTASGPRTLRTLAAASDRNTSFDSTRRTALRFQELSPWSSFGHVAANGAILESFLEVAA
AASSETQRFHILDLSNTFCTQWPTLLEALATRSADETPHLSITTVVSAAPSAPTAAVQRVMR
EIGQRMEKFARLMGVPFRFRAVHHSGDLAELDLDALDLREGGATTALAVNCVNSLRGVV
PGRARRRDAFAASLRRLDPRVVTVVEEEADLVASDPDASSATEEGGDTEAAFLKVFGEGL
RFFSAYMDSLEESFPKTSNERLALERGAGRAIVDLVSCPASESMERRETAASWARRMRSA
GFSPVAFSEDVADDVRSLLRRYREGWSMREAGTDDSAAGAGVFLAWKEQPLVWASAWR
P
>OsGRAS40
MMQFTHTAPPPPPLHPNGHGLGLGLYLDVGATRGGGGARPWSSSSSTTTLGGSGYFPSSA
AASKISLGNLNSTGCMEQLLVHCANAIEANDATLTQQILWVLNNIAPADGDSNQRLTAAFL
CALVSRASRTGACKAVTAAVADAVESAALHVHRFTAVELASFIDLTPWHRFGYTAANAAIV
EAVEGFPVVHIVDLSTTHCMQIPTLIDMLAGRAEGPPILRLTVADVAPSAPPPALDMPYEEL
GAKLVNFARSRNMSMDFRVVPTSPADALTSLVDQLRVQQLVSDGGEALVVNCHMLLHTV
PDETAGSVSLTTAQPPVSLRTMLLKSLRALDPTLVVVVDEDADFTAGDVVGRLRAAFNFL
WIPYDAVDTFLPKGSEQRRWYEAEVGWKVENVLAQEGVERVERQEDRTRWGQRMRAA
GFRAAAFGEEAAGEVKAMLNDHAAGWGMKREDDDLVLTWKGHNVVFASAWAPS
>OsGRAS41
MSARASNHAYICSDDSQMPYYNNSVPSGGNGRFYITQNHQDAHYASSDDGSQKIGSSPQA
FEAPYCTLESSSANGAHPAHSSASSHSISPISGSPLSHHDSHSDHTYNSPPSASCVTEITDLQI
KLRELENAILGPELDIAYDSPESALQPNIMATPENWRQLLGINTGDLKQVIIACGKAVAEND
VRLTELLISELGQMVSVSGDPLQRLGAYMLEGLVARLSSSGSKIYKSLKCKEPTSSELMSY

MHLLYEICPFFKFGYMSANGAIAEAIKGENFVHIIDFQIAQGSQWMTLIQALAARPGGPPF
LRITGIDDSNSAYARGGGLDIVGMRLYKVAQSFGLPFEFNAVPAASHEVYLEHLDIRVGEVI
VVNFAYQLHHTPDESVSTENHRDRILRMVKSLSPRLVTLVEQESNTNTRPFFPRYLETLDY
YTAMFESIDVALPRDDKRRMSAEQHCVARDIVNLIACEGAERVERHEVFGKWKARLTMA
GFRPYPLSSVVNSTIKTLLHTYNSFYRLEERDGVLYLGWKNRVLVVSSAWC
>OsGRAS42
MAAAPAPKLPPFPAGGFVPALKPKAEAANDEAAAAVEQLAEAAKLAEAGDAFGAREILA
RLNYRLPAAPTAGTPLLRSAFYFKEALRLTLSPTGDAPAPSASTPYDVVVKLGAYKAFSEV
SPVLQFAHLTCVQAVLDELGGAGCIHVLDFDIGMGEQWASLMQELAQLRPAAALKVTAL
VSPASHHPLELQLIHENLSGFAAELGVFFHFTVFNIDTLDPAELLANATAGDAVAVHLPVGP
AHAAATPAVLRLVKRLGAKVVVSVDRGCDRSDLPFAAHLFHSFHSAVYLLESIDAVGTDP
DTASKIERYLIHPAIEQCVVASHRAASAMDKAPPPPWRAAFAAAGFAPVQATTFAESQAES
LLSKLGSASASACADAECHVAMVLYCPLIKERERKRWNVHLVIVLVELNTPQAEKNQREV
GGYGGEKNKILSEYNLLPLPVLEMQRKGGNHGVKRPCFTEMAAKHLRVRTGTSRTAAAT
LDGYTTLTWDSAPEWPWHRMHKPDRLRWAHWRVTGEPQCAAGAVAIGQASGHTGVRDT
AGANWQETCIVVTEMDVKLADKFRKKWYHSSKLQKRRCTVGLIKQDLKVQNQGGESE
ASRHKGLQISFRQVQISLRKPNRKCKHSGQTVKLINENCYYLSTKRNNLKIKEVNYICYIY
AEELHPLKVMFRNTQAKICQIDVQKS
>OsGRAS43
MGSSSLLLFPSSSSSATHSSYSPSSSSHAITSLLPPLPSDHHLLLYLDHQEQHHLAAAMVRK
RPASDMDLPPPRRHVTGDLSDVTAAAAPSSASAQLPALPTQLPAFHHTDMDLAAPAPPPPQ
QQVAAGEGGPPSTAWVDGIIRDIIASSGAAVSVAQLIHNVREIIRPCNPDLASILELRLRSLLT
SDPAPPPPPPPSHPALLPPDATAPPPPPTSVAALPPPPPPQPDKRRREPQCQEQEPNQPQSPKP
PTAEETAAAAAAAKERKEEQRRKQRDEEGLHLLTLLLQCAESVNADNLDEAHRALLEIAE
LATPFGTSTQRVAAYFAEAMSARLVSSCLGLYAPLPNPSPAAARLHGRVAAAFQVFNGISPF
VKFSHFTANQAIQEAFEREERVHIIDLDIMQGLQWPGLFHILASRPGGPPRVRLTGLGASME
ALEATGKRLSDFADTLGLPFEFCPVADKAGNLDPEKLGVTRREAVAVHWLRHSLYDVTGS
DSNTLWLIQRLAPKVVTMVEQDLSHSGSFLARFVEAIHYYSALFDSLDASYSEDSPERHV
VEQQLLSREIRNVLAVGGPARTGDVKFGSWREKLAQSGFRVSSLAGSAAAQAVLLLGMFP
SDGYTLIEENGALKLGWKDLCLLTASAWRPIQASGR
>OsGRAS44
MAGIAALANCQSDERSSSCGAALLEFMGPAAPPSEAAAAADDVVLPYISRILMEEDIDDD
MFFCLYPDHPALLEAQQPFAQILSSSSSIAGEVNSAPMEDSAALMMQGSGNGRGRKGSKH
GGDELEAEVGRASKLMATPEEEEDDDDGVGEMLEKMMLNGDEDEAFHGETNAPRVPAE
KKCGKAARRRRRQAKGEVVDLRELLMSCAQAVASGNRRSAGELLEQIKRHSSPTGDATE
RLAHYFADGLEARLAGAASLERRLVASAEERASAMELLEAYQVFMAACCFKWVAFTFAN
MAILRAAEGRNRLHIVDYGGQYHGLQWPSLLQRLAEREGGPPEFRAVAAARWETVTAED
VVGVDPDDEAAVVVNDVLSLGTLMDESGVFDDPSPRDTVLGSIRDMRPAVFVQAVVNGA
HGAPFFPTRFREALFFFSALFDMLGATTPEEGSHLRVVLERDVLRRAAVGVIAGEGAERVE
RPETYRRWQARNRRAGLRQAAVEGDVVEAVRRRVRRRHHEEFVIEEDAGWLLQGWKGR
ILYAHSAWVVAEDGAH
>OsGRAS45
MSASLHRPALASLLQIEAGGCGVLGVFAPGPRLPCRGYHPCRSSSTLGSASLTPRRWHISPP
GLRIRDVLVVHIVDLSCSAAHPWQWPKLLDDFHGRPGGAPELYLTVLHDDNDFLADMQS

LLSKKAESLGVSFHFISVIGRLETLDFSNLRSTFQIKFGVAVAISCALQMHRLLLVDDNLSST
SIAQLQKMANFTQPKQMASSVCSPASTLNYLQTPSPRTPKLLARLLSAIRALKPNIMLIME
QDADHNTLLFRDRFNEVLNYYAALFDCFHAVAAANPGRTDERLRVDRMILREEIKNILVC
EGVHRHERHERLDQWAMHMEESGFHNVQLSFSAIREAYVWQLKVQADNLRLCCTDRG
MFQDDMLSSATSSPASSVYSPSPSPSNGSWVQELSHDQQSVRLIGLLYQCAAEVSAGSFDR
ANLCLEHITQLASLDAPHALQRLAAVFADALARKLLNLILGLSRALLSSANSADAHLVPVA
RRHMFDVLPFLKLAYLTTNHAILEAMEGERFVHVVDFSGPAANPVQWIALFHAFRGRREG
PPHLRITAVHDSKEFLANMAAVLSKEAEAFDIAFQFNAVEAKLDEMDFDALRHDLGVRSG
EALAVSVVLQLHRLLAVDDGRRHAAAGCLTPVQIIARSSPRSFGELLERELNTRLQLSPDA
SVVSSLSPHSPAAATAAHPTTSTPKLGSFLSAVRSLSPKIMVMTEQEANHNGGAFQERFDE
ALNYYASLFDCLQRSAAAAAERARVERVLLGEEIRGVVACEGAERVERHERARQWAARM
EAAGMERVGLSYSGAMEARKLLQSCGWAGPYEVRHDAGGHGFFFCWHKRPLYAVTAWR
PAASRRGHTRS
>OsGRAS46
MEKATRCLARATGLAAAAAAGDGPRKRLAVAMVDCLARRLLRPVQAITDALIDPSVYLD
RRSVRAARRGFFELSPFPKVAFVVGNRAIVEAVENESLVHVVGMSGPFTQPCQWIQLLHEL
RRRPEGPPRVVRLTVVHDDGELLAKMEELVSDEAEELGMEFQFHGVVGQLEDLDFSNLR
NVLEIKSGEALVVSCTLQLHRLLAADDDAMYSSRSAHLNQMASIAQLQHMAVNSCPSSS
GGGSVQYKDDDPYRSPATPLTFVSPPVSTPHFQTPAALASFLSAVRALSPKILVVAEQDADH
NGVSFRKRFCEALHHYAAVFDSLDAAAATTSAASRLWSPDERAQVERVVVGEEIKGVLLR
DGAHRREWHDRLRQWAARMEMAGFTGVPLSYAAIKKGNDMVRRCGLRRCENKECGGC
LLLCWSSRPLYSISAWRPAASRGSGSGSERSEYVHVGAEPDDR
>OsGRAS47
MVLDSNELHQHDAPDVDVSINCDDRIFGQESVNLAAIQEELLEEDSLSDLLLAGAEAVEA
GDSILASVAFSRLDDFLSGIPENGAASSFDRLAYHFDQGLRSRMSSASTGCYQPEPLPSGN
MLVHQIIQELSPFVKFAHFTTNQAILDAIIGDMDVHVVDLNIGEGIQWSSLMSDLARCGGK
SFRLTAITTYADCHASTHDTVVRLLSEFADSLELPFQYNSICVHNEDELHAFFEDCKGSVIV
SCDTTSMYYKSLSTLQSLLLVCVKKLQPKLVVTIEEDLVRIGRGVSPSSASFVEFFFEALHH
FTTVFESMASCFIGSSYEPCLRLVEMELLGPRIQDFVVKYGSVRVEANASEVLEGFMACEL
SACNIAQARMLVGLFNRVFGVVFKKISLLMVYYISLGKNDLREPNKVIWSSLAAGCGSHG
IVVLAFYAADKLLEFKPKGIETYIHPAVERAHFHKEDRMWQEYEN
>OsGRAS48
MSHLENTLEARLAGTGSQMYQSLVAKRTSTVDFLKAYKLFTAACCVKKTIYNAVAGKRK
LHIVDYGLSYGFQWPALFFLLGTREGGPPEVRMTGIDVPQPGFRPADQIEETGRRLSICAR
APVRCAIQVPRHCRKVGDGRERGHGQGQPEPQRCGAQQQPRHEASRVRPEAPERAAGSC
CCRHRRAATALSTVVLDGLQIGLGISARCCLAGKQTSRSAPSSSPPRPSLHLHLHLRRRPPP
SSSRHAADDAALHPLSRRRDAYVERMGEGEVVDGIWPDGGGERRPAAVLALHRSARHR
SACHSQTAAALARCQPPASAGEKRRDATSAKPGKNTAEGPPVNGL
>OsGRAS49
MAGGGAKLQQQQAPTSPTASVSESNIVASTASADPEANDALAGLQALRFDGGGDIDDVEI
QSPDIALWESIFADQIGVSGAGADFLLSMSSAAASPRRDFMACSPKRDYMVTTSSPKRDY
MVTSSPKRDYMVSSPKREYMVTSPRREMATSPRRATFSNLYTSSHGGGGGGGHHLHHQS
YVHGGGMEGGGGGHGAQPQYGGLAGHGKGKAQSPLHKVYINNVGGGSGGGGVKSNTP
STLSCASSYVVHGGESGLPSLPSMDPFLEEGYLGSYQLPEKAAGGVGGGGGGDINRSGAS

VSVVTAPASSQLLPTLSECLAMPEPAAYRGGGDEAVAAAMAVAGELPVGAFVQPEMYYG
GGGEFGGEGMTPPLQHQMAADSSLHSMLGSVIQSEAEQEQDSGLQLVHLLLACADLVSK
GDHPAALRHLHLLRRVASPLGDSMQRVASHFADALAARLSLLSSPTSASPSPRAAAAAAPY
PFPPSPETLKVYQILYQACPYIKFAHFTANQAIFEAFHGEDRVHVVDLDILQGYQWPAFLQ
ALAARPGGPPTLRLTGVGHPPAAVRETGRHLASLAASLRVPFEFHAAAADRLERLRPAAL
HRRVGEALAVNAVNRLHRVPSSHLPPLLSMIRDQAPKIITLVEQEAAHNGPYFLGRFLEAL
HYYSAIFDSLDATFPAESTARMKVEQCLLAPEIRNVVACEGAERVARHERLERWRRLMEG
RGFEAVPLSAAAVGQSQVLLGLYGAGDGYRLTEDSGCLLLGWQDRAIIAASAWRC
>OsGRAS50
MAAAAPQLEELVDLEPFSPSLFLDLPPTPHSDDPNDDDLILPFISRMLMEDDIDDKFFYQFP
DHPALLHAQQPYAQILDAPSDDTTTNSSDDSASATTNNTTNSAAAANASWPYDPIELSQLL
QSPPHPVSDNHDADVGDTRSAPEDDKDLKLLFSAADNMEMLNMAFLKGREEANKLVPT
NNTLFAGFDGASLLKTEPAVDEPTLMFGRSGGSGRGRKNRHGEEDDLEAETGRSSKLMVP
PQEATAAASEMFDEIMFNWYEVIMKGMEELRVAMDSEAEKKARNGGGAGRRAARAKAA
VVDLHTLLIHCAQAVATSDRRSATELLKQIKQNSSARGDATQRLACCFAEGLEARLAGTGS
QVYKSLVAKCTSTVDFLKAYKLFAAACCIKKVSFIFSNKTILDAVAGKRKLHIVDYGLSYG
FQWPGLFKCLSEREGGPPEVRITGIDFPQPGFRPADQIEETGRRLSNCARQFGVPFRFQAIA
AKWETVRREDLHLDREEEEEEEEEVLVVNCLHFLNALQDESVVVDSPSPRDMVLNNIRD
MRPHVFVQCVVNGAYGAPFFLTRFRETLFFYSSQFDMLDATIPRDNDERLLIERDILGRWA
LNVIACEGADRVDRPETYKQWLVRNHRAGLTQLPLQPQVVELVRDKVKKLYHKDFVIDV
DHNWLLQGWKGRILYAMSTWVADRDHKSLF
>OsGRAS51
MAAAPKPEELVVAIEQPFSPSLFLDLPPTPHHDDDPNNVNDDLLLPFISRILMEDDIDDKFF
YQFPDHPALLQAQQSYAQILHAPATSSSSDDTTINNNTTNSTSVPDTLAMPDHDADTQSAP
DDMEMLYMAFLKGREEATKFLPTNNTLFSGFKAEPVLDIQPTFTFGPSGGGRGRKNRHAE
EDDLETETSRSSKLMAPEHDDAAAADEIFDEIILNGYQMIIKGIDELRVAMGSQSQADKNG
RRASRAKTAVVDLHTLLIHCAQAVATGDWRSATELLKQIKQNSSARGDATQRMACCFAEG
LEARLAGTGSQMYQSLVAKRTSTVDFLKAYKLFTAACCIKKVSVIFSNKTIYNAVAGRRKL
HIVDYGLSYGFQWPALFFLLGAREGGPPEVRMTGIDVPQPGFRPADQIEETGRRLSICARQ
FGVPFKFRAIAAKWETVRREDLHLDPEEEEEEVLVVNCLHGLNTLQDESVVVDSPSPRDV
VLDNIRDMRPHVFVQCVVNGAYGAPFFVTRFREALFFYSAHFDMLDATIPRDNDDRLLIE
RDMLGRCALNVIACEGADRVDRPETYKQWQVRNHRAGLRQLPLEAEVVELVRGKVKSL
YHKDFVIDVDHNWLLQGWKGRILYAMSTWVAHHP
>OsGRAS52
MATTPEAAESLRFRWPAAEEEFDNDMVLPYISRLLMEDDVHDHFFYQYPDHPALLRAQQP
FAQILASSPSSAAGASSSSSSSDAPPSRPFFDDEAATAKTFPTAAVHSVDHQYSGGLDMVN
MAFLKGMEEANKFLPTNTLLLSTDSSTTLQLQVQGEVVVDGHGMLGGVGGAAAAHAH
GAINSKKVNCRDDDLEAGTGRATKLMAPEPELEEEGARQMFDEMMLQEHEICMKGVKQ
LSLKSKSSSSKKARGRRTVIHTEPVDLHNLLLHCAQAVATDDRRSAHELLRQIKQHSSAWG
DAGQRLAHCFAQGLEARLAGTGSQVYQSLMSQRTSVVDFLKAYRLYMEACCCKKVAFV
FSNKTIYDAVAGRRKLHIVDYGLSYGFQWPGLLRELAARRGGPPEVRITGIDLPQPGFRPD
QHIEETGRRLSRYADELGVPFKFHGIAATKKESVRREELGEAEEDEVVVVISLCHFRNVMD
ESLQEDSSRSPRDEVLGNIRRMRPDVFIHGIMNGAYGATYFLTRFREALYYYAAQFDLLDA
TVGRESHERMLVERDIFGRAALNVIACEGAERVERPEMYKQWQARNQRAGLRQLPLNPQ

VVRLVLDKVRDKYHKDFVVDEDQRWLLHRWKGRVLYALSTWVAQH
>OsGRAS53
MANPEDFFWEALLKENEAPSPSPVFFELPPTPLANSDGSTDPSSLLDNQLLSYVTSMLMED
EMGSSAAVTNLQCVNRGSTEEANNMLPGSEVVRAFLKGMEEASKLLPRNNSFRMLETVD
QVSSHGHCRGGKKKNHDRDEQQLEEELGRSSKLAAMTNAGTEEAGARELLDELMLHSH
ETCIKDMEKLRIDMDNEADKTIKKKGKKGSSSKVVDLRMLLIQCAQAMATDNQQSAGEL
LKKIKQHALATGDAMQRVAHYFAKGLEARLAGSGKHLYQNHVRMSLVEYLKVYKLYMA
ACCFKKVALMFAAMTIMQAVQGKKRLHIVDYGIRCGLHWPDLFRRLGSREDGPPEVRITI
VDIPQPGFRPFQRIEAAGHCLSSCANEFRVPFRFQAVVAAKWETVGAEDLHIEPDEVLVVN
DLWSFSALMDESIFCDGPNPRDVALRNISKMQPDVFIQGIINGGYGASFLSRFRGALLYYSA
LFDMLDATTPRESGLRLALEQNVLGPYALNAIACEGADLVERPEKYRQWQARNHRAGM
QQLKLRPDIVDTIREEVNKYHHKDFLLGEDGQWLLQGWMGRVLFAHSAWVPQQQDNSS
G
>OsGRAS54
MANPEDFFWEALLKENEAPSPPPVFFDLPATPLSNSDGTDPSSLDNQLLSYVSRMLMEDE
MGSSAAITNLQCVNRGSTEEANNMLPGSEVVRVFSKGMGEASKLLPRNNSFRTLETVDQ
VSSDGHCRGRKKKNHDRDEQQLEEELGRTSKLAALTIAGTQEAGARELLDELMLHAHET
CIKDMEKLRIDMDNEAEKKINKKDKKGSSSKVVDLRLLLIQCAQATATDNQQSAGELLKK
IKQHALATGDAMQRVAHYFAKGLEARLAGRGKHLYQNQMRMSLVEYLKVYKLYMAAC
CFTKVALMFAAMTIMQAVQGKKRLHIVDYGPRCGLHWPDLLRRLGSREDGPPEVRITIVD
ILQPAFRPFQRIEEAGHCLSSCANEFRVPFRFQAVAAAKWETVGAEDLHIEPDEVLVVNDL
WSFSALMDESVFCDGPNPRDVALRNISKMQPDVFIQGITNDSYGASFLSRFRAVLLYYSAL
FDILDATTPRDSGLRLALEQNVLGPYALNAIACEGADLVERPEKYKQWQARNHRAGMQQ
LKLRPDIVDTIRDEVNKYHHKDFLLGEDGQWLLQGWMGRILFAHSAWVRQSQDTSSG
>OsGRAS55
MGSSSLLLFPSSSSSATHSSYSPSSSSHAITSLLPPLPSDHHLLLYLDHQEQHHLAAAMVRK
RPASDMDLPPPRRHVTGDLSDVTAAAAGAPTLSASAQLPALPTQLPAFHHTDMDLAAPAP
PAPQQVAAGEGGPPSTAWVDGIIRDIIASSGAAVSVAQLIHNVREIIRPCNPDLASILELRLRS
LLNSDPAPPPPPPSHPALLPPDATAPPPPPTSVAALPPPPPAQPDKRRREPQCQEQEPNQPQSP
KPPTAEETAAAAAAAAAAAAAAAKERKEEQRRKQRDEEGLHLLTLLLQCAESVNADNL
DEAHRALLEIAELATPFGTSTQRVAAYFAEAMSARLVSSCLGLYAPLPSPSPAGARVHGRVA
AAFQVFNGISPFVKFSHFTANQAIQEAFEREERVHIIDLDIMQGLQWPGLFHILASRPGGPP
RVRLTGLGASMEALEATGKRLSDFADTLGLPFEFCPVADKAGNLDPEKLGVTRREAVAVH
WLRHSLYDVTGSDSNTLWLIQRLAPKVVTMVEQDLSHSGSFLARFVEAIHYYSALFDSLD
ASYSEDSPERHVVEQQLLSREIRNVLAVGGPARTGDVKFGSWREKLAQSGFRVSSLAGSA
AAQAALLLGMFPSDGYTLIEENGALKLGWKDLCLLTASAWRPIQASGR
>OsGRAS56
MGPAAPPSEAAAAADDVVLPYISRILMEEDIDDDMFFCLYPDHPALLEAQQPFAQILSSSSG
IAGEVNSAPMEDSAALMMQGSGNGRGRKGSKHGGDELEAEVGRASKLMATPEEEDDDD
DGVGEMLEKMMLNGDEEMNAPRVPAEKNGGKAARRKRRQGKGEVVDLRELLMSCAQA
VASGNRRSAGELLEQIKRHSSPTGDATERLAHYFADGLEARLAGAASLEHRLLASAEERA
SAMELLEAYQVFMAACCFKWVAFTFANMAILRAAEGRSKVHIVDYGGQYHGLQWPSLL
QRLAEREGGPPEVRMTLVGHPQPGFRPARRLERTGRRLSNCARAFGLPFKFRAVAAARWE
TVTAEDVVGVDHDEAAVVVNDVLSLGTLMDESGVFDDPSPRDTVLGSIRDMRPAVFVQA

VVNGAHGAPFFPTRFREALFFFSALFDMLDATTPEEGSHLRAVLERDVLRRAAVGVIAGE
GAERVERPETYRRWQARNRRAGLRQVAVEADVVEAVRRRVRRRHHEEFVIEEDAGWLLQ
GWKGRILYAHSAWVVAEDATSVGELTVVTTSLVAIILSAASAQDELPLSTNE
>OsGRAS57
MMHGLWVQDQGMVDHLAQLVPLLHECASHVTEGSFEKADFSFKKIRMLTIADGPLQHLS
KIIVDSLDHRLLSSIQGLYGALINPSDYFEKSTLPGCPAHNFFKLNPYLSTGFVTINRAIMEA
MEDEKNFLEIKVKSNLCSILKLSHYNFKTLQGAIYVERSLMFNMVELQVVHIVDLSCSAA
HPWQWLKLLDDFHGRPGGAPELYLTVLHDDNDFLAEMQSLLSKKAESLEVSFRFISVIGR
LETLDFSNLRSTFQIKFGVAVAISCALQMHRLLLVDDNLSSTSIAQLQKMANFTQPKQMAS
SVCSPASTLNYLQTPSPRTPKLLARLLSAIRALKPNIMVIMEQDADHNALLFRDRFNEVLN
YYAALFDCFHAVAAANPGRTDERLRVERMILREEIKNILVCEGVHRHERHERLDQWAMH
MEESGFHNVQLSFSAIREGKENLLSFGLKNCQNKEDRGCLLLSWGSTNLYSISLRLCCTDR
GMFQDDMLSSATSSPASSVYSPSPSPSNGSWVQELSHDQQSVRLIGLLYQCAAEVSAGSFD
RANLCLEHITQLASLDAPHALQRLAAVFADALARKLLNLIPGLSRALLSSANSADAHLVPV
ARRHMFDVLPFLKLAYLTTNHAILEAMEGERFVHVVDFSGPAANPVQWIALFHAFRGRRE
GPPHLRITAVHDSKEFLANMAAVLSKEAEAFDIAFQFNAVEAKLDEMDFDALRHDLGVRS
GEALAVSVVLQLHRLLAVDDGRRHAATGCLTPVQIIARSSPRSFGELLERELNTRLQLSPD
ASVVSSLSPHSPAAATAAHPTTSTPKLGSFLSAVRSLSPKIMVMTEQEANHNGGAFQERFD
EALNYYASLFDCLQRSAAAAAERARVERVLLGEEIRGVVACEGAERTERHERARQWAAR
MEAAGMESVGLSYSGAMEARKLLQSCGWVGPYEVRHDAGGHGFFFCWHKRPLYAVTA
WRPAASRRGHTRS
>OsGRAS58
MEKATRCLARATGLAAAAAAGDGPQKRLAEAMVDCLARRLLRPVQAITDALIDPSVYLD
RRSVRAARRGFFELSPFPKVAFVVGNRAIVEAVENESLVHVVGMSGPFTQPCQWIQLLHEL
RRRPEGPPRVVRLTVVHDDGELLAKMAEVLSDEAEELDMEFQFHGVVGQLEDLDFSNLR
DVLEIKSGEALVVSCTLQLHRLLAADDDAMYSSRSAHLNQMASIAQLQQMAVSSCPPSTG
GGGSVQYKDDDDDPYRSPATPLTFVSPPASTPHLQMPAALANFLSAVRALSPKIVVVAEQD
ADHNGVSFRKRFCEALHHYAAVFDSLDDAAAATTSAASHLWSPDERAQVERVVVGEEIK
GVLLRDGAHRRERHDRLRQWAARMEMAGFTGVPLSYAAIRKGNDMVRRCGLRRCENR
ECGGCLLLCWSSRPLYSISAWRPAASGGAGSGSERSEYIHVGAEADDR
>OsGRAS59
METMSYPCSLLIPFSTQFEEISSSSLLLWSPQAEENPHENANMYEFDADHSHDQIHQDHQF
LDMMVIQESANEFDGNHSHDQIHQDHEFLETMVIQESANEFDGDHSHDQIHQDHEFLEM
MAIQESANDLLQLQDDFSVPNADPLAASFEFDERLAVAGHENGNVVATQEESAGDLLLAG
AMAVDAGDAVHASAIMSRLDDLLADIAGRRSCEATSPVDHLAYYFARGLKLRISGAATPA
SSPPPPAANWSSPAYRMLQELTPFVKFAHFTANQAILEATADDLDVHVVDFNVGEGVQWS
SLMLKLLLLGTITILQPKLVILIEDELSRISKNPPSPSLAAPPPFPEFFSDAVAHFTAVMESTAS
CLVSYDDEAWLSLRRVGEEVVGPRVEDAVGRYGSLAGGAQMMEGLRAREVSGFSVAQG
KMLAGLFGGGFGVVHQEKGRLALCWKSRPLISVSLWCPK
>OsGRAS60
MGSSAADSFPACGDDAIRDVYGIGGGGEEDDPSLFLYLSDLAPVSPSAYLDLPPSPPPPTTT
ATTMVKEGEEAPEDLVLPFISRMLMEEDIDDKFFYDYPDNPALLQAQQPFLEILSDPSSNSR
SSNSDDPRLSPTSSSDTSAAINSYDAAATATAVAAAAVPVPQYESIELDPAAFFAAANSDLM
SSAFLKGMEEANKFLPTENKLIIDLEASSENNYLRGLEEAKRFLPSDDKLQVGFAAAAAPV

VSVKKEAVDVVVATASGGGGRGRKNPYDDEELELEGGRSSKQTAVQGDDVAARAMFDK
VMMPSHENCTEMMEKLRIAMKEEAAKNEASAGGKGGNGKVKGGRRGGRDVVDLRTLL
IHCAQAVATDDRRSATELLKQIKQHAKPTGDATQRLAHCFAEGLQARIAGTGSLVHQSLVA
KRTSAVDILQAYQLYMAAICFKKVSFIFSNQTIYNASLGKKKIHIVDYGIQYGFQWPCFLRR
ISQREGGPPEVRMTGIDLPQPGFRPTERIEETGHRLSKYAQEFGVPFKYNAIAAVKMESVR
KEDLNIDPDEVLIVNCQYQFKNLMDESVVIDSPRDIVLSNIRKMQPHVFIHAIVNGSFSAPF
FVTRFREALFFYSALFDVLDATTPRESEQRLLIEQNIFGRAALNVIACEGIDRVERPETYKQ
WQVRNQRAGFKQLPLNPEIVQVVRNKVKDCYHKDFVIDIDHQWLLQGWKGRILYAISTW
TPNDALSYF
