# Supplementary material for: Genome-wide identification and expression analysis of the GRAS gene family in Dendrobium chrysotoxum
Source: Front Plant Sci. 2022 Nov 28;13:1058287. doi: 10.3389/fpls.2022.1058287 (PMC9742484; doi:10.3389/fpls.2022.1058287)
Supplement: Supplementary Figure 2 — Sequence and logo of Motif1–10; [file Table_2.pdf]

Motif1	Sequence	FYEASPYLKFGHFTANQAILEAFEGERRVHIIDFDIKQGLQWPSLLQALA
	Logo	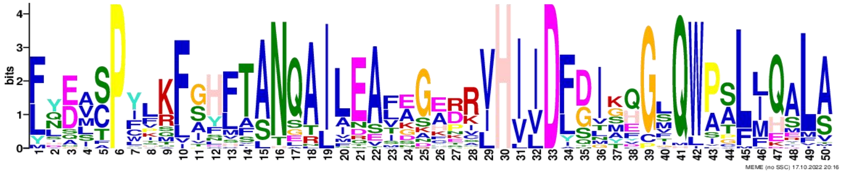
Motif2	Sequence	FLERFMEALHYYSALFDSLEA
	Logo	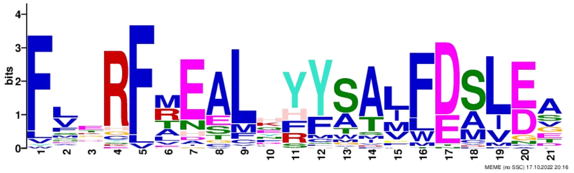
Motif 3	Sequence	LASPTGDPMQRLAAYFAEALAARJ
	Logo	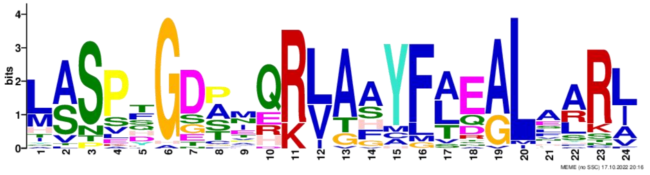
Motif 4	Sequence	LGREICNIIACEGAERVERHE
	Logo	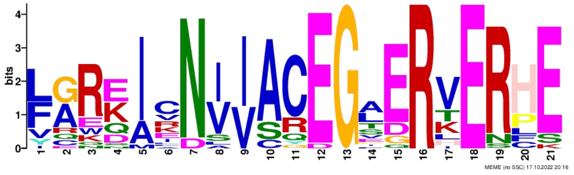

Motif 5	Sequence	LVLGWKGRPLISASAWR
	Logo	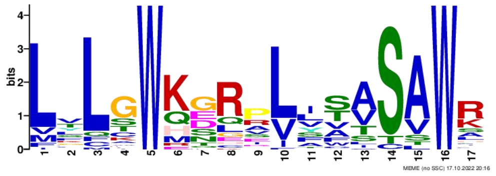
Motif 6	Sequence	QWRERMERAGFEPVGLSSFAVKQAKLLLK
	Logo	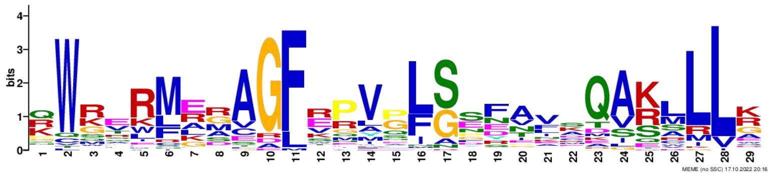
Motif 7	Sequence	TGRRLADFAESLNVPFEFHAVV
	Logo	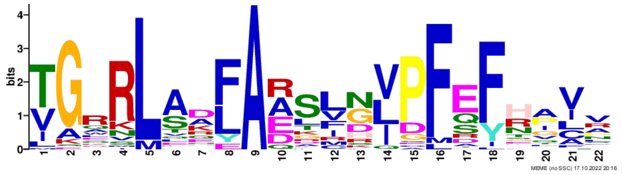
Motif 8	Sequence	FLRLVRSLRPRIVTVVEZEAB
	Logo	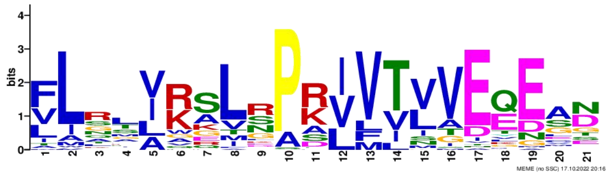

Motif 9	Sequence	ESLRVKEGEALAVNCVLQLHKLLDESGAT
	Logo	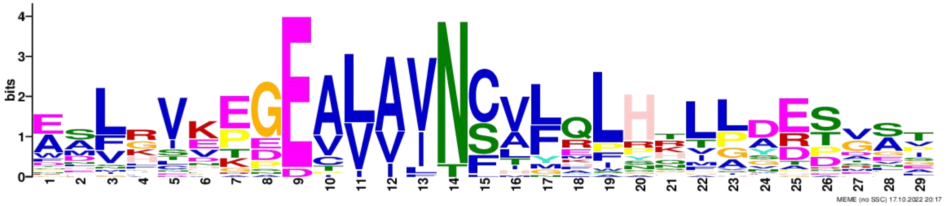
Motif 10	Sequence	AGLRLVHLLLACAEAIAAGBL
	Logo	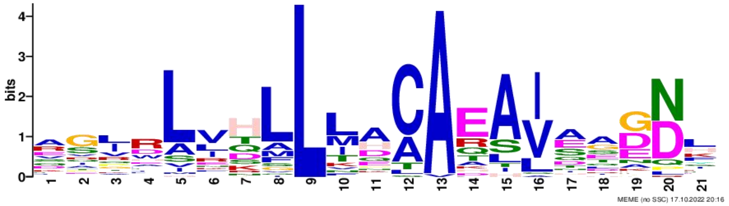
