# Supplementary material for: Genome-wide identification and expression analysis of the GRAS gene family in Dendrobium chrysotoxum
Source: Front Plant Sci. 2022 Nov 28;13:1058287. doi: 10.3389/fpls.2022.1058287 (PMC9742484; doi:10.3389/fpls.2022.1058287)
Supplement: Supplementary Figure 4 — Plant images of D. chrysotoxum before and after different abiotic stresses; [file Table_4.pdf]

| **Supplementary Figure 4**. Plant images of *D. chrysotoxum* before and after different abiotic stresses | | |
|---|---|---|
| | **Before** | **After** |
| **Group-A**<br>High temperature stress<br>(16h light/8h dark, 30°C/38°C, water once every 24h, treatment 48h.) | 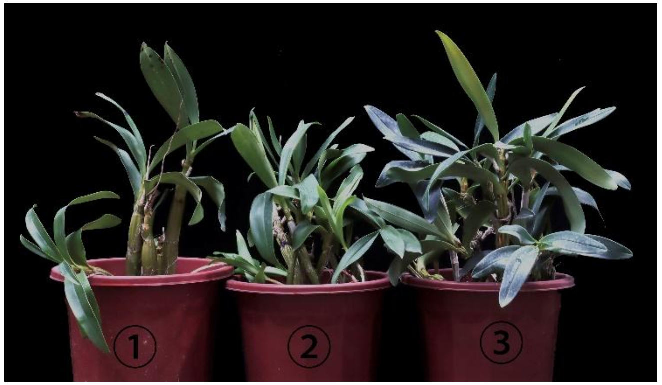 | 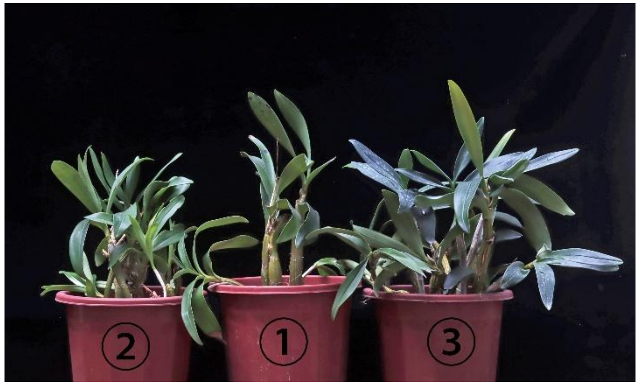 |
| **Group-B**<br>Salt stress<br>(16h light/8h dark, 15°C/25°C, irrigated with 0.5 M NaCl solution and plant roots every 8h, treatment 48h..) | 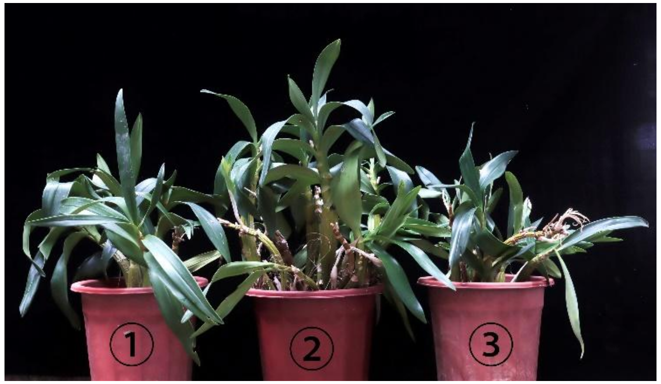 | 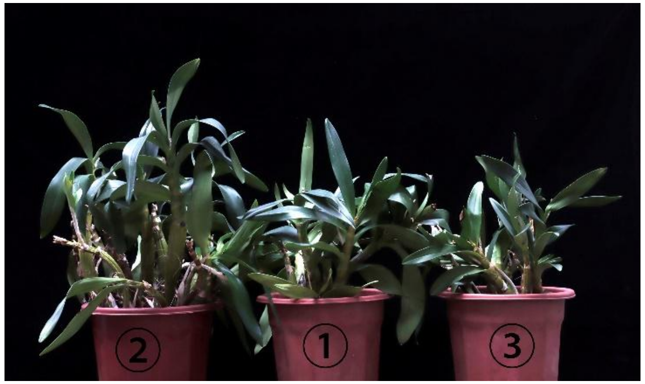 |
| **Group-C**<br>Drought stress<br>(16h light/8h dark, 15°C/25°C, treatment ten days) | 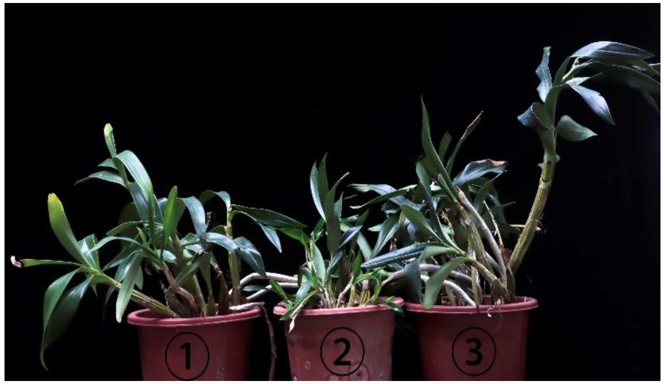 | 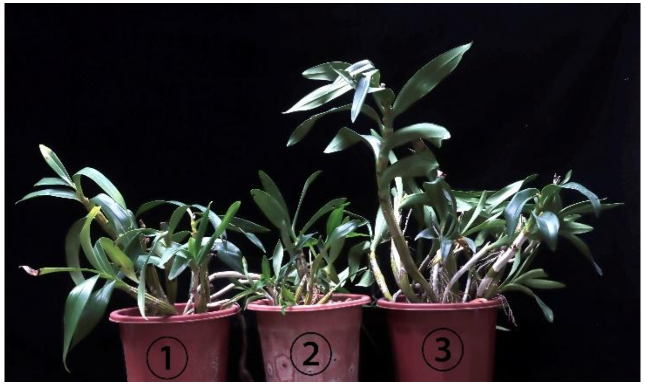 |
